# Utility of constraints reflecting system stability on analyses for biological models

**DOI:** 10.1371/journal.pcbi.1010441

**Published:** 2022-09-09

**Authors:** Yoshiaki Kariya, Masashi Honma, Keita Tokuda, Akihiko Konagaya, Hiroshi Suzuki

**Affiliations:** 1 Department of Pharmacy, The University of Tokyo Hospital, Faculty of Medicine, The University of Tokyo, Bunkyo-ku, Tokyo, Japan; 2 Department of Computer Science, University of Tsukuba, Tsukuba, Ibaraki, Japan; 3 Molecular Robotics Research Institute, Limited, Kyowa Create Dai-ichi, Minato-ku, Tokyo, Japan; University at Buffalo - The State University of New York, UNITED STATES

## Abstract

Simulating complex biological models consisting of multiple ordinary differential equations can aid in the prediction of the pharmacological/biological responses; however, they are often hampered by the availability of reliable kinetic parameters. In the present study, we aimed to discover the properties of behaviors without determining an optimal combination of kinetic parameter values (parameter set). The key idea was to collect as many parameter sets as possible. Given that many systems are biologically stable and resilient (BSR), we focused on the dynamics around the steady state and formulated objective functions for BSR by partial linear approximation of the focused region. Using the objective functions and modified global cluster Newton method, we developed an algorithm for a thorough exploration of the allowable parameter space for biological systems (TEAPS). We first applied TEAPS to the NF-κB signaling model. This system shows a damped oscillation after stimulation and seems to fit the BSR constraint. By applying TEAPS, we found several directions in parameter space which stringently determines the BSR property. In such directions, the experimentally fitted parameter values were included in the range of the obtained parameter sets. The arachidonic acid metabolic pathway model was used as a model related to pharmacological responses. The pharmacological effects of nonsteroidal anti-inflammatory drugs were simulated using the parameter sets obtained by TEAPS. The structural properties of the system were partly extracted by analyzing the distribution of the obtained parameter sets. In addition, the simulations showed inter-drug differences in prostacyclin to thromboxane A2 ratio such that aspirin treatment tends to increase the ratio, while rofecoxib treatment tends to decrease it. These trends are comparable to the clinical observations. These results on real biological models suggest that the parameter sets satisfying the BSR condition can help in finding biologically plausible parameter sets and understanding the properties of biological systems.

## Author summary

We propose a new method to analyze the properties of biological dynamic models, which we named TEAPS (Thorough Exploration of Allowable Parameter Space). TEAPS can thoroughly determine combinations of parameter values for ordinary differential equations with which an initial state in a certain range converges to a particular fixed point. This stable and resilient behavior is a characteristic shared with many biological systems, including metabolic systems and intracellular signaling systems. Therefore, this thorough search outlined the possible parameter space as biological systems for target models, which helps to understand the system constraints when the target systems behave dynamically. The obtained parameter space can be used as an initial space for parameter tuning. For models that include a large number of parameters, the parameter space to be searched in the parameter tuning process is too large; therefore, narrowing down the space by TEAPS potentially contributes to the analysis of the dynamics of complicated biological models. Thus, our approach can partly overcome the current problem in parameter tuning and can advance the computational dynamic analyses of biological systems.

## Introduction

To understand and predict the dynamic behaviors of a complex biological system such as a metabolic [1–3] or a signaling [4,5], *in silico* simulation with a mathematical model consisting of multiple ordinary differential equations (ODEs) can be helpful. Nevertheless, the utility of this approach is limited due to the difficulties in determining kinetic parameter values [6]. In some cases, experimental measurements can estimate the parameter values directly [7], although we should be cautious when adopting the measured values. Previous reports have indicated that the kinetic parameter values determined *in vitr*o using purified recombinant enzymes are not in good agreement with the values estimated under *in vivo* conditions using fluorescence cross-correlation spectrometry, partly due to the molecular crowding effects in the intracellular compartments [8,9]. These findings also indicate the risk of reusing the kinetic parameter values from an existing model describing the same reaction under different conditions such as a different organelle, cell, or tissue. In certain cases, the measurement of parameter values describing microscopic molecular behavior is rather difficult [8]. When the model includes too many kinetic parameters, it is impractical to estimate all the parameter values experimentally. Another way to determine the kinetic parameter values is by fitting the model to the observed data of model variables [5]. Currently, various fitting algorithms are available in cases where the flexibility of a model can be restricted by a sufficiently large number of data points and sufficiently narrow exploration ranges of parameter values [10,11]. However, molecular biology is advancing continuously, and the number of components to be incorporated in a model increases. Thus, it becomes more difficult and time-consuming to determine the exact values of all the parameters as the model size increases, the allowable parameter space expands, and the required number of data points becomes larger. The difficulty in determining parameters is directly related to the reliability of the simulation results. The vast parameter space will result in the risk of overfitting the model to the data and missing other possible parameter values that also explain experimental results, leading to misinterpretation of the system behavior. However, an ODE-based simulation is necessary in such cases, since experimental observations of numerous variables are usually time and labor intensive.

**Fig 1:**
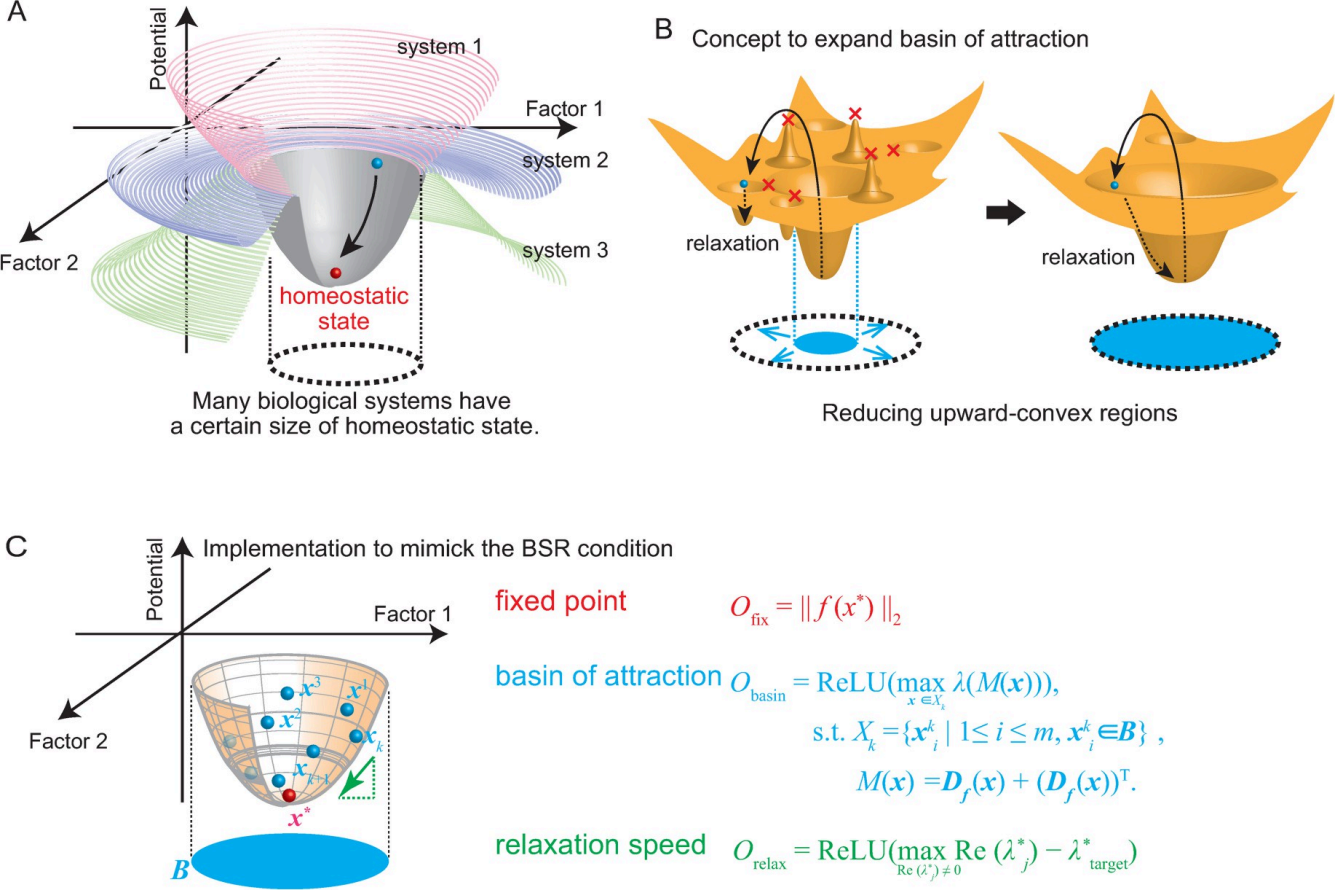
Model of biological system at homeostatic state. (A) Many biological systems behave around a certain stable state. Such stable behavior can be observed in many biological systems although the size of the stable region around the stable state differs by systems. (B) Assumed structure of biological stable region. If there are upward-convex regions in potential focused region, the size of the basin of attraction is limited, therefore, our concept is to diminish such regions in parameter searching. (C) Three factors are considered to search the parameter sets satisfying the BSR condition and incorporated in the objective function to minimize.

Based on these observations, our motivation in the present study is to obtain as much information as possible regarding the model dynamics even in cases where the parameter values are hardly determined. We propose an alternative parameter search process by adopting the following two ideas. First, we decided to focus on general characteristics shared by many biological models. Concentrations of various metabolites in each tissue are maintained in the absence of an extrinsic stimulus, such as the food intake, but they change in response to the stimulus and are then restored again to the basal levels after a certain period of time in the absence of the stimulus [12]. Similarly, an intracellular signaling pathway is activated in response to ligand stimulation and then restored to the inactivated state after the removal of the stimulator [13]. These properties of stability and resilience are observed in many biological systems in a homeostatic state [14,15]. One of our key ideas is to use these properties in the parameter searching process. If a system of interest can be assumed to be biologically stable and resilient (BSR), the size of parameter space to be focused is significantly reduced to satisfy this constraint. Although there are exceptions, the BSR feature is widely shared by various biological systems and this constraint is applicable to solve many problems (Fig 1A). Second, we decided to avoid exact determination of the optimal kinetic parameter set, a series of parameter value combinations, and rather aimed to determine the whole subset in the parameter space where the model satisfies the BSR constraint. It is often the case that the amount of observed data is insufficient to fit the model. In addition, some kinetic parameters are not sensitive enough to the observable variables due to the system structure, and the parameter values cannot be determined with acceptable estimation accuracy [16]. Thus, we adopted an approach to collect as many parameter sets as possible, thereby satisfying the conditions on the system outputs and BSR constraints. For this purpose, to enable handling more complicated models, we modified the global Cluster-Newton method (CNM), which was developed to obtain multiple parameter sets satisfying some conditions from entire parameter space without high computational cost [17]. In the present study, we evaluated the ability of our proposed method to estimate the allowable parameter space of a kinetic model and also evaluated the utility of the acquired parameter sets to extract some features of a system of interest. The proposed method will contribute to a better understanding of the properties of a biological system with a known network structure but unknown kinetic parameter values.

## Results

### Formalization of the BSR property of a biological system

In this section, we define the BSR system as a dynamical system that satisfies the following two conditions: first, the system has a stable fixed point and operates around it, and, second, the system returns close to the fixed point within a specific time after moderate external perturbation to the state variables (Fig 1A). We consider a system satisfying the following ordinary differential equation:

$$\frac{dx}{dt}=f(x,\mu )={\left(f_{1}(x,\mu ),f_{2}(x,\mu ),\cdots ,f_{N_{phase}}(x,\mu )\right)}^{T},$$ (1)

where $x(t)={\left(x_{1}(t),x_{2}(t),\cdots ,x_{N_{phase}}(t)\right)}^{T}$ represents the state of the system, such as the concentrations of metabolites, and $\mu \in R^{N_{p}}$ is the parameter. Hereafter, we formulate the two above-stated conditions of the BSR system.

There exists $x^{*}\in R^{N_{phase}}$ such that

$$f(x^{*})=0,$$ (2)

$$\mathrm{If}\;\varepsilon \ll \|x(t_{0})-x^{*}\|\leq r_{max},\;then\;(min\;\Delta t\;s.t.\|x(t_{0}+\Delta t)-x^{*}\|<\varepsilon ){∼\Delta t}_{conv}$$ (3)

where ‖ ‖ denotes norm, x* is a fixed point, *r*_max_ is the distance defining the tolerable range (size of a stable area around the fixed point), and *ε* is the distance between the fixed point and the state of the system, with which the state is presumed to be close to the fixed point. Further, $min\;\Delta t\;s.t.\|x(t_{0}+\Delta t)-x^{*}\|<\varepsilon$ is the minimum time to return nearer than *ε* to the fixed point, *a*~*b* indicates *a* and *b* have the same order of magnitude, and Δ*t*_conv_ specifies the timescale of the system to return significantly close to the fixed point.

### Design of the objective function to achieve the BSR condition

Next, we designed our objective function in the parameter space to identify the parameter sets with which the system satisfied the above-defined BSR condition.

First, we considered the condition in which the system had a fixed point at x* (Eq 2). Finding the root of the following objective function yields the condition stated in Eq 2:

$$O_{fix}(\mu )={\left\|f(x^{*},\mu )\right\|}_{2}.$$ (4)

where ‖ ‖_2_ denotes L2 norm.

Next, we considered the conditions stated in Eq 3. This condition has two requirements: (i) the orbits starting at initial conditions in the specified region must converge to x*, and (ii) the orbits must converge to a fixed point within a specific timescale. Menck *et al*. conceptualized the former point as the “basin stability,” and they proposed quantifying it by calculating the ratio of the initial conditions that converge to the attractor [18]. In principle, one can search for parameters satisfying these two conditions if the dependency of the ratio of the converging orbits and the converging time on the parameters is known. However, the duration for different initial conditions in the phase space of a high-dimensional nonlinear system is practically difficult to calculate without solving the differential equation through numerical simulations. Incorporating multiple numerical simulations for different initial conditions into each iteration step in the parameter optimization process involves a high computational cost. Thus, rather than directly evaluating the parameter dependency of the basin stability and convergence time, we used a different approach: to construct objective functions that could be calculated without numerical simulations of the system. We implemented the following objective function whose optimization is expected to yield an expansion of the basin stability in region ***B***:

$$O_{basin,X^{\left(k\right)}}(\mu )=ReLU\left(\underset{x\in X^{\left(k\right)}}{max}\lambda (M(x))\right),$$ (5)

$$X^{\left(k\right)}=\{x_{m}^{\left(k\right)}|1\leq m\leq N_{obs},x_{m}^{\left(k\right)}\in Β\},$$ (6)

$$M(x)=D_{f}(x)+{\left(D_{f}(x)\right)}^{T},$$ (7)

where ***D***_***f***_(***x***) is the Jacobian matrix of ***f***(***x***) at ***x***, *M*(***x***) is the sum of ***D***_***f***_(***x***) and its transpose, ***B*** is the pre-determined stability region, *λ*(*M*(***x***)) is the eigenvalue of the matrix *M*(***x***), and ReLU(x)≝max(0, x). Further, ***X***^(*k*)^ is a finite set of points randomly chosen from ***B*** at iteration number *k*, which we refer to as “observation points” hereinafter, and *N*_obs_ is the number of observation points. Note that all the eigenvalues of matrix *M*(***x***) are real numbers because *M*(***x***) is symmetric. The observation points ***X***^(*k*)^ were sampled repetitively during optimization (S1 Fig). The condition max ReLU(*λ*(*M*(***x***))≤0 indicates that the distance between the neighboring orbits in the phase space does not expand at ***x***. We assume that it is quite natural to expect the basin stability of a given fixed point to increase when the local contraction of the neighboring orbits is assured at a large number of points surrounding the fixed points, given that ***f***(***x***, ***μ***) is described by a smooth parametric function with parameters $\mu \in R^{N_{p}}$. Actually, we can prove that if the maximum eigenvalue of *M*(***x***) is smaller than 0 in a disc surrounding a fixed point, any orbit starting at the initial condition on this disc converges exponentially to this fixed point (see S1 Information for the proof).

Finally, we designed a function to reflect the time interval required for the convergence of the state of the system toward a fixed point. We focused on the characteristic timescale of relaxation around the fixed point, which is determined by the largest real part of the eigenvalues of the Jacobian matrix at the fixed point. Because we focused on biological systems that have smooth behavior around the fixed point, we expect that the relaxation timescale around the focused region is similar to that of the fixed point. Thus, given the system’s desired relaxation timescale Δ*t*_conv_ (Eq 3), our idea is to include the slowest relaxation at the fixed point in the objective function as follows:

$${O^{*}}_{relax}(\mu )=ReLU(\max\;Re(\lambda^{*})-{\lambda^{*}}_{target}),$$ (8)

where max Re(λ*) is the largest real part of the eigenvalues of the Jacobian matrix of ***f***(***x***) at x*, and λ*_target_ = −1/Δ*t*_conv_ is a negative constant value that defines the standard of the slowest relaxation speed. In biological experiments, the measured values often vary within a certain range despite performing experimental measurements for multiple sets in the same stable condition. This might be caused by measurement errors; however, existence of multiple stable states may form a neutrally stable region around the measured value. Thus, we allowed the existence of zero eigenvalue(s) and implemented the following objective function using non-zero eigenvalues:

Orelax(μ)=ReLU(maxRe(λ*)≠0Re(λ*)−λ*target). (9)

This objective function allows the largest real parts of the eigenvalues to be optimized to 0. Therefore, the change of the system’s state in certain directions does not cause it to return toward the fixed point. Even under this setting, the system would still be stable as per Lyapunov, which is often regarded as an indicator of biological stability [19,20].

Finally, the weighted sum or the concatenated vector of *O*_fix_(***μ***), $O_{basin,X^{\left(k\right)}}(\mu )$ and *O*_relax_(***μ***) were utilized as the single objective functions to optimize and yield parameter sets that satisfy the BSR condition. Considering that the objective function values of *O*_fix_(***μ***), $O_{basin,X^{\left(k\right)}}(\mu ),$ and *O*_relax_(***μ***) were different terms mathematically, the weight needs tuning.

### The approach to unveil parameter space satisfying BSR and its advantages

**Fig 2:**
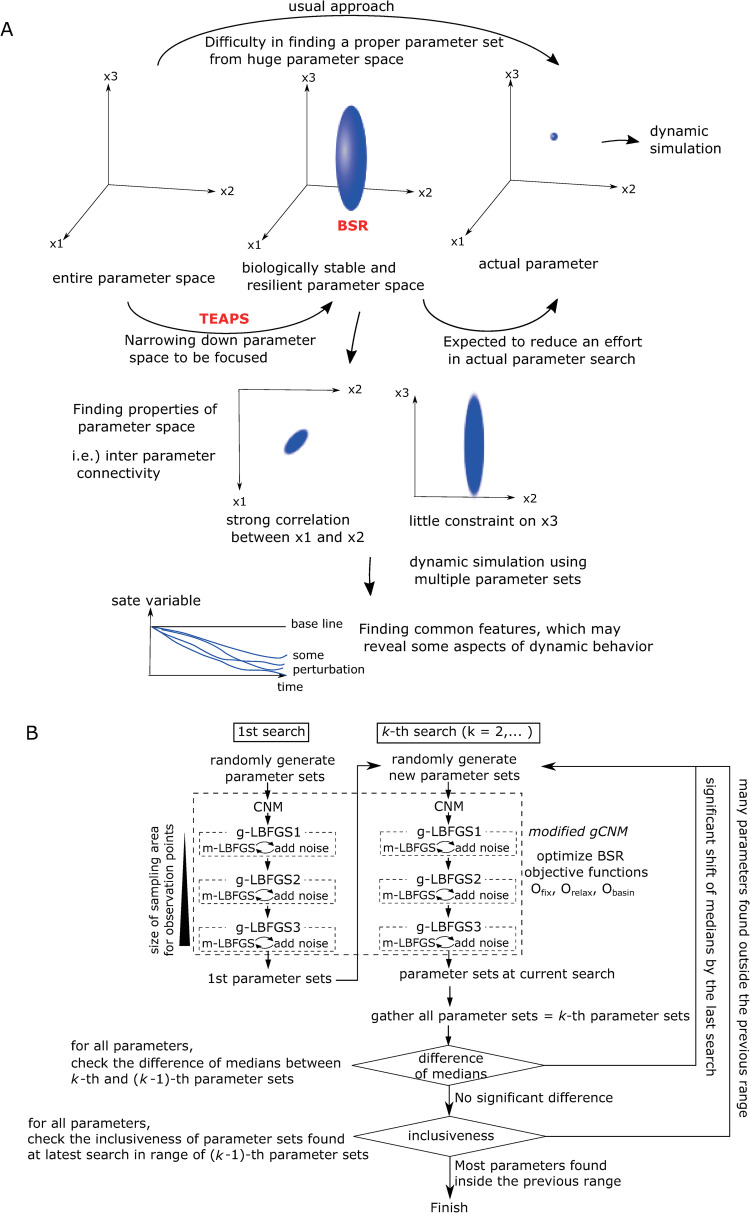
Advantages of focusing BSR and framework of TEAPS. (A) Advantages of focusing BSR were depicted. See detail in the text. (B) The framework of thorough exploration of parameter sets satisfying the BSR constraints, which we named TEAPS.

The designed objective function is helpful in finding parameter sets satisfying BSR. If we can find a parameter space satisfying BSR entirely, there are some advantages in analyses of the focused dynamic model (Fig 2A). First, the actual parameter sets for model are expected to be included in the BSR space. This feature is potentially useful in the parameter determination process especially for large scale models in which many parameters are included. For those models, the volume of parameter space to be searched is vast, while BSR constraints can reduce the volume and improve parameter search efficiency. Villaverde and Banga have shown the importance of model (network) structure constraints in effective tuning of parameters [21], which supports the fact that narrowing down the focusing parameter space by biological system constraints is helpful in the parameter determination process. The advantage of reducing allowable parameter space has been also demonstrated by Tan *et al*. who focus on the steady-state fluxes as constraints on parameters [22]. Second, even if we cannot determine a unique parameter set for the model, BSR can provide some insights for the model. The analysis of the distribution of parameter sets satisfying BSR will provide the inter-parameter connectivity, such as correlation between parameters, which will help understanding the model. In addition, performing multiple simulations using BSR satisfying parameter sets under some kinds of perturbations may reveal some common dynamic behaviors, which can be interpreted as a systems property under BSR. Considering these potential advantages, we developed an algorithm for finding as many parameter sets satisfying BSR as possible based on the global cluster newton method (gCNM) because the gCNM method is one method utilized to unveil the entire parameter space satisfying some constraints (Fig 2B). While the original gCNM uses Broyden’s method as a base algorithm for the globalization and optimization step, we opted to use L-BFGS method to reduce memory consumption to enable an application to the model, including many parameters. Considering that collecting parameter sets as many as possible is a key for the advantages shown above, we also included another modification. Each parameter set found by gCNM is added by noise to expand the distribution formed by all the found parameter sets; thereafter, it is re-subjected to L-BFGS based globalization and optimization step (referred to as g-LBFGS). The repetitive loop of the noise addition and optimization process is a large modification to the original gCNM (Figs 2B and S2). This modified gCNM is designed to iterate until satisfying two following conditions: (1) the median values of obtained parameter sets for all the parameters are not significantly shifted by the latest gCNM iteration, and (2) the majority of the parameter values found in the latest gCNM iteration are within the distribution range of the parameter set already found. Using this algorithm coupled with the BSR objective functions defined above, we expected that a large part of the parameter space will satisfy BSR and named this entire scheme the thorough exploration of allowable parameter space for biological systems (TEAPS).

### Evaluation of the algorithm performance using the simplest sample models

**Fig 3:**
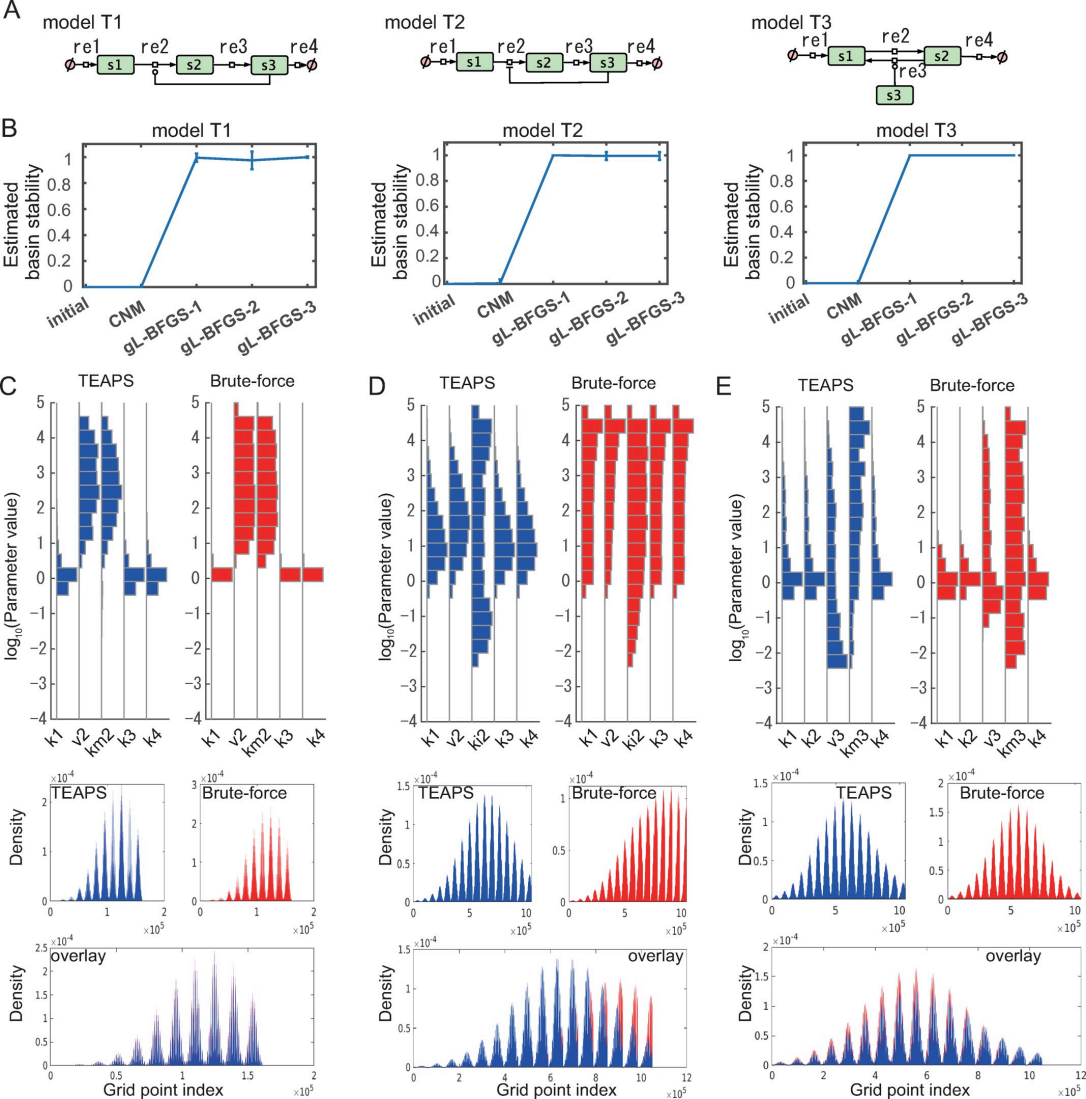
TEAPS search expands the basin stability during its process and found the parameter sets satisfying the BSR condition. (A) Three basic models were used for implementation of the BSR. (B) The estimated basin stability was increased by individual optimization process (gL-BFGS) of searching parameter sets satisfying the BSR condition using the TEAPS search algorithm. (C–E) Comparison of the parameter distributions found by the TEAPS and the brute-force searches as histograms or multidimensional density profiles for the models T1 (C), T2 (D) and T3 (E). The parameter sets satisfying the BSR condition were searched by TEAPS (upper left) or by the brute-force search (upper right), and the histograms of the distribution of the found parameter sets were compared. Density profiles of the obtained parameter sets were compared following the kernel density estimation (middle left: TEAPS, middle right: brute-force search). Two density profiles were overlaid (bottom).

Next, we evaluated the coverage of the multiple parameter sets obtained using TEAPS on the entire parameter space satisfying BSR. First, sample models (i.e., T1, T2, and T3) consisting of three biological entities that reflect the existing biological system structures were prepared (Fig 3A). T1 represents the positive feedback system and T2 represents the negative feedback system. These systems are widely observed in various biological processes, including signaling [23,24] and metabolic pathways [25]. These models do not contain reversible reactions that are fundamental to various biological processes [26]. Thus, we prepared T3 as a reversible reaction with a regulatory entity. We set the BSR conditions of these models as follows: the fixed point of the system was set to x* = (1,1,⋯,1)^T^ because the absolute value of the amount of an entity in the system depends on the unit used and important information is the relative alteration of the value. Similarly, the absolute value of time depends on the unit used. Thus, the relaxation time to the fixed point after perturbation was adjusted by setting λ*_target_ = −0.3 to observe the relaxation trend until time *t* = 100. Observation points to confirm the stable area around the fixed points were randomly generated as *x*_*m*,*n*_ = 1+*a*, where *m* is an index for observation points and *n* is an index for phase space, *a* was taken from a uniform distribution over [−*d*, *d*]. The value *d* = 0.1 was used in the final step of the algorithm because it is likely that a biological system will be stable against a perturbation that alters the amount of a certain entity by less than 10% and return to the basal point after removal of the perturbation in most cases [27,28]. To efficiently calculate in the cases of complicated models, value *d* is set to increase gradually. That is, in the CNM stage and the first subroutine of the globalization stage, the value is set to 0.004, followed by 0.036 and 0.1 in the second and the third subroutines of the globalization stage, respectively. Using these hyperparameters, we obtained multiple parameter sets for each sample model to satisfy the BSR condition. First, we evaluated whether the algorithm functions, as expected, to ensure a stable area around the fixed point. To monitor the system stability around the fixed point, the basin stability was calculated for the parameter sets acquired after each calculation step. The calculation results for each model showed that the estimated basin stability around the fixed point ***x***_*ss*_ increased as the calculation step advanced (Fig 3B). The value largely increased at the globalization stage, which was consistent with the structure of the algorithm; that is, candidate parameter sets loosely satisfying the BSR condition were collected at the CNM stage, and further optimization cycles to capture parameter sets strictly satisfying the BSR condition were carried out at the globalization stage. We then compared the parameter sets obtained using this approach with those obtained using the brute-force search (Fig 3C–3E). By brute-force search, each model showed a broad distribution for some parameters, such as *v*2 and *km*2 for T1. In contrast, there were cases of convergence to a narrow distribution of less than 0.3, such as *k*1, *k*3, and *k*4 for T1. Such profiles were reproducible using our approach for each model when the histograms of the distributions of the parameter values were compared. Because the simple comparison of the histograms cannot clearly delineate distribution shapes in multiple dimensional space, we also evaluated the density in multiple dimensional space by multivariate kernel smoothing and normalized them by the total sum. We then compared the shapes (Fig 3C–3E, middle and lower panels) based on the density. The density was calculated at grid points of the search space for parameters, and each grid point is indexed in a preset order. Thus, the densities at grid points were represented as dentsitograms. The similarity of the normalized density profiles was confirmed using the cosine similarity value and the Jensen-Shannon (JS) divergence value between the linearized vectors of each density tensor (Table 1). As a negative reference, we generated random vectors that were sampled to give the same density frequencies as the linearized vector for the brute-force search. The indices between the parameter distributions obtained by our approach and brute-force search clearly outstrip those between the random vector and brute-force search. These results indicate that the estimation of the parameter space satisfying BSR using TEAPS is practical for simple biological models.

**Table 1:** Similarities between the distributions of the obtained parameter sets which met the BSR condition by TEAPS and the brute-force search.

Model	Cosine similarity			JS divergence		
	TEAPS vs. BF	Log10 (ratio to random vs. BF) (95% CI)		TEAPS vs. BF	Log10 (Ratio to random vs. BF) (95% CI)	
T1	0.995	1.027	(1.025, 1.029)	0.0032	-2.2186	(-2.2188, -2.2184)
T2	0.890	0.785	(0.784, 0.786)	0.0262	-1.2582	(-1.2583, -1.2581)
T3	0.854	0.678	(0.677, 0.679)	0.0684	-0.7860	(-0.7861, -0.7859)
T4	0.818	1.156	(1.154, 1.157)	0.0630	-0.9621	(-0.9622, -0. 9620)
T5	0.713	0.990	(0.989, 0.991)	0.1068	-0.7180	(-0.7181, -0.7179)
T6	0.957	0.827	(0.826, 0.828)	0.0180	-1.4274	(-1.4275, -1.4273)
T7	0.975	1.108	(1.107, 1.109)	0.0191	-1.4569	(-1.4569, -1.4568)
T8	0.961	1.708	(1.705, 1.712)	0.0168	-1.5832	(-1.5833, -1.5831)

### Performance assessment of TEAPS using various simple-structured models

**Fig 4:**
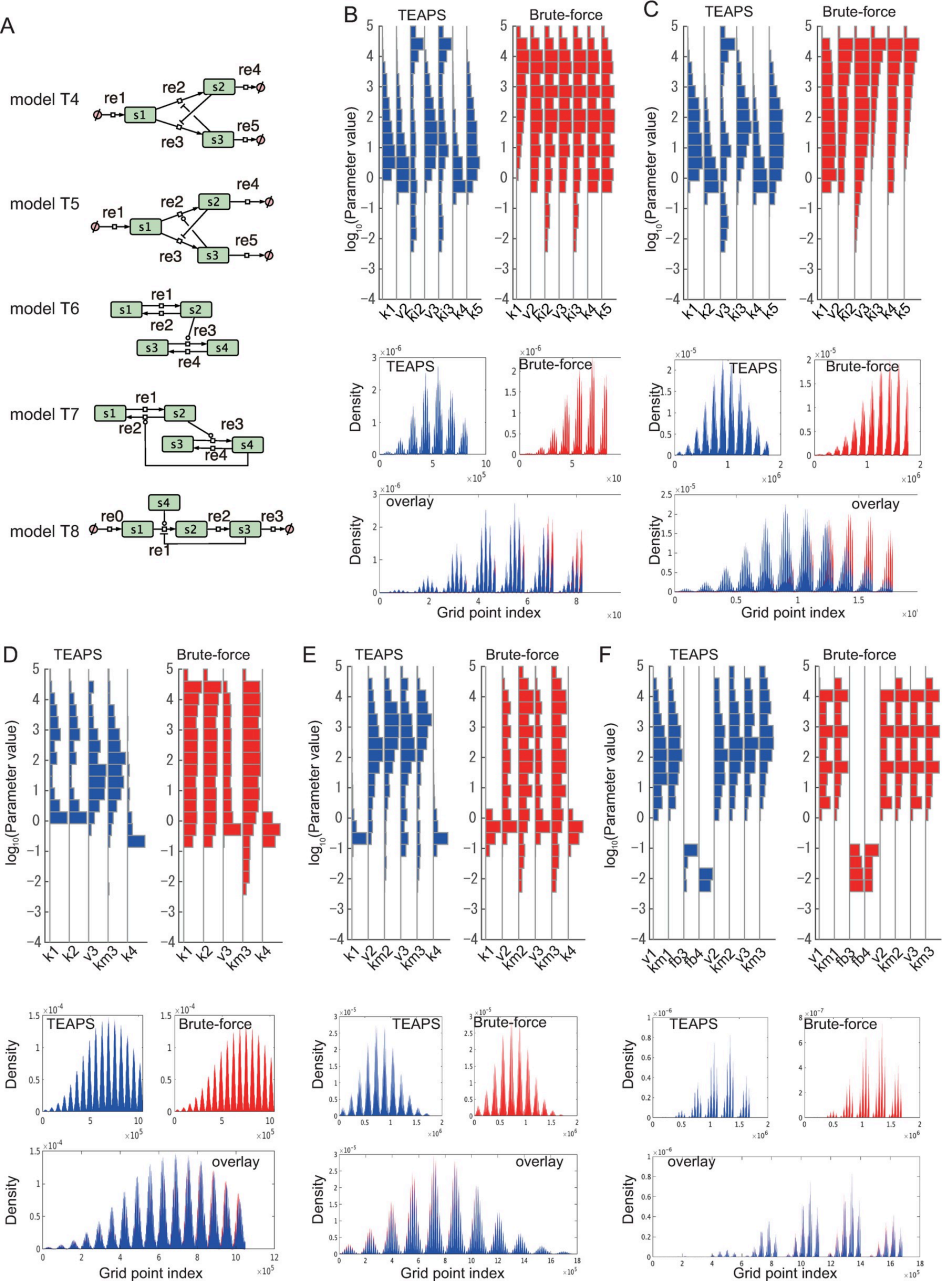
Performance assessment of TEAPS using various simple structure models. (A) Additional five sample models to be prepared for performance test. (B–F) Comparison of the TEAPS results and the brute-force search results for the models T4 (B), T5 (C), T6 (D), T7 (E) and T8 (F). The parameter sets satisfying the BSR condition were searched by TEAPS (upper left) or by the brute-force search (upper right), and the histograms of the distribution of the found parameter sets were compared. Density profiles of the found parameter sets were compared following the kernel density estimation (middle left: TEAPS, middle right: brute-force search). Two density profiles were overlaid (bottom).

Next, we evaluated the performance of TEAPS using a slightly complicated three-variable model reflecting variations in biological systems other than T1-3 (Fig 4A). Switching of the activated pathway, which is often observed in signaling pathways [29], can be simplified as expressed in T4, that is, the activation of a pathway suppresses the activation of another pathway and vice versa. T5 is a model in which the inhibitory regulation in T4 is replaced with a positive regulation. In this case, the two pathways are not mutually exclusive and cooperate to prevent the overaccumulation of end-products. The distribution of the acquired parameter sets by TEAPS aligned with those obtained by brute-force search (Fig 4B and 4C, Table 1). These results indicate great efficiency of TEAPS in searching for the allowable parameter space for various three-variable models. Subsequently, we prepared four-variable models and evaluated the performance of TEAPS. The minimum component of the signaling cascade could be modeled as expressed in T6 (Fig 4A). The algorithm could find a large part of the allowable parameter space (Fig 4D). Positive feedback regulation, which is sometimes included in signaling cascades [30], was added to T6 to build T7 (Fig 4A). Again, the algorithm found a large part of the allowable parameter space (Fig 4E). Finally, we examined T8, in which a particular reaction is regulated by multiple factors. This structure was previously reported as a representative network in biological systems [31]. This model includes nine parameters, which makes the number of grid points enormous in brute-force search; therefore, we fixed a value of *k*0 and compared the spaces for the other eight parameters. Even in the presence of complex kinetic laws by multiple regulations in a particular reaction, TEAPS successfully estimated the parameter space satisfying BSR (Fig 4F), which is intuitively understood by the combination of three-dimensional plots for three of the eight parameters (S3 Fig). Considering the possibility of biased sampling by the grid point-based search, we also performed random vector-based search to reveal the distribution of parameters satisfying BSR for all sample models. As a result, we confirmed all the three approaches, the grid point-based search, the random vector-based search and TEAPS, found the similar distribution for all models, which support the searchability of TEAPS (S4 Fig). Next, we evaluated the computation time for the entire allowable space search between TEAPS and brute-force for model T8, owing to the number of parameters being 8, which is the largest in sample models. TEAPS takes approximately 100 min for completion, while the brute-force search takes about 16 h in the same computational environment. Considering the brute-force searched for a space formed only by five parameters since the fixed-point constraints were analytically given in advance, the effective sampling by TEAPS was suggested. In addition, we performed simulations using parameter sets by TEAPS to confirm that the system returns close to the fixed point after the perturbation is added to the state variables (Computational reports in S3 Information). A series of tests confirmed the performance of TEAPS in exploring the allowable parameter space for BSR systems.

### NF-κB pathway model

**Fig 5:**
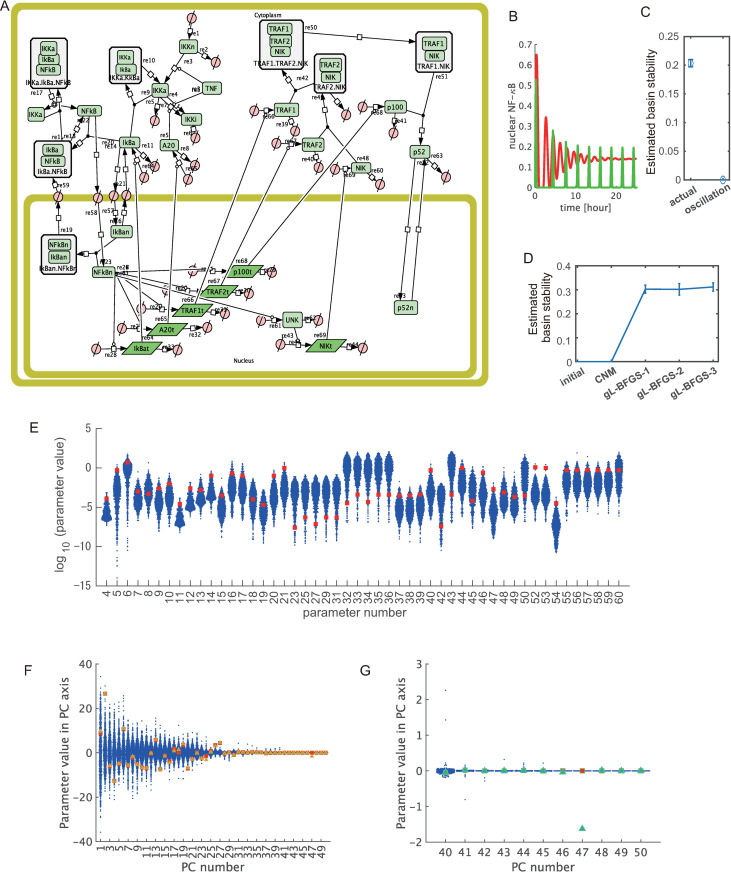
Application of TEAPS for NF-κB signaling pathway model. (A) The model structure of NF-κB signaling pathways. This signaling pathway includes feedback inhibition of NF-κB activation through IκBα and A20. (B) Time courses of NF-κB activation. The parameter set fitting to in vitro observation outputs a damped oscillation as is consistent with experimental observation (red), while the model outputs continuous oscillation when the parameter related to feedback inhibitions are changed (green). (C) The estimated basin stability for the reported parameter set (actual) and the parameter set outputting continuous oscillation (oscillation). (D) The estimated basin stability was increased by individual optimization process (gL-BFGS) when TEAPS were applied to the NF-κB model. (E) The distribution of parameter sets found by TEAPS for the NF-κB model. The found parameter values were broadly distributed (blue). For many parameters, the previously reported value (red) was included in the range found by TEAPS. (F, G) The distribution of the parameter values found by TEAPS in PC space. The reported values (red) were included in the found distribution for most axes, while the oscillative parameter set was outside of dominant distribution for later PCs. (F) The distributions for all PCs are shown. (G) The distributions of later PCs are enlarged.

Next, we examined the applicability of TEAPS in parameter space searches in a real biological network model. The model for the NF-κB signaling pathway was focused on. This system includes feedback inhibition against NF-κB in the downstream of its activation; therefore, this system may show continuous oscillatory behavior [32–34] (Fig 5A). In actual observations, this system shows damped oscillation once the signaling pathway is activated, and this behavior is consistent with the BSR constraint. To mathematically analyze this characteristic behavior, several ODE models have been developed based on various parameters, many of which were determined by fitting to actual observations [33,34] (Fig 5B, red line). We also analyzed the property of basin stability for the reported parameter set. Initial states of the system were randomly generated around the fixed point; then, the probability of convergence to around the fixed point was assessed. Hence, it was confirmed that a certain size of basin stability existed around the fixed point (Fig 5C, actual). Therefore, we examined whether TEAPS can explore the parameter space, including the reported parameter values. The target fixed point was set to the state where the model with the reported parameter set converged, and the target relaxation was set to converge within approximately 20 h to ensure consistency with actual observations [33,34]. The target size of the basin was set to be as large as that of the sample models. We confirmed the reported parameter set is consistent with this setting for BSR (Computational reports in S4 Information). By this setting, we finally obtained 1095 parameter sets by TEAPS within 2 h of computation time in our environment and confirmed that the estimated basin stability around the fixed point was also elevated in this case after the g-LBFGS (Fig 5D). For many parameters, the reported values were included in the range of parameter values found by TEAPS (Fig 5E). However, the found parameter values are broadly distributed, which makes it difficult to judge whether the critical parameter characteristics are truly determined by TEAPS. To further assess whether the reported parameter set is included in a manifold formed by the obtained parameter sets with considering the inter-parameter connectivity, primary component analysis (PCA) was performed against the obtained parameter sets and examined the inclusion in primary component axes. The number of components was set to be the same as the dimensions of the parameter space to identify the widest and narrowest directions. In all primary components (PCs), the reported value was clearly within the range of the obtained parameter space. In particular, it is worth focusing on the PC axes with narrower distributions (PC 40 and over). TEAPS picked up limited parameter sets when projected to these axes; therefore, these axes are expected to define stringent constraints for BSR. It is clearly confirmed that the reported parameter set is included in the range found in those axes with stringent constraints (Fig 5F and 5G). Next, we examined whether parameter sets giving undamped oscillatory behavior were actually outside the found parameter space. For this purpose, we searched a parameter set that changed slightly from the reported parameter set. We found that the model outputs an undamped oscillation when the values of two parameters describing the translation speed of IκBα and A20 are changed both from 0.5 to 5.0 (Fig 5B, green line). For this parameter set, we assessed the basin stability around the focused stability region, which was around the fixed point given by the original parameter set. Thus, the basin stability was confirmed to vanish when using the oscillatory parameter set (Fig 5C, oscillation). When this oscillatory parameter set was projected onto the primary component axes, this parameter set was confirmed to exist outside the dominant distribution in PC 47 (Fig 5G), which is consistent with the fact that the oscillatory behavior does not satisfy the BSR constraint. These observations suggest that TEAPS is applicable for searching parameter sets that satisfy the BSR for real biological models.

### Arachidonic acid metabolic pathway model

**Fig 6:**
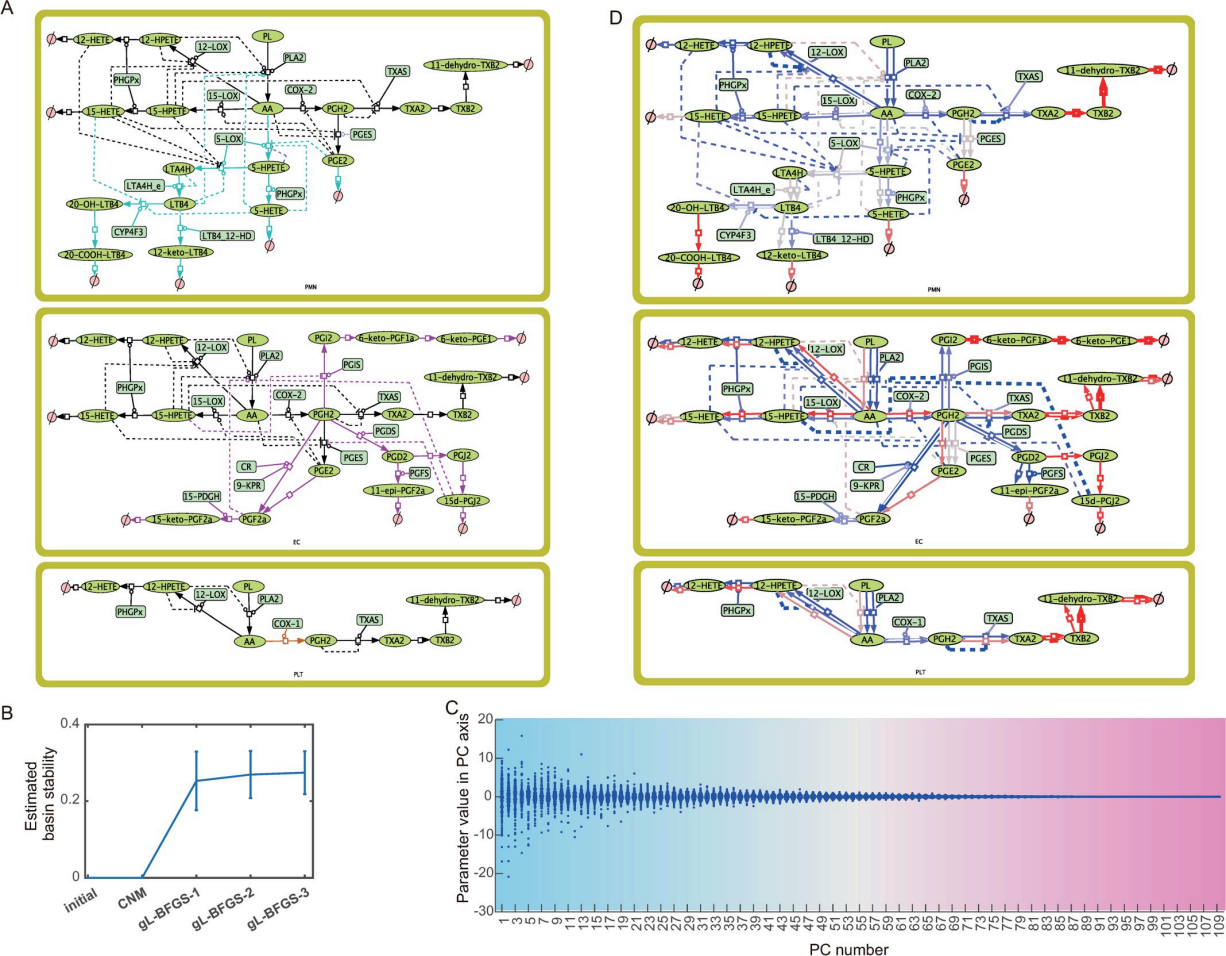
Utility of TEAPS in the analyses of arachidonic acid pathway model. (A) The model containing arachidonic acid pathways in polymorphonuclear cells (PMN), endothelial cells (EC), and platelets (PLT) was reconstructed according to the previously reported model structure. The Michaelis-Menten constants were set to the same values if there were same reactions in different cells. The reactions which only appears in each cell type are colored in cyan (PMN), pink (EC), and brown (PLT). The reactions with black arrows are shared with at least two cell types. For modifications on reactions, lines ending with a circle indicate promoting effect on a reaction whereas lines ending with a bar indicate inhibitory effect. Modificative effects by metabolite but not enzymes are described as dashed lines. (B) The estimated basin stability was increased by individual optimization process (gL-BFGS) when TEAPS were applied to the arachidonic acid pathway model. (C) The parameter sets obtained for the arachidonic acid pathway model were subjected to the principal component analysis. To figure out the entire shape of the parameter distribution, the number of components was set to the number of parameters in the model. (D) The reactions with tight and robust regulation were searched. Each arrow corresponds to each parameter on every reaction. The reactions related with parameters that appeared as high contribution to PCA axes in which the parameter distributions are narrow are colored in red, while those to each of the PCA axes in which the parameter distributions are broad are colored in blue.

We then evaluated the utility of the parameter sets found by TEAPS in a more complicated biological system. A previously reported model with which the dynamic properties of the system have been well analyzed is suitable for this purpose. Thus, we selected an arachidonic acid (AA) metabolic pathway in polymorphonuclear cells (PMNs), endothelial cells (ECs), and platelets (PLTs) [1,35]. These models were used to simulate the effects of 11 non-steroidal anti-inflammatory drugs (NSAIDs) (i.e., aspirin, ibuprofen, naproxen, 6-methoxy naphthalene acetic acid (6-MNA), acetaminophen, indomethacin, meloxicam, nimesulide, diclofenac, celecoxib, and rofecoxib) on the concentrations of prostaglandins. We combined the three separate models of AA pathways in PMNs, ECs, and PLTs into a single model consisting of 75 biological entities, 60 reactions, and 109 kinetic parameters (Fig 6A). In this model, a single parameter value was adopted for the Michaelis-Menten constant to describe the same enzymatic reaction in different cell types. Reactions including such shared kinetic parameters are colored black, and cell type-specific reactions are differently colored as in Fig 6A. In this regard, models of different cell types were linked to a single model. We applied TEAPS to this model and evaluated its searchability. The value of each variable at the fixed point was set to 1, and a 10% perturbation around the fixed point was assumed to be allowable, as in the sample model analyses. Within a realistic time period (about 3 days), we obtained 1391 parameter sets out of 4000 initial sets that converged near the target fixed point. In addition, during the process of TEAPS, basin stability was confirmed to increase (Fig 6B). These observations suggest that TEAPS can be applied to a certain scale of a real biological model, similar to this example.

Next, we analyzed the properties of the obtained parameter sets. Because one of the features of TEAPS is the collection of multiple parameter sets from the allowable parameter space, the shape of this space could include the information of model constraints to satisfy the BSR condition. Sensitivity analyses are usually performed by repeating simulations to determine the parameters or reactions that crucially affect the system behavior. Conversely, in cases where we have information about the allowable parameter space to satisfy the BSR conditions, the identification of the narrowest direction in the parameter space will possibly lead to the identification of crucial parameters affecting system behaviors. Given that a simple evaluation of the distribution of each parameter value would be inappropriate owing to inter-parameter connectivity, we adopted PCA. The number of components was set to be the same as the dimensions of the parameter space to identify the widest and narrowest directions (Fig 6C). We also calculated an index termed the principal central axis index (PCI) for each parameter (see Method section); it is a weighted average calculated by multiplying each PC axis number by the relative loading of each parameter on its axis (S1 Table). This index is designed to represent a hypothetical PC axis number between 1 and 109, where each parameter largely contributes. Therefore, the parameters with a small PCI value are assumed to contribute to the PC axes wherein the found parameter distribution is broad. In this context, we searched for parameters with small PCI values, which are expected to vary significantly even if the system fulfills the BSR requirements. These parameters are shown in blue in the model (Fig 6D). Note that a parameter, but not a reaction, is indicated by one arrow in Fig 6D. Among the blue-colored parameters, those related to AA production in each cell were distinctive. Given that AA production corresponds to the initiation process of metabolic reactions in each cell, the system can reach a steady state by balancing the fluxes of downstream reactions with AA production fluxes. Thus, it is reasonable that parameters related to AA production can take a broad range of values to satisfy the BSR constraints. In contrast, parameters having a large PCI value are major contributors to the PCA axes in which the found parameter distribution is narrow. In these PCA axes, the linear combination of several parameter values can take a narrow range. Therefore, the parameters contributing to those axes can take a value range tightly limited by other parameters contributing the same PCA axis to fulfill the BSR constraints, leading to the restriction of the parameter flexibility. In Fig 6D, these parameters are shown in red. We can easily notice that the downstream reactions are less flexible than the other reactions. These reactions are largely non-enzymatic processes whose fluxes are described by one parameter, and the flux of each reaction needs to be balanced with the flux of the subsequent reaction at the steady state. Therefore, it is quite reasonable that these reactions are concentrated in the narrowest directions, which indicates that the parameter sets acquired by TEAPS seem to successfully outline the allowable parameter space satisfying the BSR constraint even in a system consisting of more than 100 kinetic parameters.

### Applications of parameter sets obtained by TEAPS in systems biological analyses

**Fig 7:**
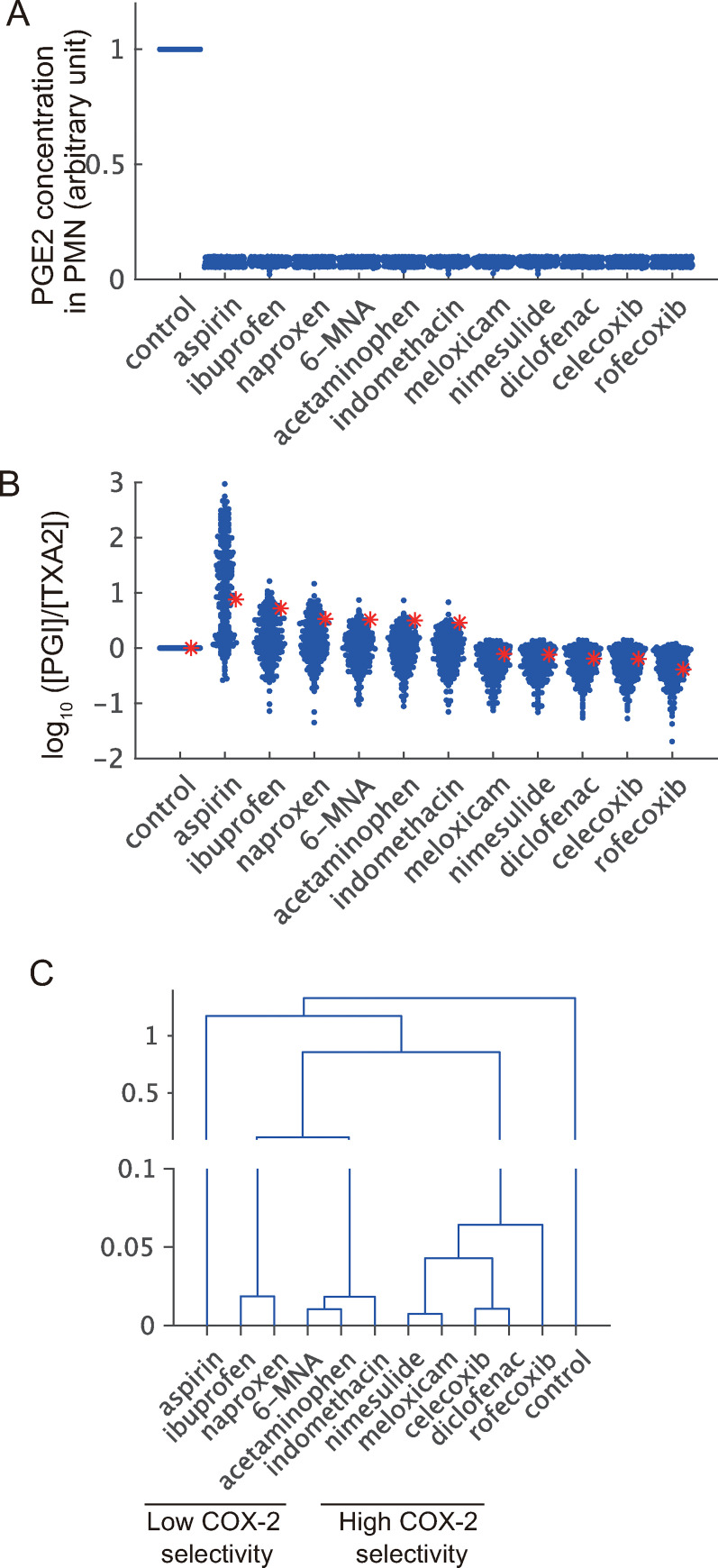
Part of parameter sets obtained by TEAPS can reproduce the *in vivo* phenotype associated with the administration of NSAIDs. (A, B) The situations where each NSAID was administered to suppress PGE2 levels in PMN to approximately 10% of control were compared with the previously reported article. Each blue point corresponds to a result by one parameter set obtained by TEAPS. Red asterisks indicate the reported values by the simulation results using the previously reported model. PGE2 level in PMN (A) and the ratio of PGI2 to TXA2 (PT ratio) (B), a marker of physiological output, are shown. (C) Patterning of pharmacological outputs by clustering analysis showed the difference of pharmacological actions. Clustering analysis was performed based on the pattern of the simulated PT ratios by the obtained parameter sets under each drug exposure. The COX-2 selectivity was figured out by the phylogenetic tree.

In previous reports, the AA model was used to compare the pharmacological effects of NSAIDs using parameters fitted to experimental observations. Therefore, we next examined whether similar pharmacological characteristics could be extracted using TEAPS-based parameter sets. In previous reports, the pharmacological actions of NSAIDs were expressed as concentration-dependent correction factors on kinetic parameters describing the activities of cyclooxygenase (COX)-1 and COX-2, and the concentrations of prostaglandins at steady state were analyzed [35]. This means that the pharmacological effects were evaluated as the alteration of the fixed point of the system associated with the changes in particular parameter values. TEAPS was designed to find the condition where the system relaxed to the same fixed point when the initial values of the state variables were at a certain distance from the fixed point; in contrast, the fixed point could possibly shift when particular kinetic parameters included in each parameter set obtained by TEAPS were changed. Therefore, although TEAPS finds parameter sets based on the stability of systems, pharmacological analyses such as those in the previous report can be performed using TEAPS-based parameter sets. As an initial step, we performed simulations using each parameter set obtained by TEAPS to identify parameter sets with which the reduction of PGE2 concentration by NSAID exposure can be reproduced, since the reduction of PGE2 levels in PMNs was used as an indicator of the pharmacological effect of NSAIDs in a previous report [35]. We examined whether the PGE2 level in PMNs can be reduced to about 10% by increasing the concentration of each drug, as has been performed in a previous report. Specifically, the initial concentration of each drug was set to be one-hundredth of the IC_50_ value of each drug against COX-2, and the drug concentration was increased in a 1.5-fold stepwise manner until the simulated PGE2 level in PMNs was reduced to less than 15% of that without NSAIDs exposure. The parameter sets with which PGE2 reduction was not achieved even when the drug concentration exceeded one hundred-fold of the IC_50_ value were excluded from the following analyses. Consequently, we determined the concentration of each drug for the selected 234 parameter sets (Fig 7A). Thereafter, the alteration in the ratio of prostacyclin concentration to thromboxane A2 concentration (PT ratio) was simulated and compared with the reported values [35], given that this index is often used as an indicator of cardiac adverse reactions [36,37]. The mean values of the calculated PT ratios across 234 parameter sets decreased in the following order: aspirin, ibuprofen, naproxen, 6-MNA, acetaminophen, indomethacin, meloxicam, nimesulide, diclofenac, celecoxib, and rofecoxib (Fig 7B). This was consistent with the observations in the original report, in which each parameter value was estimated independently by fitting the model to the experimental observations. Next, we examined whether it was possible to classify drugs based on the simulated results. If the pharmacological properties of drugs A and B are similar, it is plausible to assume that the PT ratios for drugs A and B will be close in any simulation using each of the obtained parameter sets. Thus, for each drug, vectors consisting of PT ratios for all parameter sets were constructed, and the similarity of vectors for all drugs was assessed by clustering analysis (Fig 7C). One cluster consisted of ibuprofen, naproxen, 6-MNA, and acetaminophen. These drugs share a feature of low COX-2 selectivity [35]. Another cluster consisted of high-selectivity drugs such as meloxicam, nimesulide, diclofenac, celecoxib, and rofecoxib. This classification is consistent with the known pharmacological properties of NSAIDs. These results suggest that the parameter sets obtained using TEAPS include those reflecting some pharmacologically important biological events.

## Discussion

In the present study, we attempt to obtain as much information as possible regarding the model dynamics even in cases that the parameter values are hardly determined. To analyze the dynamic behavior with avoiding parameter determination process, one approach is data-driven model construction, which is sometimes achieved by machine learning. For example, the approach by Henriques *et al*. does not require individual parameter determination [38]. This approach is quite powerful when large scale observation data over time are available. When the focus is on the intracellular events, such as signal transduction system, such timeseries data for many biological molecules are unfortunately not available in many cases. We think complementary approaches for such cases are also required to analyze the biological models. We proposed an approach with avoiding precise parameter determination and without any requirement of large-scale observation data. To achieve this, we adopted an approach to pick up all allowable parameter sets under some biological constraints.

We focused on biological systems satisfying BSR conditions in this study. The concept of dynamic stability of the biological models has been mentioned as robustness around 2004 [39]. In this literature, the author explained many biological systems consist of signal receiving components, core processing components, and output components, and that there are feedback loops from output components to signal receiving components in many cases, which is a reasonable structure for systems to maintain a certain state. In addition, the author also mentioned that too strong a robustness causes loss of a chance in state transition by any stimulation; therefore, the robustness was thought to exist in a limited range around a certain state. Our concept of BSR is well reflected the robustness, thus, TEAPS method will be applicable to most metabolic systems [1–3] and various signaling systems, such as cellular responses to external stimuli [4,5]. In order to obtain parameter sets that satisfy this stability concept, we designed objective functions using linear approximation strategies for the model such as calculating eigenvalues of Jacobian matrix around the fixed point. This approach is reasonable since the usefulness of the eigenvalues of the Jacobian matrix in the evaluation of state stability has been well recognized in the field of control theory [40,41]. In our designed objective function, we need to define the region of interest for evaluating state stability. We tentatively set the size of the stable area to allow 10% change in each variable, as per the two previous reports. One report showed that the mRNA expression level had approximately 30% variability between clones isolated from the same cell line [42]. The variability of expression level may be a result of fluctuations around a particular value, or it may indicate that there are many stable points in that range. In either case, the system was stable in that range. Another report focused on personalization of the cellular metabolism model [43]. In this report, the parameters for the erythrocyte metabolic pathway kinetic model were individually determined for 24 individuals. The difference in kinetic parameters between individuals was 14.5% as the median value of differences for all parameters. The amounts of molecules regulated by such kinetic parameters potentially vary on a similar scale if the system is linear. Despite some exceptions, such as complicated nonlinear systems, we assumed that many biological systems can tolerate at least 10% changes in each variable. In cases where this assumption is unrealistic, this value can be changed by considering prior information about the system of interest. For example, in the case of a biological event, including transitions of the state of a system to other states, such as multistep cellular differentiation [44], the stable range of each single state might be narrower than that of states in a uni-stable system and the corresponding setting should be applied in TEAPS. We also have to be careful about the possibility that the system has a number of fixed points in these cases. Conceptually, when we identify several steady-states for a certain system and try to find parameter sets satisfying BSR for all the steady-states, we can separately search parameter space satisfying BSR for every steady-state, then we can find the overlapped space, but actual performance should be assessed as further tasks. In contrast, the TEAPS method will not be applicable to biological systems with autonomous periodic behaviors, such as the electrical conduction system of the heart [45], circadian rhythm generating system [46], and regulation of cell cycles [47]. If an objective function to reflect the periodic behaviors can be designed, then the modified global CNM algorithm used in the present study might be applicable for analyses of the systems without determining the exact values of the kinetic parameters. In addition, if there are systems that satisfy BSR but are non-continuous and nondifferentiable, TEAPS cannot be applied to such systems.

We modified the global CNM to enable the sampling of multiple parameter sets from the entire solution space in a relatively unbiased manner (Figs 3 and 4). However, there are some cases with the risk of insufficient search. One such case is where the true allowable parameter space is large, similar to model T2, whereas the TEAPS search uses a finite number of parameter sets for space search. If the number of seeded parameter sets is inadequate, the parameter set satisfying the BSR constraint may be sparsely found in the entire parameter space, which makes it difficult to outline a true allowable parameter space. In addition, the globalization process may be insufficient if the true allowable space is large. Because TEAPS includes steps of noise addition during optimization to minimize the above-mentioned risk, modification of the noise addition process may further improve the searchability for some cases. Further, we have to be careful about the possibility that the allowable parameter space is not necessarily connected. The optimization algorithm used in this study expands the searchable area adjacent to the solution space found in the earlier steps of the optimization process. Thus, if a number of isolated parameter spaces are allowable for the system to satisfy the BSR conditions, there is a risk that the algorithm could lose a part of the solution space. The concept of finding additional solution using the already found solution or values in its neighboring regions as initial value was implemented to overwhelm the local minima problem in several reports [48,49]. Since our approach is similar to this concept, our current implementation may have potential to find isolated solution space. Considering that several stochastic processes are included in the TEAPS method, repetition of independent trials with different initial parameter sets might reduce the risk of losing isolated solution spaces.

To analyze the dynamic behaviors of a system, numerical simulation is often performed using a parameter set determined by fitting the model to experimental observations [5]; however, this approach has limitations. Depending on the system structure and available experimental data, it is possible that parameter sets other than the one selected can also reproduce the experimental observations. In this case, the simulation using the selected parameter set cannot possibly reproduce the system behaviors that are not reflected in the experimental data used for parameter determination. This issue has been categorized as an overfitting problem that is yet to be solved [16,50]. Parallel simulations using multiple parameter sets might be helpful in avoiding this issue, as proposed in this study. Although determining the parameter set that is closest to the real values from other parameter sets is difficult, common features or trends observed in the parallel simulation results reflect the essential properties of the system behaviors. This approach is similar to the bagging strategy often used in statistics, where the common tendency in predictions of a large number of different models (with different parameters) is extracted to prevent overfitting [51]. From a different perspective, predicting the dynamics of systems as a superposition of multiple possibilities may be reasonable. The kinetic parameter value for a particular molecule changes depending on the external environment, such as the state of molecular crowding [9]. In that regard, what we observe may be a superposition of multiple possibilities. In addition, analysis of the shape of the allowable parameter space based on the multiple parameter sets, with which the model behaviors are plausible, is helpful in understanding the features of the system behavior and structure, as shown in the models of the NF-κB signaling pathway and AA pathway (Figs 5F and 6D). Analysis of the shape will raise candidate parameters that significantly influence the behavior of the system. The exact determination of such parameters by experimental observations may increase the predictability of the dynamics.

Finally, we propose a possible utility of our approach for predicting the behavior of systems. Dynamic simulations of many biological events are often difficult because of the availability of reliable kinetic parameter values or related biological observations. Even in such cases, our method can collect many parameter sets under the constraint of the BSR. Using these sets, it may be possible to find possible alterations in the system state that can occur under some perturbations. We could speculate the effect of perturbations by analyzing the trends of the simulated alterations under the assumption that the system obeys the constraint of the BSR. For example, when we consider the situation where information on NSAID effects on PGE2 is unavailable, unlike in the case of Fig 7, we cannot use a constraint of PGE2 concentration for selecting biologically plausible parameter sets. Even in this case, simulating the NSAID effects using all acquired parameter sets may have some biological implications. By simulating all parameter sets, we first found that simulations with many parameter sets return tiny changes in the PT ratio at the clinical concentration of each drug. This is reasonable because a part of the parameter sets still satisfies the BSR condition even after parameter modifications corresponding to the perturbations are added, which may imply that the exposure of each drug at the clinical concentration is unlikely to change the PT ratio. Next, when we focused on parameter sets with which the PT ratio was simulated to alter more than a certain level (e.g., 10%) at a clinical concentration of at least one drug, the simulated results were consistent with clinical observations (S5 Fig). Under low-dose aspirin therapy, the reduction in PGE2 level was found to be weak, and the PT ratio was higher compared to that under untreated conditions, which was consistent with the clinical use of low-dose aspirin as an anti-platelet therapy [52]. In the case of rofecoxib, the median value of PT ratios was the lowest, which is consistent with the high risk of cardiac events with rofecoxib [53]. These suppositional simulation results support that our approach may be helpful in extracting the dynamic behaviors of biological systems whose kinetic parameters are unavailable.

Collectively, the assumption of the BSR conditions and acquisition of as many plausible parameter sets as possible is a new effective strategy for understanding the dynamic behaviors of a biological system.

## Materials and methods

### Model file preparation

All models were prepared as SBML compliant XML files using Cell Designer 4.4 [54].

#### Model T1

The following are the equations indicating rates of the reactions:

$$\begin{array}{l} re1=k1\;(input\;flux),\\ re2=\frac{v2∙[s3][s1]}{km2+[s1]},\\ re3=k3∙[s2],\\ re4=k4∙[s3]. \end{array}$$

The ODE system is composed as follows:

$$\begin{array}{l} \frac{d[s1]}{dt}=re1-re2,\\ \frac{d[s2]}{dt}=re2-re3,\\ \frac{d[s3]}{dt}=re3-re4. \end{array}$$

#### Model T2

The following are the equations indicating rates of the reactions:

$$\begin{array}{l} re1=k1\;(input\;flux),\\ re2=\frac{v2∙[s1]}{1+\frac{\left[s3\right]}{ki2}},\\ re3=k3∙[s2],\\ re4=k4∙[s3]. \end{array}$$

The ODE system is composed as follows:

$$\begin{array}{l} \frac{d[s1]}{dt}=re1-re2,\\ \frac{d[s2]}{dt}=re2-re1,\\ \frac{d[s3]}{dt}=re3-re4. \end{array}$$

#### Model T3

The following are the equations indicating rates of the reactions:

$$\begin{array}{l} re1=k1\;(input\;flux),\\ re2=k2∙[s1],\\ re3=\frac{v3∙[s3][s2]}{km3+[s2]},\\ re4=k4∙[s2]. \end{array}$$

The ODE system is composed as follows:

$$\begin{array}{l} \frac{d[s1]}{dt}=re1+re3-re2,\\ \frac{d[s2]}{dt}=re2-re3-re4,\\ \frac{d[s3]}{dt}=0. \end{array}$$

#### Model T4

The following are the equations indicating rates of the reactions:

$$\begin{array}{l} re1=k1\;(input\;flux),\\ re2=\frac{v2∙[s1]}{1+\frac{\left[s3\right]}{ki2}},\\ re3=\frac{v3∙[s1]}{1+\frac{\left[s2\right]}{ki3}},\\ re4=k4∙[s2],\\ re5=k5∙[s3]. \end{array}$$

The ODE system is composed as follows:

$$\begin{array}{l} \frac{d[s1]}{dt}=re1-re2-re3,\\ \frac{d[s2]}{dt}=re2-re4,\\ \frac{d[s3]}{dt}=re3-re5. \end{array}$$

#### Model T5

The following are the equations indicating rates of the reactions:

$$\begin{array}{l} re1=k1\;(input\;flux),\\ re2=k2∙[s1][s3],\\ re3=\frac{v3∙[s1]}{1+\frac{\left[s2\right]}{ki3}},\\ re4=k4∙[s2],\\ re5=k5∙[s3]. \end{array}$$

The ODE system is composed as follows:

$$\begin{array}{l} \frac{d[s1]}{dt}=re1-re2-re3,\\ \frac{d[s2]}{dt}=re2-re4,\\ \frac{d[s3]}{dt}=re3-re5. \end{array}$$

#### Model T6

The following are equations indicating rates of the reactions:

$$\begin{array}{l} re1=k1∙[s1],\\ re2=k2∙[s2],\\ re3=\frac{v3∙[s2][s3]}{km3+[s3]},\\ re4=k4∙[s4]. \end{array}$$

The ODE system is composed as follows:

$$\begin{array}{l} \frac{d[s1]}{dt}=re2-re1,\\ \frac{d[s2]}{dt}=re1-re2,\\ \frac{d[s3]}{dt}=re4-re3,\\ \frac{d[s4]}{dt}=re3-re4. \end{array}$$

#### Model T7

The following are the equations indicating rates of the reactions:

$$\begin{array}{l} re1=k1∙[s1],\\ re2=\frac{v2∙[s4][s2]}{km2+[s2]},\\ re3=\frac{v3∙[s2][s3]}{km3+[s3]},\\ re4=k4∙[s4]. \end{array}$$

The ODE system is composed as follows:

$$\begin{array}{l} \frac{d[s1]}{dt}=re2-re1,\\ \frac{d[s2]}{dt}=re1-re2,\\ \frac{d[s3]}{dt}=re4-re3,\\ \frac{d[s4]}{dt}=re3-re4. \end{array}$$

#### Model T8

The following are the equations indicating rates of the reactions:

$$\begin{array}{l} re0=0.5,\\ re1=\frac{v1∙[s1]}{km1+[s1]}∙\frac{1}{1+fb3∙[s3]-fb4∙[s4]},\\ re2=\frac{v2∙[s2]}{km2+[s2]},\\ re3=\frac{v3∙[s3]}{km3+[s3]}. \end{array}$$

The ODE system is composed as follows:

$$\begin{array}{l} \frac{d[s1]}{dt}=re0-re1,\\ \frac{d[s2]}{dt}=re1-re2,\\ \frac{d[s3]}{dt}=re2-re3,\\ \frac{d[s4]}{dt}=0. \end{array}$$

### NF-κB pathway model

The structure of the model refers to the one from an earlier reports [33,34]. The SBML model file is also provided (S1 File).

### Arachidonic acid pathway model

The structure of the model refers to the one from an earlier report [35]. Based on the molecular interaction structure illustrated in the original report, we modified and integrated three types of cells into one model to take the same Michaelis-Menten constants for the same reactions in different cells. The SBML model file is also provided (S1 File).

### Algorithm of TEAPS

A schematic is shown in S1 Fig and detailed algorithm in S3 Information. We designed the algorithm for a thorough search of parameter sets satisfying the BSR constraints when given a search range for each parameter. We optimized the BSR objective functions using global cluster Newton method (gCNM) [17] with several modifications. Global CNM consists of two stages, CNM stage and globalized quasi-Newton method stage. In the CNM stage, rather than performing multiple iterations until strict convergence, we modified to collect the parameter sets that satisfied moderate conditions at each iteration and iterated until the number of collected parameter sets reached the preset number. In the globalized quasi-Newton method stage, Broyden’s method with modification for global search is originally implemented. In this study, we adopted limited-memory BFGS (LBFGS) based optimization to reduce memory consumption during optimization considering the situation of application to models with many parameters. We also modified the globalization method. In the original method, the direction of shift at each iteration step in the quasi-Newton method was twisted for enabling global search. We describe LBFGS with this modification as m-LBFGS. In addition to m-LBFGS, we added the step noise addition to obtained parameter sets and re-optimization step loop (g-LBFGS). We repeated g-LBFGS by expanding the size of a region for sampling observation points to assess O_basin_. This modified gCNM, consists of CNM stage and g-LBFGS stage, was repeated using different initial parameter sets until the distribution of parameter sets was not significantly shifted by inclusion of parameter sets found by the last gCNM iteration. To judge the significance, we implemented two conditions. One is Wilcoxon rank sum test between a distribution of all values for each parameter until the previous iteration and one obtained by including the last iteration. In the current implementation, the level of significance (alpha value) was 0.1. The other is that for all parameters, the majority of parameter values found in the latest gCNM iteration (specifically, more than 99% in the current setting) are within the distribution of the parameter set already found. To obtain parameter sets effectively, tuning wights for objective function is required. We found the equivalent treatment of (*O*_fix_)^2^, (*O*_basin_)^*w*^, and (*O*_relax_)^*w*^ where the weight *w* = 1~2 is applicable in many cases as a rule of thumb.

### Setting of TEAPS used in this study

The parameter search space was arbitrarily determined. For models T1 to T8, the range of 5 × 10^−3^ to 5 × 10^4^ was used except for an input flux. The range of input flux was set to 5 × 10^−1^ to 5 × 10^4^. For the arachidonic acid pathway model, the number of sequential reactions in the model, which is a kind of depth of network structure, is more than models T1 to T8, therefore, the larger relative difference of fluxes was allowed in the arachidonic acid pathway and the range of 1 × 10^−3^ to 1 × 10^24^ was used. In the objective functions, the following function and hyper parameters were used. For the fixed point restriction, *O*_fix_(***μ***) = ‖***f***(x*, ***μ***)‖_2_, we used target fixed point x* = (1,⋯,1)^T^ as mentioned in the main text. In the calculation of $O_{basin}(\mu )=ReLU\left(\underset{x\in X^{\left(k\right)}}{max}\lambda \left(M(x)\right)\right)$, 20 points were generated for ***X***^(*k*)^. The distribution of ***X***^(*k*)^ was expanded as TEAPS progress as described in 7) in the algorithm of TEAPS. In the calculation of Orelax(μ)=ReLU(maxRe(λ*)≠0Re(λ*)−λ*target),|Re(λ*)|>10−8 was used instead of Re(λ*) ≠ 0 as tolerable computation errors. Finally, the parameter satisfying ‖***f***(x*, ***μ***)‖_2_<10^−6^ and maxRe(λ*)≠0Re(λ*)<0 was judged as parameter sets converging to x* and used in further analysis.

### Estimation of basin stability

We randomly sampled 500 parameter sets in each TEAPS step. For each parameter sets, ODE integration was performed from 500 initial states around the target fixed point until time *t* = 100. For each initial state, using the state at *t* = 100 as an initial value, a state minimizing the distance between $\frac{dx}{dt}$ and (0,…,0)^T^ was searched using “fsolve” function in Matlab and was treated as a fixed point. In the current study, initial states were generated for ***x***_*i*_ including *x*_*ij*_ = 1+*a*, where *a* is taken from a uniform distribution over [−0.15, 0.15] for small models. For the NF-κB model, considering an increase of state variable dimension, initial states were generated in less small region, such that *a* is taken from a uniform distribution over [−0.105, 0.105]. For the AA model, initial states were generated in less small region, such that *a* is taken from a uniform distribution over [−0.101, 0.101]. If all components of an obtained fixed point are in ranges between 90% and 110% of the target values, the combination of the initial state and the parameter set was judged as stable. For each parameter set, a ratio of stable initial states was calculated. The means and standard errors of the ratios of all sampled parameter sets were calculated at each TEAPS step.

### Brute-force search

Grid points for the brute-force search were generated from a uniform distribution ranging from 5 × 10^−4^ to 5 × 10^4^ in a logarithmic scale. To reduce the number of points, the constraints to give the fixed point ***x***_***ss***_ = **1** were given, and the number of free parameters was reduced. The number of generated grid points were 3 × 10^8^ to 3 × 10^9^ points. For each generated grid point, the values of *O*_basin_(*μ*) and *O*_relax_(*μ*) were calculated to judge whether the point satisfies the BSR condition. For the calculation of model T8, to reduce the number of calculation points and to avoid global sparsity, only the input flux was fixed to 0.5, which was the most frequently observed value in TEAPS results. In addition to grid point-search, we also compared the searchability using random vectors sampled from the parameter space under the restriction that vectors should satisfy *O*_fix_(*μ*) = 0.

### Calculation of Jacobian matrix and its Eigenvalues

The descriptions of ODEs in SBML models were extracted. Using the extracted equations, Jacobian matrices were generated using the function “jacobian” in Maxima. Then the output was transformed into the MATLAB function format to be able to calculate the Jacobian matrix for each model in an analytical manner. Eigenvalues of the Jacobian matrix were calculated using the “eig” function in MATLAB.

### Evaluation of distribution similarity

Kernel density estimation was performed using the function “mvksdensity” in MATLAB. The bandwidth of the kernel function was calculated by the Silverman’s rule [55]. Tensors composing the estimated density at grid points in a multidimensional parameter space were vectorized. The similarity between vectors by TEAPS and brute-force search was evaluated in the following two indicators. To evaluate the similarity of the two distributions of parameter sets, the cosine similarity and the JS divergence were computed. The cosine similarity was calculated as follows: $\frac{\sum_{i=1}^{n}A_{i}B_{i}}{\sqrt{\sum_{i=1}^{n}A_{i}^{2}}\sqrt{\sum_{i=1}^{n}B_{i}^{2}}}$, where *A*_*i*_ and *B*_*i*_ are the components of vectors to be compared. This value ranges between 0 and 1 and takes a value of 1 when completely same vectors are compared. The JS divergence was calculated as follows: $\frac{1}{2}(\sum_{i=1}^{n}A_{i}\log\frac{A_{i}}{\left(A_{i}+B_{i}\right)/2}+\sum_{i=1}^{n}B_{i}\log\frac{B_{i}}{\left(A_{i}+B_{i}\right)/2})$, where *A*_*i*_ and *B*_*i*_ are the components of vectors to be compared. This value could be 0 or more and takes a value of 0 when completely same vectors are compared. To assess the superiority of TEAPS sampling to random sampling, the comparator vectors were prepared as follows: (1) density histograms of brute-force density vector were prepared for each model, (2) each component of comparator vectors was randomly sampled using the density histogram of each model as a probability distribution. By repeating random sampling, 95% confidence interval of the ratio of each similarity index in a comparison between brute-force and TEAPS to that in a comparison between brute-force and random sampling for each model is calculated.

### Clustering analyses

Hierarchical clustering of simulated PT ratios was performed. A vector consisting of a series of simulated results using multiple parameter sets for each drug was prepared. Correlation coefficients between vectors of all drug pairs were calculated. Clustering analysis based on correlation coefficients was performed by Ward’s minimum variance cluster analysis using “linkage” functions in MATLAB.

### Calculation of principal central axis indices (PCIs)

PCIs were calculated as follows: $PCI(i)=\frac{\sum_{k=1}^{n_{PCA}}\left(c_{k,i}∙k\right)}{\sum_{k=1}^{n_{PCA}}\left(c_{k,i}\right)},c_{k,i}=\frac{\left|l_{k,i}\right|}{\sum_{m=1}^{n_{PCA}}\left|l_{k,m}\right|}$, where *l*_*k*,*m*_ is the loading of the *m*th parameter on the *k*th principal component, and *n*_PCA_ is the dimension of the parameter space.

### Optimization

The base code of CNM was transferred from the code used in the previous article [56]. Solving optimization problems was performed using the L-BFGS method whose code is available as “minFunc” from the web [57]. The code was modified to expand searchable space as described in the step 3 in “Algorithm of TEAPS”. To calculate the fixed point with the obtained parameter sets, the state when the time derivatives of the amount of biological entities are zero was sought by the “fsolve” function in MATLAB.

### ODE integration

ODEs were solved by using the SUNDIALS CVode solver implemented in MATLAB or the MATLAB stiff ODE solvers.

### Computation platform

Symbolic computations were performed with Maxima. All the numerical calculations were performed with MATLAB using a Mac Pro (CPU: Xeon E5-2697 v2 2.7GHz × 1, OS: mac OS Catlina, RAM: 64GB, MATLAB version: R2019a), or a cloud computing source (AMAZON EC2, instance type: c5.12xlarge, the number of virtual CPU: 48, RAM: 96 GB, OS: AlmaLinux 8.5 64 bit, MATLAB version: R2019a, R20221b, or R2022a).

## Supporting information

S1 Fig Schematic diagram of the TEAPS algorithm. TEAPS is an algorithm to search global parameter space satisfying BSR by iterating modified global cluster Newton method (CNM). Our modified global CNM composed of CNM step and global-LBFGS (g-LBFGS) step. The g-LBFGS method composed of L-BFGS method with globalized search modification (m-LBFGS) and noise addition steps. To efficiently optimize BSR objective functions in g-LBFGS step, the objective function is changed in each subprocess as shown.
(TIF)

S2 Fig Modified global CNM method used in our study. (A) (a-c) The CNM was used for sampling the parameter sets near the target situations. Randomly generated initial parameter sets (red circles) were used as initial parameter sets for CNM (a). During the iteration of CNM, some parameter sets get close to the optima, and such parameter sets (green circles with red outline) were collected (b, c). (B) (d-g) The sampled parameter sets were used as initial parameter sets for the expansion and optimization stages. The sampled parameter sets were applied to the modified quasi-Newton method and new parameter sets were obtained (orange circles) (d). To expand the distribution, noises were added to the new parameter sets (blue circles with black outline) (e). These parameter sets were further applied to the modified quasi-Newton method and the renewed parameter sets were obtained (orange circles with black outline) (f). To expand the distribution of the parameter sets, the subroutines consisting of noise addition and optimization were repeated several times (g). Finally, the normal quasi-Newton method was used to optimize the nearest solution point from each parameter set. Thus, the distribution of parameter sets was expanded globally.
(TIF)

S3 Fig Visualization of the structure of parameter sets that met the BSR condition for model T8. Although the kinetic laws for model T8 are complicated due to multiple regulations on one reaction, TEAPS has succeeded in searching the entire shape. Each parameter set is indicated as a circle. Blue circles indicate the parameter sets found by TEAPS and red circles indicate the parameter sets found by the brute-force search.
(TIF)

S4 Fig Comparison of distribution between BSR parameter space found by grid point based or random-vector based search and one by TEAPS. The histograms of Grid point and TEAPS are same data with the main text. The random-vector based search is added in this figure. When we performed a search using random vectors, each element of which was generated by uniform distribution in the search space, and similar results were obtained for all models. In the generation of random vectors, some parameters were fixed since the fixed-point constraint for each model was analytical given in advance. The number of generated vectors were 3 × 10^8^ to 3 × 10^9^ points. For all sample models, we confirmed all the three histograms showed similar distributions.
(TIF)

S5 Fig The parameter sets obtained by TEAPS help predicting the *in vivo* phenotype associated with the administration of NSAIDs. (A, B) As a suppositional in silico experiment, we assume the situation where the effects of NSAIDs administration on PGE2 concentrations were completely unknown. This assumption was set to mimic biological models with limited or no information on quantitative behavior of systems. The situations where each NSAID was administered at the average concentration of normal clinical usage were simulated. Then, to pick up significant alterations by NSAIDs as many as possible, we focused parameter sets with which the PT ratio simulated to alter more than 10% at least one drug. Each blue point corresponds to a result by one parameter set obtained by TEAPS. PGE2 level in PMN (A) and the PT ratio (B), a marker of physiological output, are shown. Even in simulations with limited information, the trend of the PT ratio is comparable to the clinical observations, which supports that TEAPS help predicting behavior of biological systems. Green boxes indicate the median values.
(TIF)

S1 Table Principal central axis indices (PCIs) for parameters in the arachidonic acid pathway models. (PDF)

S1 Model The arachidonic acid metabolite pathway model. SBML file are provided as S1 File.
(PDF)

S1 File SBML model files. (ZIP)

S2 File TEAPS source codes A zip file containing MATLAB codes for TEAPS. The codes are also available at the following URL: https://github.com/kyoshiaki-tky/teaps_project.
(ZIP)

S1 Information Mathematical background of objective function for basin stability. The theorem and its proof were descripted.
(PDF)

S2 Information Modification of the global CNM to intensify expansion of searchable areas. (PDF)

S3 Information Entire algorithm of TEAPS. (PDF)

S4 Information Computational report. (PDF)
