# Supplementary figures and images for: Utility of constraints reflecting system stability on analyses for biological models
Source: PLoS Comput Biol. 2022 Sep 9;18(9):e1010441. doi: 10.1371/journal.pcbi.1010441 (PMC9491612; doi:10.1371/journal.pcbi.1010441)

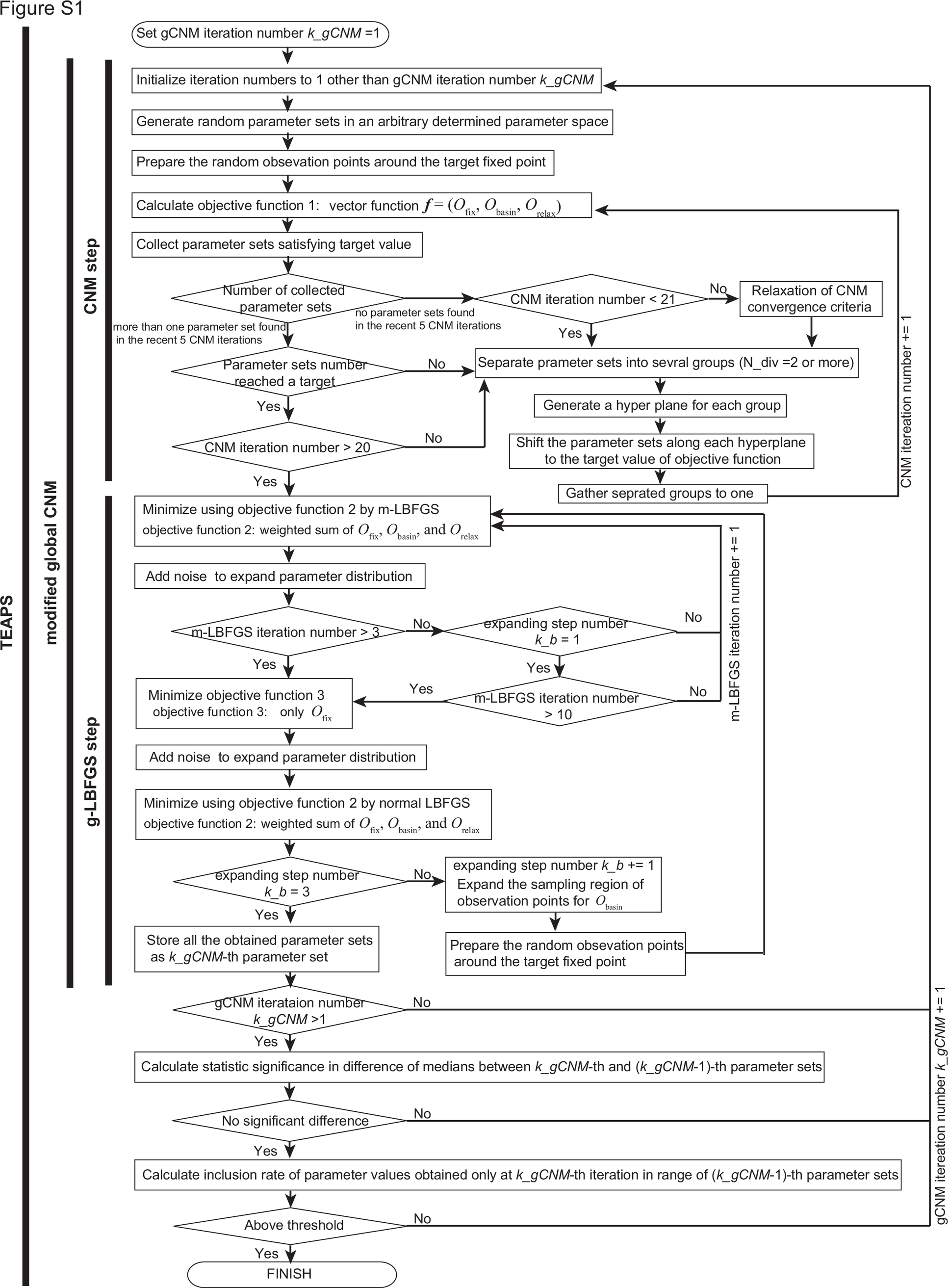

Supplement: S1 Fig — TEAPS is an algorithm to search global parameter space satisfying BSR by iterating modified global cluster Newton method (CNM). Our modified global CNM composed of CNM step and global-LBFGS (g-LBFGS) step. The g-LBFGS method composed of L-BFGS method with globalized search modification (m-LBFGS) and noise addition steps. To efficiently optimize BSR objective functions in g-LBFGS step, the objective function is changed in each subprocess as shown. (TIF) [file pcbi.1010441.s001.tif]

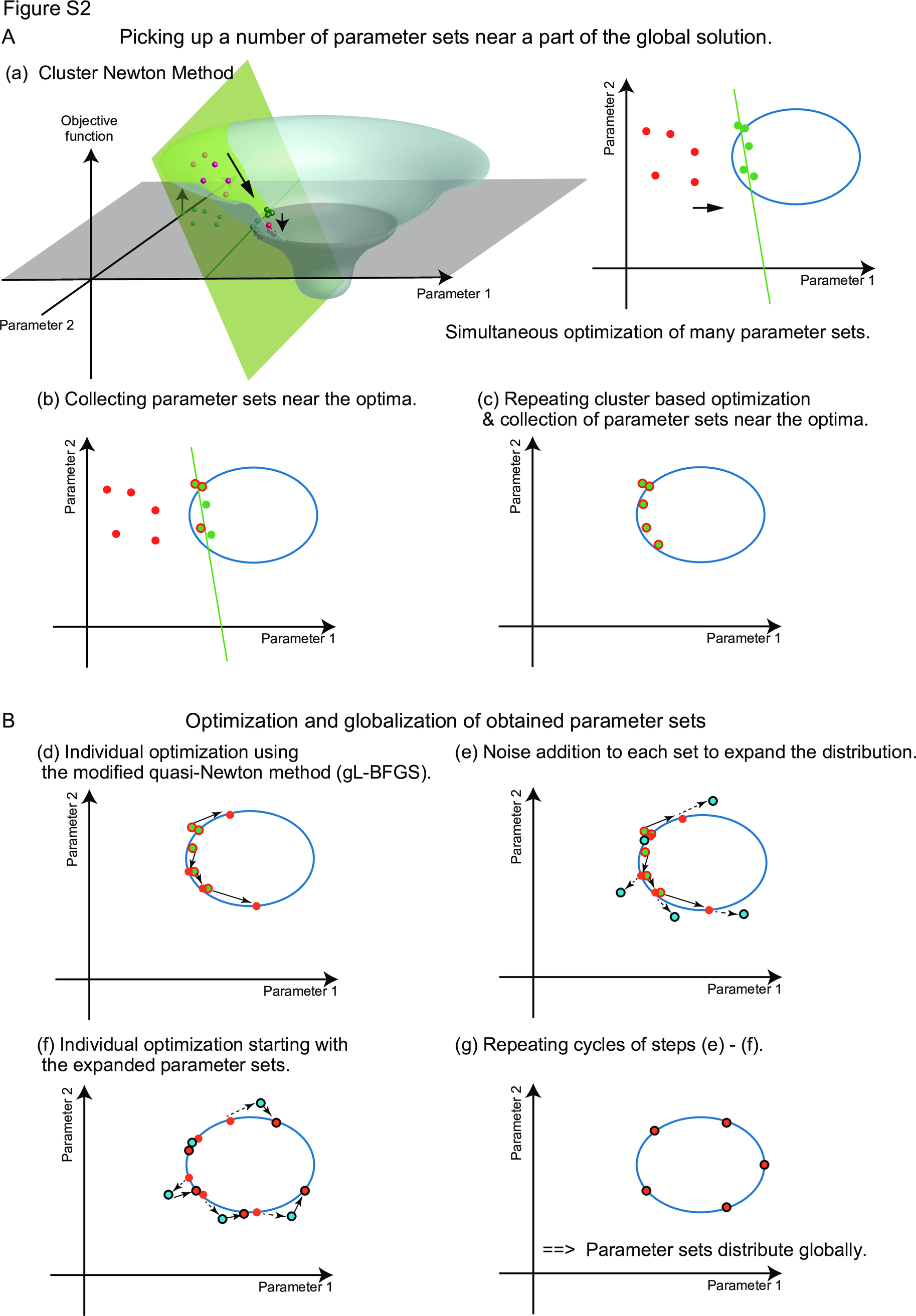

Supplement: S2 Fig — (A) (a-c) The CNM was used for sampling the parameter sets near the target situations. Randomly generated initial parameter sets (red circles) were used as initial parameter sets for CNM (a). During the iteration of CNM, some parameter sets get close to the optima, and such parameter sets (green circles with red outline) were collected (b, c). (B) (d-g) The sampled parameter sets were used as initial parameter sets for the expansion and optimization stages. The sampled parameter sets were applied to the modified quasi-Newton method and new parameter sets were obtained (orange circles) (d). To expand the distribution, noises were added to the new parameter sets (blue circles with black outline) (e). These parameter sets were further applied to the modified quasi-Newton method and the renewed parameter sets were obtained (orange circles with black outline) (f). To expand the distribution of the parameter sets, the subroutines consisting of noise addition and optimization were repeated several times (g). Finally, the normal quasi-Newton method was used to optimize the nearest solution point from each parameter set. Thus, the distribution of parameter sets was expanded globally. (TIF) [file pcbi.1010441.s002.tif]

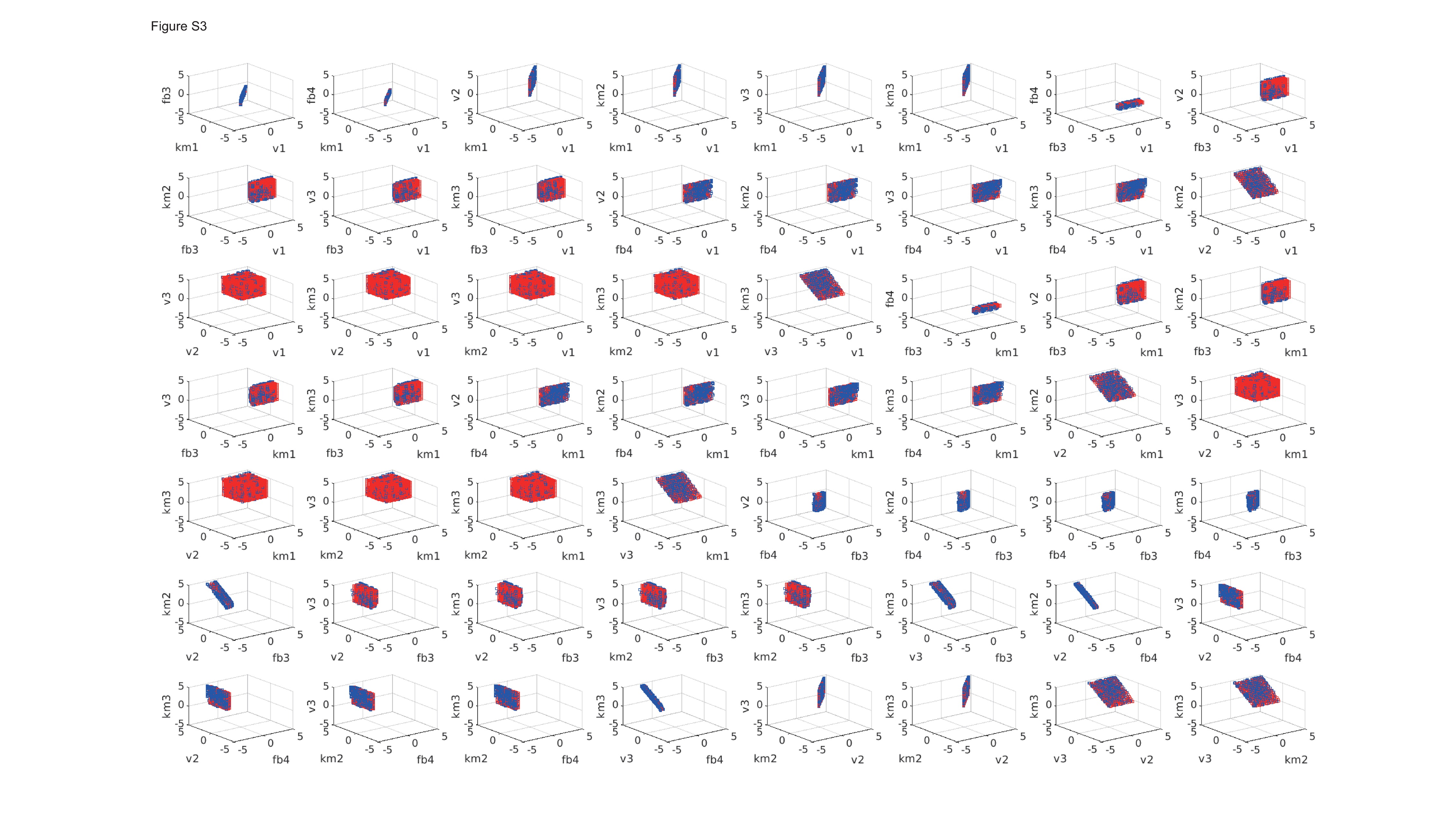

Supplement: S3 Fig — Although the kinetic laws for model T8 are complicated due to multiple regulations on one reaction, TEAPS has succeeded in searching the entire shape. Each parameter set is indicated as a circle. Blue circles indicate the parameter sets found by TEAPS and red circles indicate the parameter sets found by the brute-force search. (TIF) [file pcbi.1010441.s003.tif]

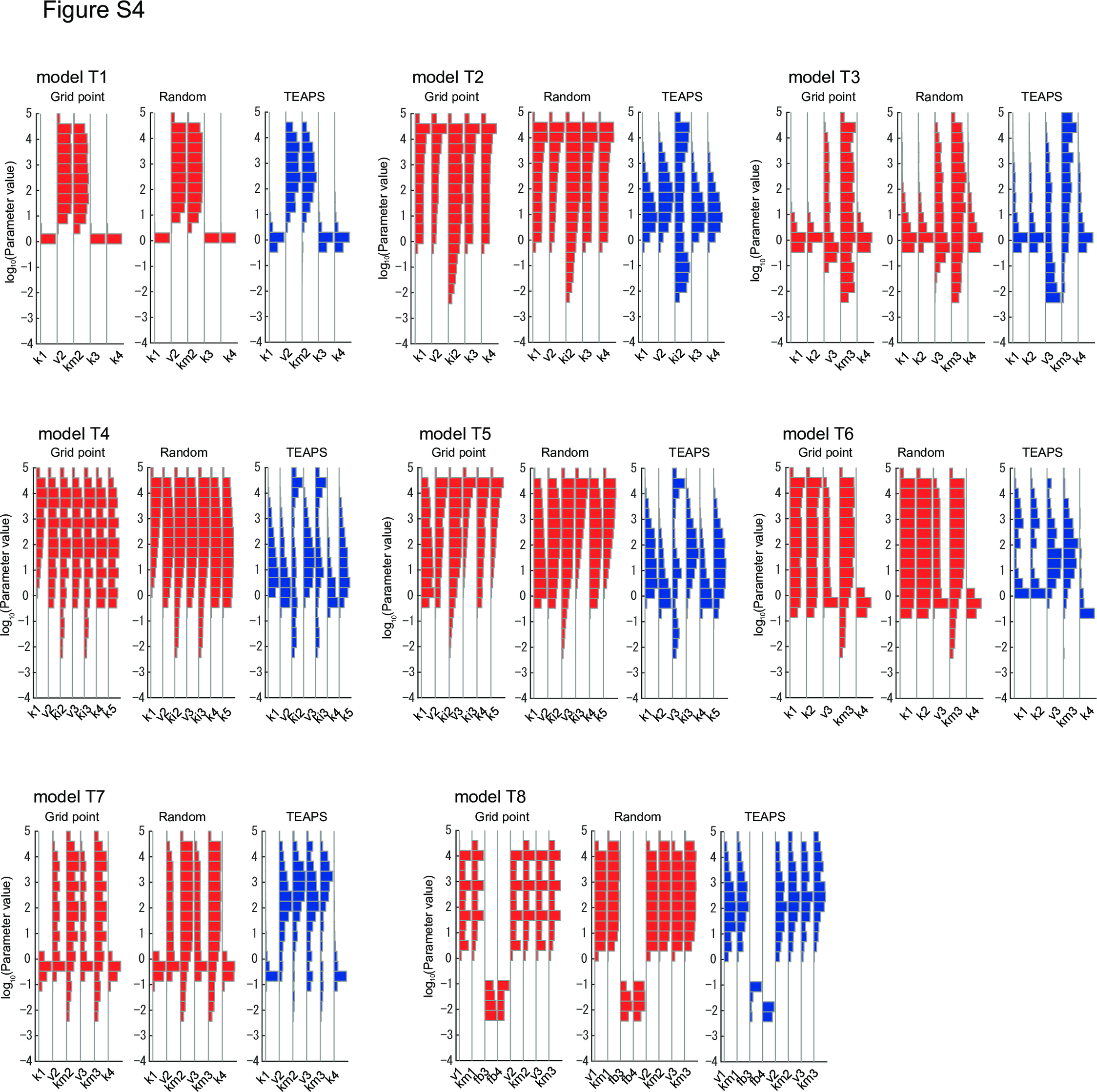

Supplement: S4 Fig — The histograms of Grid point and TEAPS are same data with the main text. The random-vector based search is added in this figure. When we performed a search using random vectors, each element of which was generated by uniform distribution in the search space, and similar results were obtained for all models. In the generation of random vectors, some parameters were fixed since the fixed-point constraint for each model was analytical given in advance. The number of generated vectors were 3 × 10^8^ to 3 × 10^9^ points. For all sample models, we confirmed all the three histograms showed similar distributions. (TIF) [file pcbi.1010441.s004.tif]

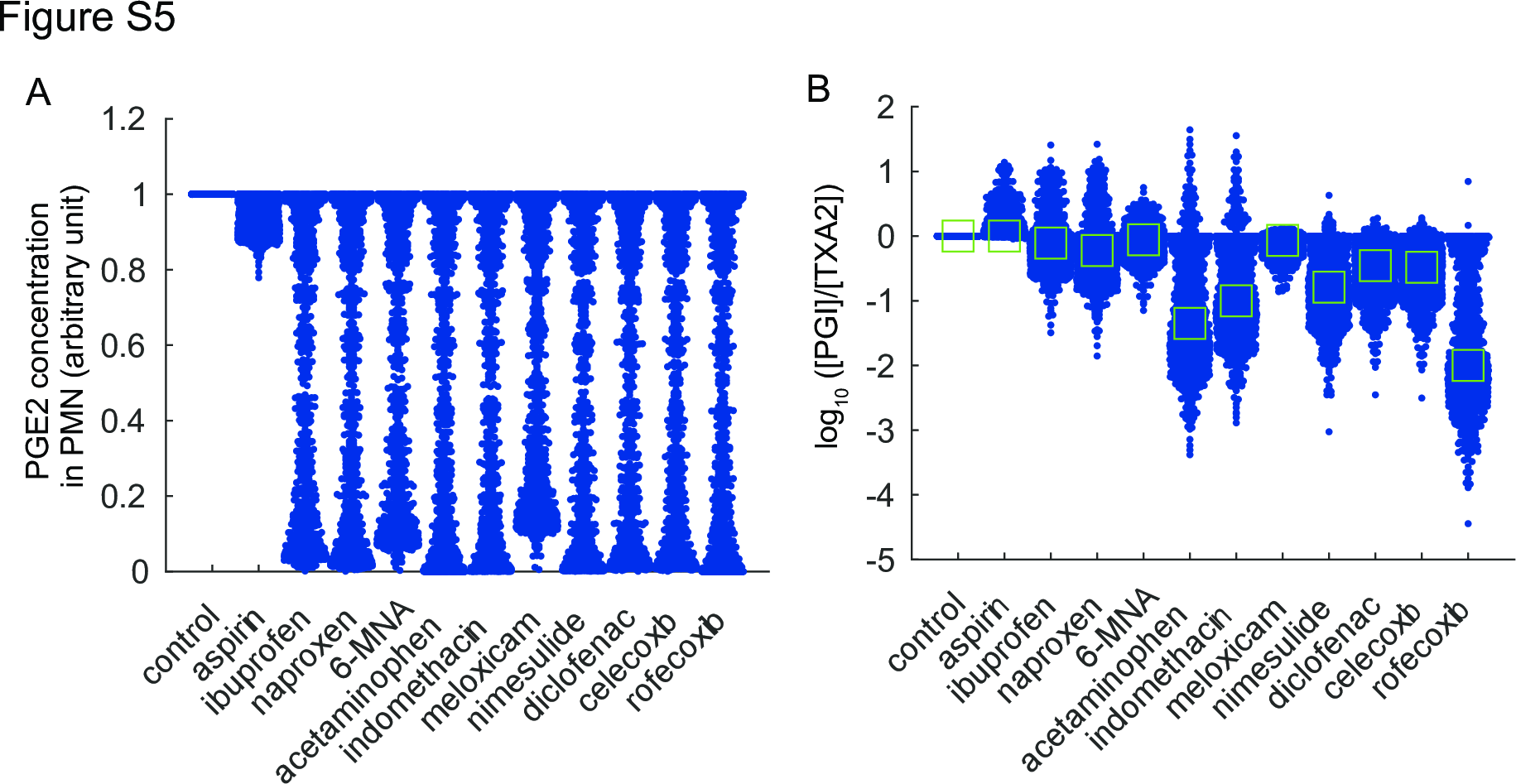

Supplement: S5 Fig — (A, B) As a suppositional in silico experiment, we assume the situation where the effects of NSAIDs administration on PGE2 concentrations were completely unknown. This assumption was set to mimic biological models with limited or no information on quantitative behavior of systems. The situations where each NSAID was administered at the average concentration of normal clinical usage were simulated. Then, to pick up significant alterations by NSAIDs as many as possible, we focused parameter sets with which the PT ratio simulated to alter more than 10% at least one drug. Each blue point corresponds to a result by one parameter set obtained by TEAPS. PGE2 level in PMN (A) and the PT ratio (B), a marker of physiological output, are shown. Even in simulations with limited information, the trend of the PT ratio is comparable to the clinical observations, which supports that TEAPS help predicting behavior of biological systems. Green boxes indicate the median values. (TIF) [file pcbi.1010441.s005.tif]
