# Supplementary material for: Utility of constraints reflecting system stability on analyses for biological models
Source: PLoS Comput Biol. 2022 Sep 9;18(9):e1010441. doi: 10.1371/journal.pcbi.1010441 (PMC9491612; doi:10.1371/journal.pcbi.1010441)
Supplement: S1 Table — (PDF) [file pcbi.1010441.s006.pdf]

Supplemental Table

S1 Table. Principal central axis indices (PCIs) for parameters in the arachidonic acid pathway models.

Parameter	PCI
Vm5	31.188
Km5	31.490
k5_5HETE	55.441
k5_LTB4	54.970
k5_15HPETE	57.855
k5_12HPETE	58.232
k23_PGF2a	58.155
k23i_15dPGJ2	11.515
Vm1	30.612
Km1	37.433
k1i_15HETE	28.026
k1i_15HPETE	41.329
k1i_12HPETE	15.392
Vm3	29.900
Km3	31.573
k55	35.178
Vm2	40.580
Km2	41.217
k2_PGE2	55.313
Vm4	38.582
Km4	38.685
k56	58.375
Vm6	46.431
Km6	50.582
k6i_5HETE	34.944
k6i_PGE2	42.446
k6i_5HPETE	38.528
Vm9	53.227
Km9	48.290
k59	72.600
Vm14	35.834
Km14	46.380
k14i_PGE2	21.085
Vm16	34.758
Km16	45.305
k16i_PGH2	18.214
k16i_15HPETE	31.675

Parameter	PCI
k17	102.192
k18	101.382
k60	101.256
Vm15	55.893
Km15	55.506
k15i_AA	28.020
k15i_15HETE	34.837
k32i_15dPGJ2	35.414
k69	73.565
Vm8	52.107
Km8	57.011
k8i_15HPETE	36.812
k8i_15HETE	31.582
k8i_12HETE	34.510
k8_LTB4	51.346
k21adj	75.643
k8i_LTA4H	35.675
Vm10	54.205
Km10	55.376
k10i_LTA4H	36.954
Vm11	53.863
Km11	43.983
Vm12	47.908
Km12	50.586
k12i_15HETE	41.648
k12i_5HETE	28.131
k13	89.570
k19adj	77.532
k20adj	85.972
k22adj	84.306
k23adj	64.708
k31adj	81.164
k32adj	69.817
k33adj	69.399
k34adj	80.275
k35adj	82.403

Parameter	PCI
Vm36	38.012
Km36	41.024
k36i_15HPETE	9.103
k37	94.619
k38	93.684
Vm39	24.327
Km39	27.031
k39_9KPR	47.321
Vm33	44.952
Km33	45.589
k41	70.381
Vm42	35.378
Km42	35.386
k43	84.878
k44	86.789
Vm45	31.089
Km45	25.850
k46adj	64.052
k48adj	79.558
k50adj	60.307
Vm51	39.707
Km51	41.591
k52adj	67.618
k53adj	86.442
k54adj	84.860
k57	89.570
k58	72.794
k65adj	74.492
k62adj	65.799
k63	75.383
k64	93.708
k61adj	76.866
k66	81.913
k67adj	73.626
k68adj	82.332
k70	69.426
