# Supplementary material for: Utility of constraints reflecting system stability on analyses for biological models
Source: PLoS Comput Biol. 2022 Sep 9;18(9):e1010441. doi: 10.1371/journal.pcbi.1010441 (PMC9491612; doi:10.1371/journal.pcbi.1010441)
Supplement: S1 Model — SBML file are provided as S1 File. (PDF) [file pcbi.1010441.s007.pdf]

S1 model

The arachidonic acid metabolite pathway model.

The following are the equations indicating rates of the reactions:

re1 = Vm1 * [AA]_PMN * [12-LOX]_PMN / (Km1 * (1 + [15-HETE]_PMN / k1i_15HETE + [15-HPETE]_PMN / k1i_15HPETE + [12-HPETE]_PMN / k1i_12HPETE) + [AA]_PMN),

r*e2* = Vm2 * [AA]_PMN * [15-LOX]_PMN * (1 + [PGE2]_PMN / k2_PGE2) / (Km2 + [AA]_PMN),

r*e3* = Vm3 * [12-HPETE]_PMN * [PHGPx]_PMN / (Km3 + [12-HPETE]_PMN),

r*e4* = Vm4 * [15-HPETE]_PMN * [PHGPx]_PMN / ([15-HPETE]_PMN + Km4),

re5 = Vm5 * [PL]_PMN * [PLA2]_PMN * (1 + [5-HETE]_PMN / k5_5HETE + [LTB4]_PMN / k5_LTB4 + [15-HPETE]_PMN / k5_15HPETE + [12-HPETE]_PMN / k5_12HPETE) / (Km5 + [PL]_PMN),

re6 = Vm6 * [AA]_PMN * [5-LOX]_PMN / (Km6 * (1 + [5-HETE]_PMN / k6i_5HETE + [PGE2]_PMN / k6i_PGE2 + [5-HPETE]_PMN / k6i_5HPETE) + [AA]_PMN),

re8 = Vm8 * [5-HPETE]_PMN * [5-LOX]_PMN * (1 + [LTB4]_PMN / k8_LTB4) / (Km8 * (1 + [15-HPETE]_PMN / k8i_15HPETE + [15-HETE]_PMN / k8i_15HETE + [12-HETE]_PMN / k8i_12HETE + [LTA4H]_PMN / k8i_LTA4H) + [5-HPETE]_PMN),

re9 = Vm9 * [5-HPETE]_PMN * [PHGPx]_PMN / (Km9 + [5-HPETE]_PMN),

re10 = Vm10 * [LTA4H]_PMN * [LTA4H_e]_PMN / (Km10 * (1 + [LTA4H]_PMN / k10i_LTA4H) + [LTA4H]_PMN),

re11 = Vm11 * [LTB4]_PMN * [LTB4_12-HD]_PMN / (Km11 + [LTB4]_PMN),

re12 = Vm12 * [LTB4]_PMN * [CYP4F3]_PMN / (Km12 * (1 + [15-HETE]_PMN / k12i_15HETE + [5-HETE]_PMN / k12i_5HETE) + [LTB4]_PMN),

re13 = k13 * [20-OH-LTB4]_PMN,

re14 = Vm14 * [AA]_PMN * [COX-2]_PMN / (Km14 * (1 + [PGE2]_PMN / k14i_PGE2) + [AA]_PMN),

re15 = Vm15 * [PGH2]_PMN * [PGES]_PMN / (Km15 * (1 + [AA]_PMN / k15i_AA + [15-HETE]_PMN / k15i_15HETE) + [PGH2]_PMN),

re16 = Vm16 * [PGH2]_PMN * [TXAS]_PMN / (Km16 * (1 + [PGH2]_PMN / k16i_PGH2 + [15-HPETE]_PMN / k16i_15HPETE) + [PGH2]_PMN),

re17 = k17 * [TXA2]_PMN,

re18 = k18 * [TXB2]_PMN,

re19 = Vm1 * [AA]_EC * [12-LOX]_EC * k19adj / (Km1 * (1 + [15-HETE]_EC / k1i_15HETE + [15-HPETE]_EC / k1i_15HPETE + [12-HPETE]_EC / k1i_12HPETE) + [AA]_EC),

re20 = Vm2 * [AA]_EC * [15-LOX]_EC * k20adj * (1 + [PGE2]_EC / k2_PGE2) / (Km2 + [AA]_EC),

re21 = Vm3 * [12-HPETE]_EC * [PHGPx]_EC * k21adj / (Km3 + [12-HPETE]_EC),

re22 = Vm4 * [15-HPETE]_EC * [PHGPx]_EC * k22adj / ([15-HPETE]_EC + Km4),

re23 = Vm5 * [PL]_EC * [PLA2]_EC * k23adj * (1 + [15-HPETE]_EC / k5_15HPETE + [12-HPETE]_EC / k5_12HPETE + [PGF2a]_EC / k23_PGF2a) / (Km5 * (1 + [15d-PGJ2]_EC / k23i_15dPGJ2) + [PL]_EC),

re31 = Vm14 * [AA]_EC * [COX-2]_EC * k31adj / (Km14 * (1 + [PGE2]_EC / k14i_PGE2) + [AA]_EC),

re32 = Vm15 * [PGH2]_EC * [PGES]_EC * k32adj / (Km15 * (1 + [AA]_EC / k15i_AA + [15-HETE]_EC / k15i_15HETE + [15d-PGJ2]_EC / k32i_15dPGJ2) + [PGH2]_EC),

re33 = Vm16 * [PGH2]_EC * [TXAS]_EC * k33adj / (Km16 * (1 + [PGH2]_EC / k16i_PGH2 + [15-HPETE]_EC / k16i_15HPETE) + [PGH2]_EC),

re34 = k17 * k34adj * [TXA2]_EC,

re35 = k18 * k35adj * [TXB2]_EC,

re36 = Vm36 * [PGH2]_EC * [PGIS]_EC / (Km36 * (1 + [15-HPETE]_EC / k36i_15HPETE) + [PGH2]_EC),

re37 = k37 * [PGI2]_EC,

re38 = k38 * [6-keto-PGF1a]_EC,

re39 = Vm39 * [PGH2]_EC * [CR]_EC * (1 + [9-KPR]_EC / k39_9KPR) / (Km39 + [PGH2]_EC),

re40 = Vm33 * [PGF2a]_EC * [15-PDGH]_EC / (Km33 + [PGF2a]_EC),

re41 = k41 * [PGE2]_EC,

re42 = Vm42 * [PGH2]_EC * [PGDS]_EC / (Km42 + [PGH2]_EC),

re43 = k43 * [PGD2]_EC,

re44 = k44 * [PGJ2]_EC,

re45 = Vm45 * [PGD2]_EC * [PGFS]_EC / (Km45 + [PGD2]_EC),

re46 = Vm1 * [AA]_PLT * [12-LOX]_PLT * k46adj / (Km1 * (1 + [12-HPETE]_PLT / k1i_12HPETE) + [AA]_PLT),

re48 = Vm3 * [12-HPETE]_PLT * [PHGPx]_PLT * k48adj / (Km3 + [12-HPETE]_PLT),

re50 = Vm5 * [PL]_PLT * [PLA2]_PLT * k50adj * (1 + [12-HPETE]_PLT / k5_12HPETE) / (Km5 + [PL]_PLT),

re51 = Vm51 * [AA]_PLT * [COX-1]_PLT / (Km51 + [AA]_PLT),

re52 = Vm16 * [PGH2]_PLT * [TXAS]_PLT * k52adj / (Km16 * (1 + [PGH2]_PLT / k16i_PGH2) + [PGH2]_PLT),

re53 = k17 * k53adj * [TXA2]_PLT,

re54 = k18 * k54adj * [TXB2]_PLT,

re55 = k55 * [12-HETE]_PMN,

re56 = k56 * [15-HETE]_PMN,

re57 = k57 * [20-COOH-LTB4]_PMN,

re58 = k58 * [12-keto-LTB4]_PMN,

re59 = k59 * [5-HETE]_PMN,

re60 = k60 * [11-dehydro-TXB2]_PMN,

re61 = k55 * [12-HETE]_EC * k65adj,

re62 = k56 * [15-HETE]_EC * k62adj,

re63 = k63 * [15-keto-PGF2a]_EC,

re64 = k64 * [6-keto-PGE1]_EC,

re65 = k60 * k61adj * [11-dehydro-TXB2]_EC,

re66 = k66 * [15d-PGJ2]_EC,

re67 = k55 * k67adj * [12-HETE]_PLT,

re68 = k60 * k68adj * [11-dehydro-TXB2]_PLT,

re69 = k69 * [PGE2]_PMN,

re70 = k70 * [11-epi-PGF2a]_EC.

The ODE system is composed as follows:

d[12-HETE]_PLT/dt = *re48 - re67*,

d[12-HPETE]_PLT/dt = *re46 - re48*,

d[AA]_PLT/dt = *re50 - re46 - re51*,

d[PL]_PLT/dt = 0,

d[15-HPETE]_EC/dt = *re20 - re22*,

d[15-HETE]_EC/dt = *re22 - re62*,

d[5-HPETE]_PMN/dt = *re6 - re8 - re9*,

d[LTA4H]_PMN/dt = *re8 - re10*,

d[LTB4]_PMN/dt = *re10 - re11 - re12*,

d[5-HETE]_PMN/dt = *re9 - re59*,

d[20-OH-LTB4]_PMN/dt = *re12 - re13*,

d[20-COOH-LTB4]_PMN/dt = *re13 - re57*,

d[12-keto-LTB4]_PMN/dt = *re11 - re58*,

d[PGH2]_PLT/dt = *re51 - re52*,

d[PGE2]_EC/dt = *re32 - re41*,

d[TXA2]_PLT/dt = *re52 - re53*,

d[TXB2]_PLT/dt = *re53 - re54*,

d[11-dehydro-TXB2]_PLT/dt = *re54 - re68*,

d[PHGPx]_PLT/dt = 0,

d[12-LOX]_PLT/dt = 0,

d[15-LOX]_EC/dt = 0,

d[5-LOX]_PMN/dt = 0,

d[LTA4H_e]_PMN/dt = 0,

d[CYP4F3]_PMN/dt = 0,

d[LTB4_12-HD]_PMN/dt = 0,

d[COX-2]_EC/dt = 0,

d[PGES]_EC/dt = 0,

d[TXAS]_PLT/dt = 0,

d[PLA2]_PLT/dt = 0,

d[PGI2]_EC/dt = *re36 - re37*,

d[6-keto-PGF1a]_EC/dt = *re37 - re38*,

d[6-keto-PGE1]_EC/dt = *re38 - re64*,

d[PGF2a]_EC/dt = *re39 + re41 - re40*,

d[15-keto-PGF2a]_EC/dt = *re40 - re63*,

d[PGD2]_EC/dt = *re42 - re43 - re45*,

d[PGJ2]_EC/dt = *re43 - re44*,

d[15d-PGJ2]_EC/dt = *re44 - re66*,

d[11-epi-PGF2a]_EC/dt = *re45 - re70*,

d[PGIS]_EC/dt = 0,

d[PGDS]_EC/dt = 0,

d[PGFS]_EC/dt = 0,

d[CR]_EC/dt = 0,

d[9-KPR]_EC/dt = 0,

d[15-PDGH]_EC/dt = 0,

d[COX-1]_PLT/dt = 0,

d[12-HETE]_PMN/dt = *re3 - re55*,

d[12-HPETE]_PMN/dt = *re1 - re3*,

d[AA]_PMN/dt = *re5 - re1 - re2 - re6 - re14*,

d[PL]_PMN/dt = 0,

d[15-HPETE]_PMN/dt = *re2 - re4*,

d[15-HETE]_PMN/dt = *re4 - re56*,

d[PGH2]_PMN/dt = *re14 - re15 - re16*,

d[PGE2]_PMN/dt = *re15 - re69*,

d[TXA2]_PMN/dt = *re16 - re17*,

d[TXB2]_PMN/dt = *re17 - re18*,

d[11-dehydro-TXB2]_PMN/dt = *re18 - re60*,

d[PHGPx]_PMN/dt = 0,

d[12-LOX]_PMN/dt = 0,

d[15-LOX]_PMN/dt = 0,

d[COX-2]_PMN/dt = 0,

d[PGES]_PMN/dt = 0,

d[TXAS]_PMN/dt = 0,

d[PLA2]_PMN/dt = 0,

d[12-HPETE]_EC/dt = *re19 - re21*,

d[12-HETE]_EC/dt = *re21 - re61*,

d[12-LOX]_EC/dt = 0,

d[PHGPx]_EC/dt = 0,

d[PL]_EC/dt = 0,

d[PLA2]_EC/dt = 0,

d[PGH2]_EC/dt = *re31 - re32 - re33 - re36 - re39 - re42*,

d[AA]_EC/dt = *re23 - re19 - re20 - re31*,

d[TXAS]_EC/dt = 0,

d[TXA2]_EC/dt = *re33 - re34*,

d[TXB2]_EC/dt = *re34 - re35*,

d[11-dehydro-TXB2]_EC/dt = *re35 - re65*.
