# Supplementary material for: Utility of constraints reflecting system stability on analyses for biological models
Source: PLoS Comput Biol. 2022 Sep 9;18(9):e1010441. doi: 10.1371/journal.pcbi.1010441 (PMC9491612; doi:10.1371/journal.pcbi.1010441)
Supplement: S1 Information — The theorem and its proof were descripted. (PDF) [file pcbi.1010441.s010.pdf]

S1 Information

Mathematical background of objective function for basin stability

Theorem.

Let $D^N = \{x \in R^N \mid |x - x^*| < r\}$ an *N*-dimensional open disc around a fixed point $x^* \in R^N$ in *N*-dimensional Euclidean space, where *r*>0 is the radius of the disc, and $\frac{\mathrm{d}x}{\mathrm{d}t} = f(x)$ is a continuously differentiable vector field on an open set containing D^N. Assume x^* is a fixed point of the flow $f(x)$, namely $f(x^*) = 0$. Let $D_f(x)$ the Jacobian matrix of $f(x)$ at x, and let matrix $M(x) = D_f(x) + {}^tD_f(x)$ be the sum of $D_f(x)$ and its transpose matrix ${}^tD_f(x)$.

If assumption

(A) $\lambda_{\max} := \max_{\mathrm{x} \in D^N} \sigma(M(x)) \leq 0$ (1)

holds, then

(i) any trajectory $\phi_t(x_0)$ with its initial condition in $x_0 \in D^N$ stays in D^N for $t \to \infty$.

If assumption

(A') $\lambda_{\max} := \max_{\mathrm{x} \in D^N} \sigma(M(x)) < 0$. (2)

holds, then

(ii) any trajectory $\phi_t(x_0)$ with its initial condition in D^N converges exponentially to the fixed point x^*.

Proof.

For the initial condition $x_0 = x^*$, it is obvious that the results (i) and (ii) hold.

Now let $x_0 \in D^N$ be an arbitrary point in the disc D^N that satisfies $x_0 \neq x^*$. We consider the evolution of the linear line $\gamma_0(\tau) = \{x^* + \tau(x_0 - x^*) \mid \tau \in [0, 1]\}$ by the flow. Since $f(x)$

is a continuously differentiable vector field on D^N, there exists a finite time $T_0 > 0$ such that the following solution exists:

$$\gamma_t(\tau) = \{\phi_t(x^* + \tau(x_0 - x^*)) \mid \tau \in [0,1], -T_0 < t < T_0\} \tag{3}$$

where $(t, x) \mapsto \phi_t(x) : R^N \times R \to R^N$ is the flow of the vector field $f(x)$ that satisfies,

$\frac{\mathrm{d}\phi_t(x_0)}{\mathrm{d}t} = f(\phi_t(x_0)),\ \phi_{t=0}(x_0) = x_0.$

The arc-length $L(\gamma_t(\tau))$ of the curve $\gamma_t(\tau)$ at time *t* is given by

$L(\gamma_t) = \int_0^1 \left|\frac{\mathrm{d}\gamma_t(\tau)}{\mathrm{d}\tau}\right| d\tau.$

Since the uniqueness of the solution is assured by the continuously differentiable property of $f(x)$, $\left|\frac{\mathrm{d}\gamma_t(\tau)}{\mathrm{d}\tau}\right| \neq 0$ for any τ and t. Now we consider the time-derivative of $L(\gamma_t)$ as follows:

$$\frac{\mathrm{d}}{\mathrm{d}t}L(\gamma_t) = \frac{\mathrm{d}}{\mathrm{d}t}\int_0^1 \left|\frac{\mathrm{d}\gamma_t(\tau)}{\mathrm{d}\tau}\right| d\tau = \int_0^1 \left(\frac{\mathrm{d}}{\mathrm{d}t}\left|\frac{\mathrm{d}\gamma_t(\tau)}{\mathrm{d}\tau}\right|\right) d\tau$$

$$= \int_0^1 \left(\frac{\mathrm{d}}{\mathrm{d}t}\left({}^{\mathrm{t}}\!\left(\frac{\mathrm{d}\gamma_t(\tau)}{\mathrm{d}\tau}\right) \cdot \frac{\mathrm{d}\gamma_t(\tau)}{\mathrm{d}\tau}\right)^{\frac{1}{2}}\right) d\tau$$

$$= \int_0^1 \left(\frac{1}{2}\left({}^{\mathrm{t}}\!\left(\frac{\mathrm{d}\gamma_t(\tau)}{\mathrm{d}\tau}\right) \cdot \frac{\mathrm{d}\gamma_t(\tau)}{\mathrm{d}\tau}\right)^{-\frac{1}{2}} \left(2 \cdot {}^{\mathrm{t}}\!\left(\frac{\mathrm{d}}{\mathrm{d}t}\frac{\mathrm{d}\gamma_t(\tau)}{\mathrm{d}\tau}\right) \cdot \frac{\mathrm{d}\gamma_t(\tau)}{\mathrm{d}\tau}\right)\right) d\tau$$

$$= \int_0^1 \left(\frac{1}{\left|\frac{\mathrm{d}\gamma_t(\tau)}{\mathrm{d}\tau}\right|}\ {}^{\mathrm{t}}\!\left(\frac{\mathrm{d}}{\mathrm{d}t}\frac{\mathrm{d}\gamma_t(\tau)}{\mathrm{d}\tau}\right) \cdot \frac{\mathrm{d}\gamma_t(\tau)}{\mathrm{d}\tau}\right) d\tau. \tag{4}$$

Since $\gamma_t(\tau) = \phi_t(x^* + \tau(x_0 - x^*))$,

$$\frac{\mathrm{d}}{\mathrm{d}t}\frac{\mathrm{d}\gamma_t(\tau)}{\mathrm{d}\tau} = \frac{\mathrm{d}}{\mathrm{d}\tau}\frac{\mathrm{d}\gamma_t(\tau)}{\mathrm{d}t} = \frac{\mathrm{d}}{\mathrm{d}\tau}f\big(\gamma_t(\tau)\big) = D_f(\gamma_t(\tau)) \cdot \frac{\mathrm{d}\gamma_t(\tau)}{\mathrm{d}\tau}, \tag{5}$$

where D_f is the Jacobian matrix of $f(x)$ along the trajectory $x(t)$. Substituting to Equ. (4) yields

$$\frac{\mathrm{d}}{\mathrm{d}t}L(\gamma_t) = \int_0^1 \left(\frac{1}{\left|\frac{\mathrm{d}\gamma_t(\tau)}{\mathrm{d}\tau}\right|} {}^{\mathrm{t}}\left(\frac{\mathrm{d}}{\mathrm{d}t}\frac{\mathrm{d}\gamma_t(\tau)}{\mathrm{d}\tau}\right) \cdot \frac{\mathrm{d}\gamma_t(\tau)}{\mathrm{d}\tau} \right) d\tau$$

$$= \int_0^1 \left(\frac{1}{\left|\frac{\mathrm{d}\gamma_t(\tau)}{\mathrm{d}\tau}\right|} {}^{\mathrm{t}}\left(\frac{\mathrm{d}\gamma_t(\tau)}{\mathrm{d}\tau}\right) \cdot {}^{\mathrm{t}}D_f\big(\gamma_t(\tau)\big) \cdot \frac{\mathrm{d}\gamma_t(\tau)}{\mathrm{d}\tau} \right) d\tau$$

$$= \int_0^1 \left(\left|\frac{\mathrm{d}\gamma_t(\tau)}{\mathrm{d}\tau}\right| {}^{\mathrm{t}}\big(u_t(\tau)\big) \cdot D_f(\gamma_t(\tau)) \cdot u_t(\tau) \right) d\tau$$

$$= \int_0^{L(\gamma_t)} \left({}^{\mathrm{t}}\big(u_t(s)\big) \cdot D_f(\gamma_t(s)) \cdot u_t(s) \right) ds = \frac{1}{2}\int_0^{L(\gamma_t)} \left({}^{\mathrm{t}}\big(u_t(s)\big) \cdot \big(D_f(\gamma_t(s) + \right.$$

$$\left. {}^{\mathrm{t}}D_f(\gamma_t(s))\big) \cdot u_t(s) \right) ds = \frac{1}{2}\int_0^{L(\gamma_t)} \left({}^{\mathrm{t}}\big(u_t(s)\big) \cdot M(\gamma_t(s)) \cdot u_t(s) \right) ds \qquad (6)$$

where $u_t(\tau) = \frac{1}{\left|\frac{\mathrm{d}\gamma_t(\tau)}{\mathrm{d}\tau}\right|} \cdot \frac{\mathrm{d}\gamma_t(\tau)}{\mathrm{d}\tau}$, $\frac{ds}{d\tau} = \left|\frac{\mathrm{d}\gamma_t(\tau)}{\mathrm{d}\tau}\right|$, $\gamma_t(s) = \gamma_t\big(\tau(s)\big)$ and $u_t(s) = u_t(\tau(s))$.

Using $\lambda_{\max} = \max_{x \in D^N} \sigma(M(x))$ and $|u_t(s)| = 1$, we obtain

$$\frac{\mathrm{d}}{\mathrm{d}t}L(\gamma_t) \le \frac{\lambda_{\max}}{2} \cdot L(\gamma_t) \qquad (7)$$

From the assumption (A) $\lambda_{\max} = \max_{x \in D^N} \sigma(M(x)) \le 0$, we further obtain

$$\frac{\mathrm{d}}{\mathrm{d}t}L(\gamma_t) \le \frac{\lambda_{\max}}{2} \cdot L(\gamma_t) \le 0 \qquad (8)$$

From $0 \le |\phi_t(x_0) - x^*| \le L(\gamma_t)$, we conclude $\frac{\mathrm{d}}{dt}|\phi_t(x_0) - x^*| \le 0$. We initially assumed $x_0 \in D^N$ to be an arbitrary point in D^N. Thus, any orbit starting from any initial condition in the disc D^N does not escape from the disc D^N as time evolves, however stays within it.

Next, we consider the case (A') $\lambda_{\max} := \max_{x \in D^N} \sigma(M(x)) < 0$. Since any orbit starting from any initial condition in the disc D^N does not escape from the disc D^N, we can assume the existence and uniqueness of the following solution:

$\gamma_t(\tau) = \{\phi_t(x^* + \tau(x_0 - x^*)) \mid \tau \in [0,1], -T_0 < t < +\infty\}$.

Following the same arguments from (3) to (7) yields

$$\frac{\mathrm{d}}{\mathrm{d}t} L(\gamma_t) \leq \frac{\lambda_{\max}}{2} \cdot L(\gamma_t) \, .$$

Thus,

$$|\phi_t(x_0) - x^*| \leq L(\gamma_t) \leq \mathrm{e}^{\frac{\lambda_{\max} \cdot t}{2}} |\phi_{t=0}(x_0) - x^*|,$$

which means any initial condition x_0 converges to the fixed point x^* exponentially.
