# Supplementary material for: Utility of constraints reflecting system stability on analyses for biological models
Source: PLoS Comput Biol. 2022 Sep 9;18(9):e1010441. doi: 10.1371/journal.pcbi.1010441 (PMC9491612; doi:10.1371/journal.pcbi.1010441)
Supplement: S2 Information — (PDF) [file pcbi.1010441.s011.pdf]

S2 Information

Modification of the global CNM to intensify expansion of searchable areas

In this study, we assumed that the essential dynamic properties of a system of interest could be observed in the simulation results using multiple parameter sets within the allowable parameter space. To securely extract the essential features of the system behaviors, it is necessary to acquire multiple parameter sets covering the entire allowable space. Thus, we used the global CNM proposed by Aoki *et al.* as the basement algorithm (1). Global CNM consists of two stages. In the first stage (CNM stage), the gradient of the objective function is approximated by a hyperplane fitted to the objective function values, calculated with the "cluster" of parameter sets, in place of individual tangential planes. Subsequently, the cluster of parameter sets is shifted along the hyperplane, and the subroutine is iterated until convergence. In the later stage, each parameter set is individually optimized using a quasi-Newton method with a modification to expand the searchable area. This two-stage approach is directly applicable in cases where the shape of the solution space is not complicated. However, we often faced divergence during the optimization using CNM or usual quasi-Newton methods presumably owing to the complexity of our joint objective function with the biological system models used in this study. Thus, we modified the global CNM as follows. In the first stage, rather than performing multiple iterations until strict convergence, we collected the parameter sets that satisfied moderate conditions from the cluster of parameter sets at each step and iterated until the number of collected parameter sets reached the preset number (Fig. S2A). This modification helped avoid non-convergence or excess computing costs owing to excessive iteration steps. In the later stage (globalization stage) of the original global CNM, each parameter set is separately optimized by a modified Broyden's method, which is a type of quasi-Newton method. In the earlier iteration cycles, the direction of shift at each iteration step was determined by the direction calculated using the approximated Hessian matrix of the

objective function as usual. In the later iteration cycles when the value of the objective function nears optimal, the direction of the shift is modified by adding random noise perpendicular to the Hessian matrix-based direction. However, the expanding effect of this modification was insufficient in cases where the shape of the allowable parameter space was complicated, as this implementation did not find an entire solution space in some problems. Therefore, we further modified the globalization stage as follows. Once a converged parameter set was obtained by the modified quasi-Newton method, stochastic noises were added to the obtained parameter set to become more distant from the initial parameter set (see Step **3** in "Entire algorithm of TEAPS"). Then, using the noise-added parameter set as the initial parameter set, optimization using the modified quasi-Newton method was performed again. By repeating these noise addition and optimization cycles, we can expect the distribution of the parameter sets to spread more widely (Fig. S2B). In addition to this expanding procedure, we changed the basement quasi-Newton method to the limited memory-BFGS (L-BFGS) method in the globalization stage to handle large-scale models with low memory.

References

1. Aoki Y, Hayami K, Sterck HD, Konagaya A. Cluster Newton Method for Sampling Multiple Solutions of Underdetermined Inverse Problems: Application to a Parameter Identification Problem in Pharmacokinetics. SIAM Journal on Scientific Computing. 2014;36(1):B14-B44.
2. Konagaya YAKHHDSA. NII technical report: Cluster Newton Method for Sampling Multiple Solutions of an Underdetermined Inverse Problem: Parameter Identification for Pharmacokinetics. Tokyo, Japan: National Institute of Informatics; 2011. p. v.
