# Supplementary material for: Utility of constraints reflecting system stability on analyses for biological models
Source: PLoS Comput Biol. 2022 Sep 9;18(9):e1010441. doi: 10.1371/journal.pcbi.1010441 (PMC9491612; doi:10.1371/journal.pcbi.1010441)
Supplement: S3 Information — (PDF) [file pcbi.1010441.s012.pdf]

S3 Information
Entire algorithm of TEAPS

Throughout the entire algorithm, search over the parameter space of $\boldsymbol{f}$ was performed in a logarithmic scale.

1) Cluster Newton method (CNM) with modifications.

The details of the original CNM are described in the previous report (1, 2).

1-1) Preparation of initial parameter sets and determination of targets.

Randomly generate an initial cluster of N_{pc} points $\left\{\boldsymbol{\mu}_{\cdot j}^{(0)} \in \boldsymbol{R}^{N_{\mathrm{p}}}\right\}_{j=1}^{N_{\mathrm{pc}}}$ from a uniform distribution over a box defined by the following inequalities:

$$\mu_{i,\mathrm{low}} < \mu_{ij}^{(0)} < \mu_{i,\mathrm{up}} \quad \text{for } i = 1,2,\dots,N_{\mathrm{p}},\ j = 1,2,\dots,N_{\mathrm{pc}},$$

where $\mu_{i,\mathrm{low}}$ and $\mu_{i,\mathrm{up}}$ are arbitrary determined bounds of initial distribution of the i th parameter, and N_{p} is the dimension of the parameter space. The number of points in the cluster, N_{pc}, is set to satisfy $N_{\mathrm{pc}} \geq (N_{\mathrm{p}} + 1)N_{\mathrm{div}}$ so that one can construct a linear approximation to N_{t} dimensional vector function $\boldsymbol{h}(\boldsymbol{\mu})$ using its values at all points in a subcluster, where N_{div} is the subdividing number of the cluster, N_{t} is the number of constraints (targets) that the parameter set satisfies, $\boldsymbol{h}$ is the objective function defined by the BSR condition s.t. $\boldsymbol{h} = \boldsymbol{h_0}\left(O_{\mathrm{fix}}(\boldsymbol{\mu}), O_{\mathrm{basin}}(\boldsymbol{\mu}), O_{\mathrm{relax}}(\boldsymbol{\mu})\right) = [O_{\mathrm{fix}}(\boldsymbol{\mu}), O_{\mathrm{basin}}(\boldsymbol{\mu}), O_{\mathrm{relax}}(\boldsymbol{\mu})]^{\mathrm{T}}$. We denote by $\boldsymbol{C}^{(0)}$ the concatenated matrix of the vectors in the cluster as $\boldsymbol{C}^{(0)} = \left[\boldsymbol{\mu}_{\cdot 1}^{(0)}, \boldsymbol{\mu}_{\cdot 2}^{(0)}, \dots, \boldsymbol{\mu}_{\cdot N_{\mathrm{pc}}}^{(0)}\right] \in \boldsymbol{R}^{N_{\mathrm{p}}\times N_{\mathrm{pc}}}$, where [ ] denotes concatenation.

1-2) Definition of BSR objective functions.

Set a target fixed point $\boldsymbol{x}^* = ({x^*}_1, {x^*}_2, \dots, {x^*}_{N_{\mathrm{phase}}})^{\mathrm{T}}$ so that one can calculate $O_{\mathrm{fix}}(\boldsymbol{\mu}) = \|\boldsymbol{f}(\boldsymbol{x}^*, \boldsymbol{\mu})\|_2$, where $\| \ \|_2$ denotes L2 norm and N_{phase} is the dimension of phase space.

Set a target value ${\lambda^*}_{\mathrm{target}}$ so that one can calculate $O_{\mathrm{relax}}(\boldsymbol{\mu}) = \mathrm{ReLU}\left(\max_{\mathrm{Re}(\lambda^*)\neq 0} \mathrm{Re}(\lambda^*) - {\lambda^*}_{\mathrm{target}}\right)$, where $\max_{\mathrm{Re}(\lambda^*)\neq 0} \mathrm{Re}(\lambda^*)$ is the largest real part of nonzero eigenvalues of Jacobian matrix of $\boldsymbol{f}(\boldsymbol{x}, \boldsymbol{\mu})$ at $\boldsymbol{x}^*$.

Set a target stable region $\boldsymbol{B}$ around $\boldsymbol{x}^*$. In this study, we set $\boldsymbol{B} = \{\boldsymbol{x} \mid |(x_n - {x^*}_n)/{x^*}_n| \leq (d_{\max} - 1) \ \text{for } n = 1,2,\dots,N_{\mathrm{phase}}.\}$, where $d_{\max}$ is a value defining maximum acceptable fold change from $\boldsymbol{x}^*$.

1-3) Initiation of iteration starting with $k = 0$ and an empty matrix $C_{\mathrm{store}} \in \boldsymbol{R}^{N_{\mathrm{p}}\times 0}$.

While the number of columns in $\boldsymbol{C}_{\mathrm{store}} < N_{\mathrm{pc}}$:

1-3-1) Subdivide N_{pc} vectors in the cluster $\boldsymbol{C}^{(k)}$, $\boldsymbol{\mu}_{\cdot 1}^{(k)}, \boldsymbol{\mu}_{\cdot 2}^{(k)}, \dots, \boldsymbol{\mu}_{\cdot N_{\mathrm{pc}}}^{(k)}$, into equal size of N_{div} subclusters with random allocation including $N_{\mathrm{psc}} = N_{\mathrm{pc}}/N_{\mathrm{div}}$ vectors, s.t.

$$\boldsymbol{C}_{k_{sc}}^{(k)} = \left[\boldsymbol{\mu}_{k_{sc},\cdot 1}^{(k)} \boldsymbol{\mu}_{k_{sc},\cdot 2}^{(k)} \dots, \boldsymbol{\mu}_{k_{sc},\cdot N_{\mathrm{psc}}}^{(k)}\right] \in \boldsymbol{R}^{N_{\mathrm{p}}\times N_{\mathrm{psc}}}$$

where $k_{sc} = 1,2,\dots,N_{\mathrm{div}}$, and $\boldsymbol{\mu}_{k_{sc},\cdot}^{(k)} \in \left\{\boldsymbol{\mu}_{\cdot j}^{(k)}\right\}_{j=1}^{N_{\mathrm{psc}}}$.

1-3-2) Prepare finite observation points $\boldsymbol{X}^{(k_{\mathrm{b}},k)} = \left\{\boldsymbol{x}_m \mid x_{m,n} = \left(1 + a_{k_{\mathrm{b}}}\, d_{\max}\right){x^*}_j \ \text{for } m = 1,2,\dots,N_{\mathrm{obs}},\ n = 1,2,\dots,N_{\mathrm{phase}}\right\}$ so that one can calculate $O_{\mathrm{basin}}(\boldsymbol{\mu}) = \mathrm{ReLU}\left(\max_{\boldsymbol{x}\in\boldsymbol{X}^{(k_{\mathrm{b}},k)}} \lambda\left(M(\boldsymbol{x})\right)\right)$, where k_{b} is an index defining

the broadness of distribution of observation points and N_{obs} is the number of observation points. The value $a_{k_{\text{b}}}$ was taken from a uniform distribution over $[-(0.4 \times k_{\text{b}} - 0.2)^2, (0.4 \times k_{\text{b}} - 0.2)^2]$. In this step, set $k_{\text{b}} = 1$.

1-3-3) Subcluster based linear approximation.

For $k_{sc} = 1,2,\dots,N_{\text{div}}$:

1-3-3-1) Let $\boldsymbol{y}^*$ be the vector consisting of the target values for $\boldsymbol{h_0}$ to be satisfied. In order to assure well-posedness in the linear least squares problem in the later process, generate randomly perturbed target vectors $\left\{\boldsymbol{y}^*_{\cdot j'}\right\}_{j'=1}^{N_{\text{psc}}}$ near $\boldsymbol{y}^*$ that satisfies,

$$\max_{l=1,2,\dots,N_{\text{t}}} \left| \frac{y^*_{lj'} - y_{\text{target},l}}{y_{\text{target},l}} \right| < \eta$$

with $\eta = 0.05$, and $y_{\text{target},l} = y^*_l$ when $y^*_l \neq 0$, otherwise $y_{\text{target},l} = \varepsilon$, where ε is a positive small value which is determined by considering computation errors in an actual implementation.

1-3-3-2) Solve the following forward problem for each $\boldsymbol{\mu}^{(k)}_{k_{sc},\cdot j'}$:

$$\boldsymbol{y}^{(k)}_{k_{sc},\cdot j'} = \boldsymbol{h}\left(\boldsymbol{\mu}^{(k)}_{k_{sc},\cdot j'}\right) \quad \text{for } j' = 1,2,\dots,N_{\text{psc}}$$

with $\boldsymbol{Y}^{(k)}_{k_{sc}} = \left[\boldsymbol{y}^{(k)}_{k_{sc},\cdot 1}, \boldsymbol{y}^{(k)}_{k_{sc},\cdot 2} \dots, \boldsymbol{y}^{(k)}_{k_{sc},\cdot N_{\text{psc}}}\right] \in \boldsymbol{R}^{N_{\text{t}} \times N_{\text{psc}}}$.

1-3-3-3) Construct a linear approximation of $\boldsymbol{h}$ as

$$\boldsymbol{h}^{(k)}_{k_{sc}}(\boldsymbol{\mu}) \approx \boldsymbol{A}^{(k)}_{k_{sc}}\boldsymbol{\mu} + \boldsymbol{y}^{(k)}_{k_{sc},0},$$

by fitting a hyperplane $\boldsymbol{A}^{(k)}_{k_{sc}}\boldsymbol{\mu} + \boldsymbol{y}^{(k)}_{k_{sc},0}$ to $\left\{\left(\boldsymbol{\mu}^{(k)}_{k_{sc},\cdot j'}, \boldsymbol{y}^{(k)}_{k_{sc},\cdot j'}\right)\right\}_{j'=1}^{N_{\text{psc}}}$.

The slope matrix $\boldsymbol{A}^{(k)}_{k_{sc}}$ and the shift constant $\boldsymbol{y}^{(k)}_{k_{sc},0}$ can be found as the least squares solution of an overdetermined system of linear equations:

$$\min_{\boldsymbol{A}^{(k)}_{k_{sc}} \in \boldsymbol{R}^{N_{\text{t}} \times N_{\text{p}}}, \boldsymbol{y}^{(k)}_{k_{sc},0} \in \boldsymbol{R}^{N_{\text{t}}}} \left\| \boldsymbol{Y}^{(k)}_{k_{sc}} - \left(\boldsymbol{A}^{(k)}_{k_{sc}}\boldsymbol{C}^{(k)}_{k_{sc}} + \boldsymbol{Y}^{(k)}_{k_{sc},0}\right) \right\|_F,$$

where $\boldsymbol{Y}^{(k)}_{k_{sc},0}$ is a $N_{\text{t}} \times N_{\text{psc}}$ matrix whose columns are all $\boldsymbol{y}^{(k)}_{k_{sc},0}$, and $\| \quad \|_F$ denotes the Frobenius norm.

For each $\boldsymbol{\mu}^{(k)}_{k_{sc},\cdot j'}$, find an update vector $\boldsymbol{s}^{(k)}_{k_{sc},\cdot j'}$ as

$$\boldsymbol{s}^{(k)}_{k_{sc},\cdot j'} = \min_{\boldsymbol{s} \in \boldsymbol{R}^{N_{\text{p}}}} \boldsymbol{s} \text{ s. t. } \boldsymbol{y}^* = \boldsymbol{A}^{(k)}_{k_{sc}}\left(\boldsymbol{\mu}^{(k)}_{k_{sc},\cdot j'} + \boldsymbol{s}\right) + \boldsymbol{y}^{(k)}_{k_{sc},0}.$$

1-3-3-4) Update $\boldsymbol{\mu}^{(k)}_{k_{sc},\cdot j'}$.

$$\boldsymbol{\mu}^{(k+1)}_{k_{sc},\cdot j'} = \boldsymbol{\mu}^{(k)}_{k_{sc},\cdot j'} + \boldsymbol{s}^{(k)}_{k_{sc},\cdot j'} \quad \text{for } j' = 1,2,\dots,N_{\text{psc}}.$$

While $\boldsymbol{\mu}^{(k+1)}_{k_{sc},\cdot j'} \notin \chi$, where χ is an exploring space for $\boldsymbol{\mu}$:

$$\boldsymbol{s}^{(k)}_{k_{sc},\cdot j'} \leftarrow \frac{1}{2}\boldsymbol{s}^{(k)}_{k_{sc},\cdot j'},$$

$$\boldsymbol{\mu}^{(k+1)}_{k_{sc},\cdot j'} \leftarrow \boldsymbol{\mu}^{(k+1)}_{k_{sc},\cdot j'} - \boldsymbol{s}^{(k)}_{k_{sc},\cdot j'},$$

where $a \leftarrow b$ indicates substitution of b for a.

End while.

1-3-3-5) Combine $\boldsymbol{\mu}^{(k+1)}_{k_{sc},\cdot j}$ to yield an updated subcluster as follows:

$$\boldsymbol{C}^{(k+1)}_{k_{sc}} = \left[\boldsymbol{\mu}^{(k+1)}_{k_{sc},\cdot 1}, \boldsymbol{\mu}^{(k+1)}_{k_{sc},\cdot 2}, \dots, \boldsymbol{\mu}^{(k+1)}_{k_{sc},\cdot N_{\text{psc}}}\right] \in \boldsymbol{R}^{N_{\text{p}} \times N_{\text{psc}}}.$$

End for.

1-3-4) Combine the subclusters to yield a new cluster $C^{(k+1)}$ as follows:

$$\boldsymbol{C}^{(k+1)} = \left[\boldsymbol{C}_1^{(k+1)}, \boldsymbol{C}_2^{(k+1)}, \dots, \boldsymbol{C}_{N_{\text{div}}}^{(k+1)}\right] \in \boldsymbol{R}^{N_{\text{p}} \times N_{\text{pc}}}.$$

Rename each column of $C^{(k+1)}$ as $\boldsymbol{\mu}_{\cdot j}^{(k+1)}$.

1-3-5) Collect $\boldsymbol{\mu}_{\cdot j}^{(k+1)}$ moderately close to the target condition. This step is added to the original CNM.

For $j = 1,2, \dots, N_{\text{pc}}$:

If $\|\boldsymbol{h}\left(\boldsymbol{\mu}_{\cdot j}^{(k+1)}\right) - \boldsymbol{y}^*\|_1 < c\zeta$

$$\boldsymbol{C}_{\text{store}} \leftarrow \left[\boldsymbol{C}_{\text{store}}, \boldsymbol{\mu}_{\cdot j}^{(k+1)}\right],$$

where $\| \quad \|_1$ denotes L1 norm.

End if.

End for.

In the above, ζ is the acceptable tolerance error, and c is a constant which is increased when accumulation into $\boldsymbol{C}_{\text{store}}$ is not proceeding for several iterations.

If the number of columns in $\boldsymbol{C}_{\text{store}} < N_{\text{pc}}$:

Update k as $k \leftarrow k + 1$.

End if.

End while.

1-4) In 1-3), we obtained parameter sets which were moderately close to the target situation prepared as a matrix $\boldsymbol{C}_{\text{store}}$ including l column vectors. Rename each column vector as $\boldsymbol{\mu}_{\cdot j}^{*(1)}$.

2) Further optimize each parameter set obtained in **1)** using a modified L-BFGS method.

Optimize $\boldsymbol{\mu}_{\cdot j}^{*(1)}$ to minimize the objective function h and simultaneously expanding the distribution of $\boldsymbol{\mu}_{\cdot j}^{*(1)}$ by L-BFGS method with implementation of previously reported idea as global CNM (1). In the current study, we changed the objective function in this step to h_1, which is the weighted sum of BSR condition factors:

$h = h_1(O_{\text{fix}}(\boldsymbol{\mu}), O_{\text{basin}}(\boldsymbol{\mu}), O_{\text{relax}}(\boldsymbol{\mu})) = (O_{\text{fix}})^{W_{\text{fix}}} + (O_{\text{basin}})^{W_{\text{basin}}} + (O_{\text{relax}})^{W_{\text{relax}}}$,

where W_{fix} is exponential weight for O_{fix}, $W_{\text{fix}} = 2$, $W_{\text{basin}} = 1{\sim}2$ and $W_{\text{basin}} = 1{\sim}2$ was used in the current study. For calculation of O_{relax}, the observation points used in the final iteration of **1)** is used.

2-1) Optimize by steepest descent method initially.

Calculate gradient and substitute to the initial direction vector $\boldsymbol{d}_{\cdot j}^{(1)}$as follows:

$$\boldsymbol{g}_{\cdot j}^{(1)} = \text{grad } h\left(\boldsymbol{\mu}_{\cdot j}^{*(1)}\right), \quad \boldsymbol{d}_{\cdot j}^{(1)} = -\boldsymbol{g}_{\cdot j}^{(1)}.$$

Find a step size $a_{\cdot j}^{(1)}$ s.t. $a_{\cdot j}^{(1)} = \underset{a}{\arg\min}\, h\left(\boldsymbol{\mu}_{\cdot j}^{*(1)} + a_{\cdot j}^{(1)} \boldsymbol{d}_{\cdot j}^{(1)}\right)$ by line search.

Update $\boldsymbol{\mu}_{\cdot j}^{*}$:

$$\boldsymbol{\mu}_{\cdot j}^{*(2)} = \boldsymbol{\mu}_{\cdot j}^{*(1)} + \boldsymbol{s}_{\cdot j}^{*(1)},$$

with a shift vector $\boldsymbol{s}_{\cdot j}^{*(1)} = a_{\cdot j}^{(1)} \boldsymbol{d}_{\cdot j}^{(1)}$.

2-2) Optimize by the modified L-BFGS method.

Let $K_2 - 1$ be an iteration limit number.

For $k_2 = 2, \dots, K_2 - 1$, iterate 2-2-2) and 2-2-3) :

2-2-2) Find a direction to minimize objective function by L-BFGS method.

Instead of calculating the actual Hessian matrix $\boldsymbol{H}_{\cdot j}^{(k_2)}$ for $h\left(\boldsymbol{\mu}_{\cdot j}^{*(k_2)}\right)$, the direction vector $\boldsymbol{d}_{\cdot j}^{(k_2)} = -\left(\boldsymbol{H}_{\cdot j}^{(k_2)}\right)^{-1} \boldsymbol{g}_{\cdot j}^{(k_2)}$ was estimated and used for updating $\boldsymbol{\mu}_{\cdot j}^{*(k_2)}$

according to the L-BFGS method, where $\boldsymbol{g}_{\cdot j}^{(k_2)} = \text{grad}\, h\left(\boldsymbol{\mu}_{\cdot j}^{*(k_2)}\right)$. The vector $\boldsymbol{d}_{\cdot j}^{(k_2)}$ is estimated using previous $\hat{p}$ iterations conducting following calculations, where $\hat{p} = \min(k_2 - 1, p)$ and we used $p = 100$ in this study:

Set the two vectors $\boldsymbol{v}_{\cdot j}^{(k_2-1)}$ and $\boldsymbol{q}$ as follows:

$$\boldsymbol{v}_{\cdot j}^{(k_2-1)} = \boldsymbol{g}_{\cdot j}^{(k_2)} - \boldsymbol{g}_{\cdot j}^{(k_2-1)},$$

$$\boldsymbol{q} \leftarrow \boldsymbol{g}_{\cdot j}^{(k_2)}.$$

For $i_{\text{tmp}} = k_2 - 1, \dots, k_2 - \hat{p}$:

$$\alpha_{\cdot j}^{(i_{\text{tmp}})} \leftarrow \frac{\left(\boldsymbol{s}_{\cdot j}^{*(i_{\text{tmp}})}\right)^T \boldsymbol{q}}{\left(\boldsymbol{v}_{\cdot j}^{(i_{\text{tmp}})}\right)^{\mathrm{T}} \boldsymbol{s}_{\cdot j}^{*(i_{\text{tmp}})}}, \qquad \boldsymbol{q} \leftarrow \boldsymbol{q} - \alpha_{\cdot j}^{(i_{\text{tmp}})} \boldsymbol{v}_{\cdot j}^{(i_{\text{tmp}})},$$

End for.

Note that a shift vector $\boldsymbol{s}_{\cdot j}^{*(k_2-1)} = \boldsymbol{\mu}_{\cdot j}^{*(k_2)} - \boldsymbol{\mu}_{\cdot j}^{*(k_2-1)}$ is calculated in 2-2-3-2) and 2-2-3-3) for $k_2 - 1 \geq 2$.

Update $\boldsymbol{q}$ as:

$$\boldsymbol{q} \leftarrow \frac{\left(\boldsymbol{s}_{\cdot j}^{*(k_2-1)}\right)^T \boldsymbol{v}_{\cdot j}^{(k_2-1)}}{\left(\boldsymbol{v}_{\cdot j}^{(k_2-1)}\right)^{\mathrm{T}} \boldsymbol{v}_{\cdot j}^{(k_2-1)}} \boldsymbol{q}.$$

For $i_{\text{tmp}} = k_2 - \hat{p}, \dots, k_2 - 1$:

$$\beta_{\cdot j}^{(i_{\text{tmp}})} \leftarrow \frac{\left(\boldsymbol{v}_{\cdot j}^{*(i_{\text{tmp}})}\right)^T \boldsymbol{q}}{\left(\boldsymbol{v}_{\cdot j}^{(i_{\text{tmp}})}\right)^{\mathrm{T}} \boldsymbol{s}_{\cdot j}^{*(i_{\text{tmp}})}}, \qquad \boldsymbol{q} \leftarrow \boldsymbol{q} + \left(\alpha_{\cdot j}^{(i_{\text{tmp}})} - \beta_{\cdot j}^{(i_{\text{tmp}})}\right) \boldsymbol{s}_{\cdot j}^{*(i_{\text{tmp}})},$$

End for.

Determine the direction vector as:

$$\boldsymbol{d}_{\cdot j}^{(k_2)} = -\boldsymbol{q}.$$

2-2-3) Update $\boldsymbol{\mu}_{\cdot j}^{*(k_2)}$.

To expand the distribution of $\boldsymbol{\mu}^{*(k_2)}$, $\boldsymbol{\mu}_{\cdot j}^{*(k_2)}$ is slid in the direction tangential to the solution set once they are sufficiently close to the solution set, according to following steps.

2-2-3-1)

If $\left\|\boldsymbol{s}_{\cdot j}^{*(k_2-1)}\right\|_2 < \xi$:

Find a vector $\boldsymbol{t}$ randomly s.t. $\boldsymbol{t}^{\mathrm{T}} \boldsymbol{d}_{\cdot j}^{(k_2)} = 0$, $\|\boldsymbol{t}\|_2 = 1$.

Add a tangential shift to $\boldsymbol{d}_{\cdot j}^{(k_2)}$:

$$\boldsymbol{d}_{\cdot j}^{(k_2)} \leftarrow \boldsymbol{d}_{\cdot j}^{(k_2)} + r\boldsymbol{t},$$

where ξ is the value defining the threshold of a closeness to the solution, r is a random size taken from a uniform distribution over $[-d_r, d_r]$. The value $d_r = 10$ is used in this study.

End if.

2-2-3-2)

Find a step size $a_{\cdot j}^{(k_2)}$ s.t. $a_{\cdot j}^{(k_2)} = \arg\min_{a_{\cdot j}^{(k_2)}} h\left(\boldsymbol{\mu}_{\cdot j}^{*(k_2)} + a_{\cdot j}^{(k_2)} \boldsymbol{d}_{\cdot j}^{(k_2)}\right)$ by line search.

Update $\boldsymbol{\mu}_{\cdot j}^{*(k_2)}$ and $\boldsymbol{s}_{\cdot j}^{*(k_2)}$ as follows:

$$\boldsymbol{\mu}_{\cdot j}^{*(k_2+1)} = \boldsymbol{\mu}_{\cdot j}^{*(k_2)} + a_{\cdot j}^{(k_2)} \boldsymbol{d}_{\cdot j}^{(k_2)},$$

$$\boldsymbol{s}_{\cdot j}^{*(k_2)} = a_{\cdot j}^{(k_2)} \boldsymbol{d}_{\cdot j}^{(k_2)}.$$

2-2-3-3)

While $\boldsymbol{\mu}_{\cdot j}^{*(k_2+1)} \notin \chi$:

$$\boldsymbol{s}_{\cdot j}^{*(k_2)} \leftarrow \frac{1}{2} \boldsymbol{s}_{\cdot j}^{*(k_2)}$$

$$\boldsymbol{\mu}_{\cdot j}^{*(k_2+1)} \leftarrow \boldsymbol{\mu}_{\cdot j}^{*(k_2+1)} - \boldsymbol{s}_{\cdot j}^{*(k_2)}$$

End While.

End for.

3) Further expanding of the distribution of $\boldsymbol{\mu}^*$.

For $j = 1, 2, \ldots, N_{\mathrm{pc}}$:

3-1) Calculate the shift from $\boldsymbol{\mu}_{\cdot j}^{*(1)}$ to $\boldsymbol{\mu}_{\cdot j}^{*(K_2)}$as

$$\boldsymbol{s}_{\cdot j}^{(K_2)} = \boldsymbol{\mu}_{\cdot j}^{*(K_2)} - \boldsymbol{\mu}_{\cdot j}^{*(1)}.$$

3-2) Randomly generate $\boldsymbol{t}$ s.t. $\boldsymbol{t}^{\mathrm{T}} \boldsymbol{s}_{\cdot j}^{(K_2)} = 0$, $\|\boldsymbol{t}\|_2 = 1$.

3-3) Add an expansive shift as follows:

$$\boldsymbol{s}_{\cdot j}^{(K_2)} \leftarrow \boldsymbol{s}_{\cdot j}^{(K_2)} + r_{t,j} \operatorname{diag}(\boldsymbol{w}_j) \left(\boldsymbol{t} + r_{s,j} \frac{\boldsymbol{s}_{\cdot j}^{(K_2)}}{\left\| \boldsymbol{s}_{\cdot j}^{(K_2)} \right\|_2} \right)$$

where $r_{t,j}$ and $r_{s,j}$ are random values from uniform distributions over $[0, r_{t,\max}]$ and $[0, 1]$, $r_{t,\max}$ is a positive value to be determined arbitrary, $\boldsymbol{w}_j$ is a vector defining weights for each parameter, and $\operatorname{diag}(\boldsymbol{w}_j)$ denotes a diagonal matrix whose diagonal entries are the vector $\boldsymbol{w}_j$.

To give large shifts to parameters originated from near the center of $\boldsymbol{C}_{\mathrm{store}}$, we used $\boldsymbol{w}_j$ as follows:

$$\boldsymbol{w}_j = \exp\left(- \left[\left(\frac{\mu_{1j}^{*(K_2)} - \overline{\mu_{1\cdot}^{*(1)}}}{b_1 / 2} \right)^2, \cdots, \left(\frac{\mu_{N_{\mathrm{p}}j}^{*(K_2)} - \overline{\mu_{N_{\mathrm{p}}\cdot}^{*(1)}}}{b_{N_{\mathrm{p}}} / 2} \right)^2 \right]^{\mathrm{T}} \right),$$

where $\overline{\mu_{i\cdot}^{*(1)}} = \frac{1}{N_{\mathrm{pc}}} \sum_{j=1}^{N_{\mathrm{pc}}} \mu_{ij}^{*(1)}$ and b_i is a width (broadness) of the exploring space χ for i th parameter for $i = 1, 2, \cdots, N_{\mathrm{p}}$.

3-4) Update $\boldsymbol{\mu}_{\cdot j}^{*(K_2)}$ as $\boldsymbol{\mu}_{\cdot j}^{*(K_2+1)} = \boldsymbol{\mu}_{\cdot j}^{*(1)} + \boldsymbol{s}_{\cdot j}^{(K_2)}$

End for.

4) Repeat **2)** and **3)** for several times as initial $\boldsymbol{\mu}_{\cdot j}^{*(1)}$ is set to $\boldsymbol{\mu}_{\cdot j}^{*(K_2+1)}$.

5) Optimize the expanded distribution to the fixed point condition by minimizing another objective function $h = h_2(O_{\mathrm{fix}}) = \|\boldsymbol{f}(\boldsymbol{x}^*, \boldsymbol{\mu})\|_2^{\,2}$ with initial values $\boldsymbol{\mu}_{\cdot j}^{*(K_2+1)}$ using dog-leg trust-region method, which is implemented with "fsolve" function in MATLAB. The returned vector is named as $\boldsymbol{\mu}_{\cdot j}^{*(K_2+2)}$.

6) Optimize to all the BSR constraints by performing **2)** without **2-2-3-1)** and **2-2-3-3)** using objective function $h = h_1(O_{\mathrm{fix}}, O_{\mathrm{basin}}, O_{\mathrm{relax}})$ using initial vector $\boldsymbol{\mu}_{\cdot j}^{*(1)} \leftarrow \boldsymbol{\mu}_{\cdot j}^{*(K_2+2)}$ and iterate for $k_2 = 2, \ldots, K_3 - 1$ to give $\boldsymbol{\mu}_{\cdot j}^{*(K_3)}$, where K_3 defines a iteration limit. We termed the steps from **2)** to **6)** as global L-BFGS (g-LBFGS) method.

7) Repeat g-LBFGS.

For $k_\mathrm{b} = 2, 3$:

7-1) Renew and expand observation points $\boldsymbol{X}^{(k_b)} = \left\{\boldsymbol{x}_m \mid x_{m,n}^{(k_b)} = (1 + a\, d_\mathrm{max})x^*{}_j \text{ for } m = 1,2,\dots,N_\mathrm{obs},\ j = 1,2,\dots,N_\mathrm{phase}.\right\}$, where a was taken from a uniform distribution over $[-(0.4 \times k_\mathrm{b} - 0.2)^2, (0.4 \times k_\mathrm{b} - 0.2)^2]$.

7-2) $\boldsymbol{\mu}_{\cdot j}^{*(\mathbf{1})} \leftarrow \boldsymbol{\mu}_{\cdot j}^{*(K_3)}$, and perform **2)** to **6)**.

End for.

8) Iterate gCNM until convergence of the obtained parameter distribution.

8-0) Set $k_\mathrm{gCNM} = 1$, where k_gCNM is iteration number of entire loop of gCNM. Prepare an empty matrix $\boldsymbol{C}_{\mathrm{final},\, k_\mathrm{gCNM}} \in \boldsymbol{R}^{N_\mathrm{p} \times 0}$.

8-1) Store all the optimized parameter vectors.

For $j = 1,2,\dots,N_\mathrm{pc}$:

$$\boldsymbol{C}^{\mathrm{final},\, k_\mathrm{gCNM}} \leftarrow \left[\boldsymbol{C}^{\mathrm{final},\, k_\mathrm{gCNM}}, \boldsymbol{\mu}_{\cdot j}^{*(K_3)}\right]$$

End for.

8-2) Iterate gCNM and check convergence.

8-2-1) $k_\mathrm{gCNM} \leftarrow k_\mathrm{gCNM} + 1$.

8-2-2) Perform **2)** to **7).**

8-2-3) Store all the optimized parameter vectors.

For $j = 1,2,\dots,N_\mathrm{pc}$:

$$\boldsymbol{C}^{\mathrm{final},\, k_\mathrm{gCNM}} \leftarrow \left[\boldsymbol{C}^{\mathrm{final},\, k_\mathrm{gCNM}-\mathbf{1}}, \boldsymbol{\mu}_{\cdot j}^{*(K_3)}\right]$$

End for.

8-2-4) Perform statistical test:

Calculate following P value:

$$P = \max_{i=1,2,\dots,N_\mathrm{p}} WRStest(\boldsymbol{C}^{\mathrm{final},\, k_\mathrm{gCNM}}{}_{i\cdot}, \boldsymbol{C}^{\mathrm{final},\, k_\mathrm{gCNM}-1}{}_{i\cdot}),$$

where $WRStest(\boldsymbol{a}, \boldsymbol{b})$ is a function returns Wilcoxon rank sum test P-value between vectors $\boldsymbol{a}$ and $\boldsymbol{b}$, and $\boldsymbol{C}^\mathrm{final}{}_{i\cdot}$ is a i th row vector of $\boldsymbol{C}^\mathrm{final}$.

8-2-5) Check convergence criteria for median shift:

If $P < \alpha$, where α is a significance level:

Perform another gCNM iteration by going back to **8-2-1)**.

End if.

In this study, we used $\alpha = 0.1$.

8-2-6) Calculate inclusion rate R:

$$R = \min_{i=1,2,\dots,N_\mathrm{p}} ICR(\boldsymbol{\mu}_{i\cdot}^{*(K_3)}, \boldsymbol{C}^{\mathrm{final},\, k_\mathrm{gCNM}-1}{}_{i\cdot}),$$

where $ICR(\boldsymbol{a}, \boldsymbol{b})$ is a function returns a rate of element of vector $\boldsymbol{a}$ included in the interval $\left[\min_i b_i, \max_i b_i\right]$ and b_i is i th element of vector $\boldsymbol{b}$.

8-2-7) Check convergence criteria for inclusion rate:

If $R < \beta$, where β is a threshold:

Perform another gCNM iteration by going back to **8-2-1)**.

Else:

Finish. $\boldsymbol{C}^{\mathrm{final},\, k_\mathrm{gCNM}}$ is the final matrix consisting of finally obtained parameter vectors (sets).

End if.

In this study, we used $\beta = 0.99$.

Note for symbols used in the algorithm.

$\boldsymbol{\mu}$	a vector consisting of model parameters
$\boldsymbol{x}$	a vector consisting of a state in the phase space
N_{t}	the number of constraints (targets)
l	an index for constraint
N_{p}	the dimension of the parameter space
i	an index for parameter
N_{pc}	the number of parameter sets in the cluster
j	an index for parameter vector in the cluster
N_{div}	the subdividing number of the cluster
N_{psc}	the number of parameter sets in the sub-cluster
k_{sc}	an index for subcluster in CNM
j'	an index for parameter vector in the sub-cluster
N_{obs}	the number of observation points for calculating O_basin
m	an index for observation points
N_{phase}	the dimension of phase space
n	an index for phase space dimension
$\boldsymbol{C}$	matrix consisting of parameter vectors (= cluster of parameter sets)
$\boldsymbol{C}_{store}$	matrix consisting of parameter vectors moderately close to the target condition
$\boldsymbol{C}^{final}$	matrix consisting of finally obtained parameter vectors
k_b	an index defining the broadness of distribution of observation points
k_{gCNM}	an iteration number of gCNM
$\boldsymbol{y}^*$	a vector consisting of target values for objective functions
$\boldsymbol{y}$	a vector consisting of objective function values
$\boldsymbol{Y}$	a matrix consisting of objective function value vectors inCNM
$\boldsymbol{A}$	a slope matrix of hyperplane in CNM
$\boldsymbol{s}$	a vector for updating in optimization
$\boldsymbol{g}$	a gradient vector in LBFGS optimization
