# Supplementary material for: Utility of constraints reflecting system stability on analyses for biological models
Source: PLoS Comput Biol. 2022 Sep 9;18(9):e1010441. doi: 10.1371/journal.pcbi.1010441 (PMC9491612; doi:10.1371/journal.pcbi.1010441)
Supplement: S4 Information — (PDF) [file pcbi.1010441.s013.pdf]

S4 Information

Computation report

Following information is described.

<Model name>

The name of model.

<Parameter Setting>

Search region: Preset search region in parameter space.

Weight of objective function

w_fix: The exponential weight on O_fix. In the integrative objective, O_fix^{w_fix} is used. Usually, w_fix = 2 shows good performance as a rule of thumb.

w_relax: The exponential weight on O_relax. In the integrative objective, O_relax^{w_relax} is used. Usually, w_relax = 1 ~ 2 shows good performance as a rule of thumb.

w_basin: The exponential weight on O_basin. In the integrative objective, O_basin^{w_basin} is used. Usually, w_basin set to same value with w_relax shows good performance as a rule of thumb.

n_sample_per_cluster: A number of parameter sets in one cluster. A default value is 400.

n_div: A number of clusters in CNM. A default value is 2.

(Total number of parameter sets in a TEAPS loop is n_sample_per_cluster times n_div.)

target_relax: The target value of the upper threshold of maximum nonzero eigenvalue of Jacobian matrix at the target fixed points (max of Eig_relax).

target_max_basin_size: This value defines a region in the phase space where observation points are generated. The values of observation points in each dimension are randomly generated between the value of target fixed point times (1 - target_max_basin_size) and that the value times (1 + target_max_basin_size) in each dimension.

<computation environment>

CPU Core (thread) number: The number of threads of computer used in calculation.

Max CPU clock: The max clock value of CPU.

Parallel number: The number of parallel calculations implemented in MATLAB.

<Result>

computation time: The time until TEAPS completion.

Number of TEAPS loops: The number of TEAPS loops until distribution convergence.
Total number of seed parameter set: The total number of parameter sets seeded in TEAPS with consideration of loop number. (This value can be calculated by n_sample_per_cluster × n_div × number of loops.)
Number of parameter sets met BSR: The number of parameter sets met BSR constraint.
Evaluations of objective function values
The following factors related in objective function against finally obtained parameter sets were plotted.
$||dF||_2$: Norm of time derivative of f(u,x) is used for assessment of O_fix. This value should be zero (or almost zero if considering computation tolerance).
Max Eig_relax: This value is used for assessment of O_relax. Aiming to adjust time scale, TEAPS aim to optimize this value less than the target_relax but parameter sets showing a negative value for the max of Eig_relax are consistent with BSR since this is consistent with convergence to the target fixed point.
Max Eig_obs: The max eigenvalues for Jacobian matrix at all observation points. This value is used for assessment of O_basin and should be zero or less (or almost zero or less if considering computation tolerance) to support the contraction around the fixed point by the dynamics, which is consistent with BSR.
Time course plot
The time courses of each model given by the obtained parameter sets have been simulated. A hundred parameter sets were randomly selected from the obtained parameter sets. The initial state for each simulation was randomly generated around the target stable points (xi = 1) with a maximum perturbation size of 10 % for each entity (variable) in consideration of structural constraints: Entities which could not be converged due to isolated structure are not perturbed (x3 in model T3 and x4 in model T8), and local mass balance was considered (x1 + x2 = 2 and x3 + x4 = 2 for models T5 and T6). Under this setting, the convergence toward xi = 1 was observed in most cases. Even in the cases where such convergence cannot be observed, the amount of each entity was not diverged but stayed near the target fixed point when the initial states were in the aimed stable region, $|xi - 1| \leq 0.1$, in most cases. The behavior observed here is consistent with our design of objective function which allows the neutrally stable fixed point around the target.

<Comment>
Concise comments on the obtained results.

<Model name>

T1

<Parameter Setting>

Search region: 0.5 ～ 5×10^4 for x(5) (input flux), and 5×10^{-3} ～ 5×10^4 for others.

Weight of objective function

w_fix: 2.

w_relax: 1

w_basin: 1

n_sample_per_cluster: 400

n_div: 2

target_relax: -0.3

target_max_basin_size: 0.1

<computation environment>

CPU Core (thread) number: 48

Max CPU clock: 2.7 GHz

Parallel number: 46

<Result>

computation time: 5 min 3.268 sec

Number of TEAPS loops: 2

Total number of seed parameter set: 1600

Number of parameter sets met BSR: 1556

Evaluations of objective function values

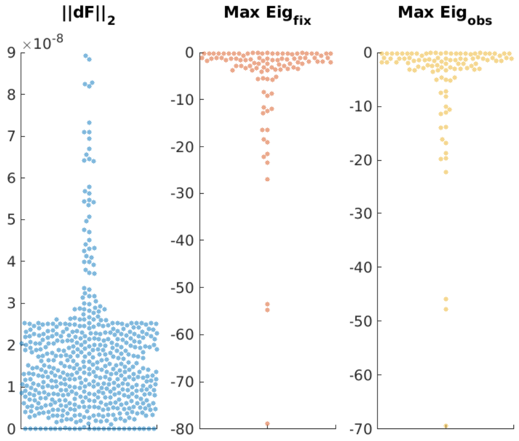

Time course plot

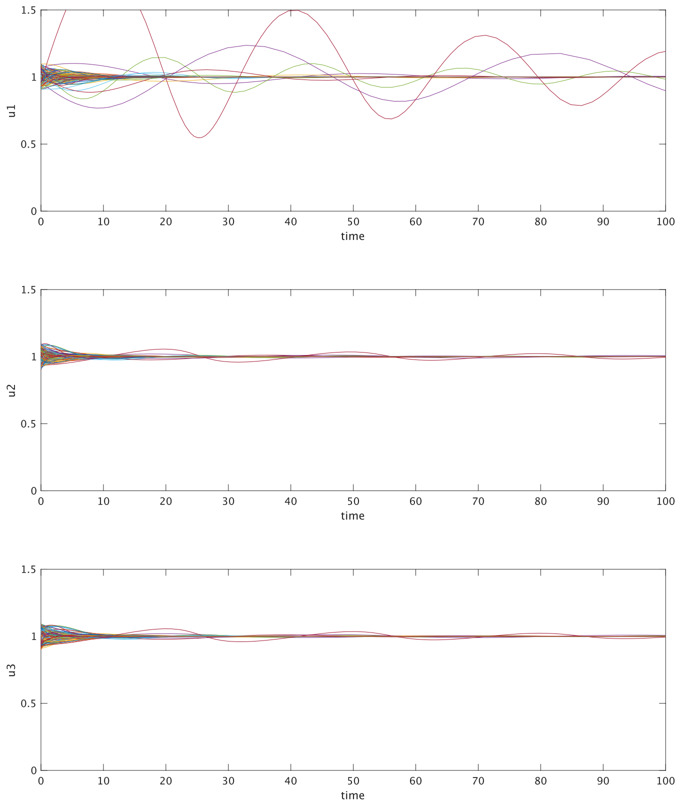

<Comment>

The values of l2-norm of dF for each parameter set were judged as zero since the value is quite small.

<Model name>

T2

<Parameter Setting>

Search region: 0.5 ～ 5×10^4 for x(2) (input flux), and 5×10^{-3} ～ 5×10^4 for others.

Weight of objective function

w_fix: 2.

w_relax: 2

w_basin: 2

n_sample_per_cluster: 400

n_div: 2

target_relax: -0.3

target_max_basin_size: 0.1

<computation environment>

CPU Core (thread) number: 24

Max CPU clock: 2.7 GHz

Parallel number: 22

<Result>

computation time: 25 min 8.864 sec

Number of TEAPS loops: 3

Total number of seed parameter set: 2400

Number of parameter sets met BSR: 2369

Evaluations of objective function values

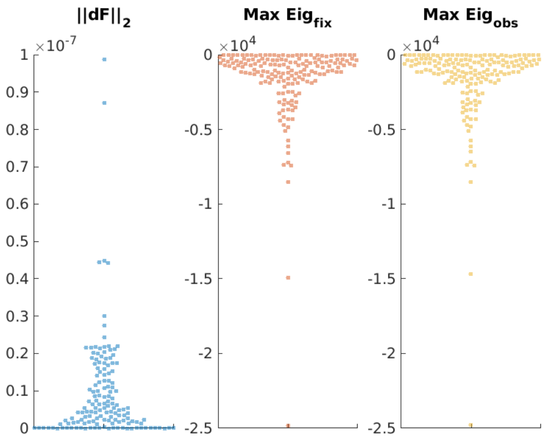

Time course plot

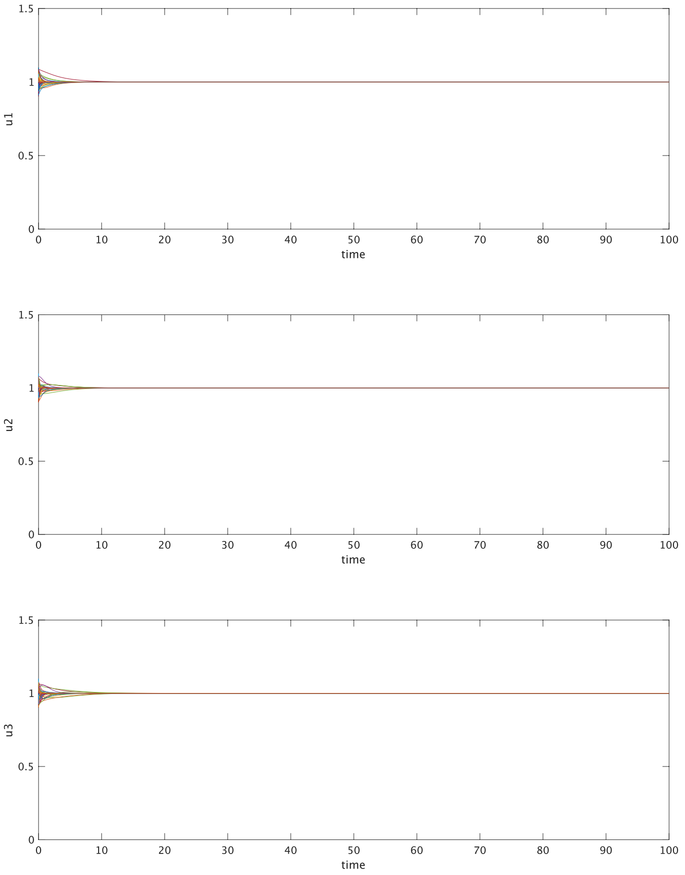

<Comment>

The values of l2-norm of dF for each parameter set were judged as zero since the value is quite small.

<Model name>

T3

<Parameter Setting>

Search region: 0.5 ～ 5×10^4 for x(5) (input flux), and 5×10^{-3} ～ 5×10^4 for others.

Weight of objective function

w_fix: 2.

w_relax: 2

w_basin: 2

n_sample_per_cluster: 400

n_div: 2

target_relax: -0.3

target_max_basin_size: 0.1

<computation environment>

CPU Core (thread) number: 48

Max CPU clock: 2.7 GHz

Parallel number: 46

<Result>

computation time: 9 min 4.4679 sec

Number of TEAPS loops: 2

Total number of seed parameter set: 1600

Number of parameter sets met BSR: 1082

Evaluations of objective function values

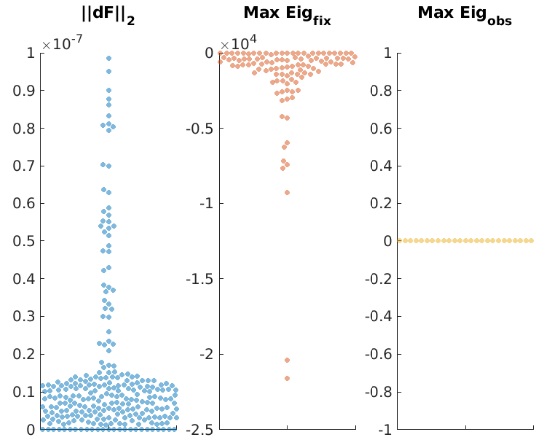

Time course plot

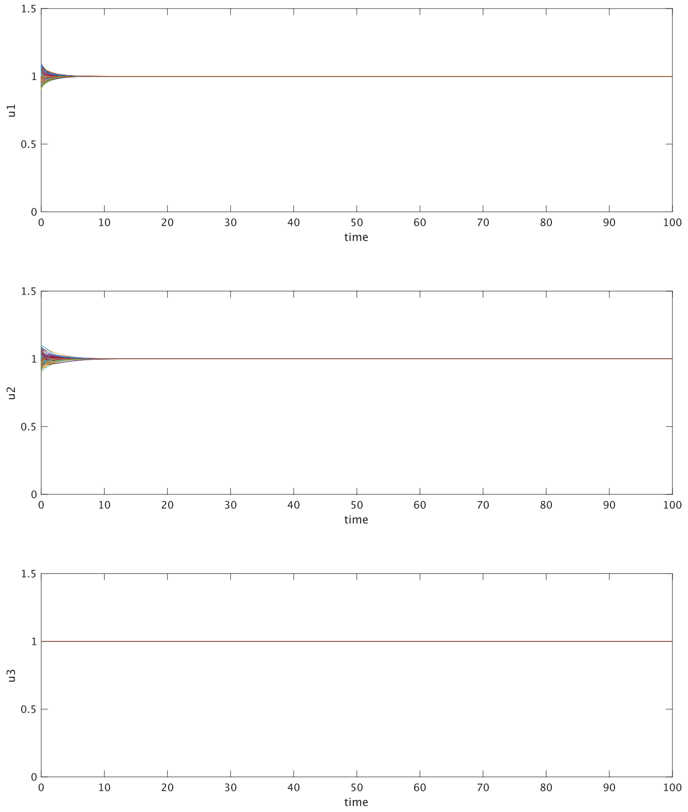

<Comment>

The values of l2-norm of dF for each parameter set were judged as zero since the value is quite small.

<Model name>

T4

<Parameter Setting>

Search region: 0.5 ～ 5×10^4 for x(8) (input flux), and 5×10^{-3} ～ 5×10^4 for others.

Weight of objective function

w_fix: 2.

w_relax: 2

w_basin: 2

n_sample_per_cluster: 400

n_div: 2

target_relax: -0.3

target_max_basin_size: 0.1

<computation environment>

CPU Core (thread) number: 48

Max CPU clock: 2.7 GHz

Parallel number: 46

<Result>

computation time: 11 min 27.173 sec

Number of TEAPS loops: 3

Total number of seed parameter set: 2400

Number of parameter sets met BSR: 1842

Evaluations of objective function values

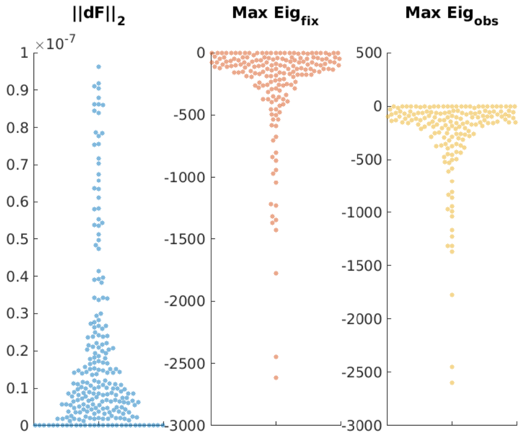

Time course plot

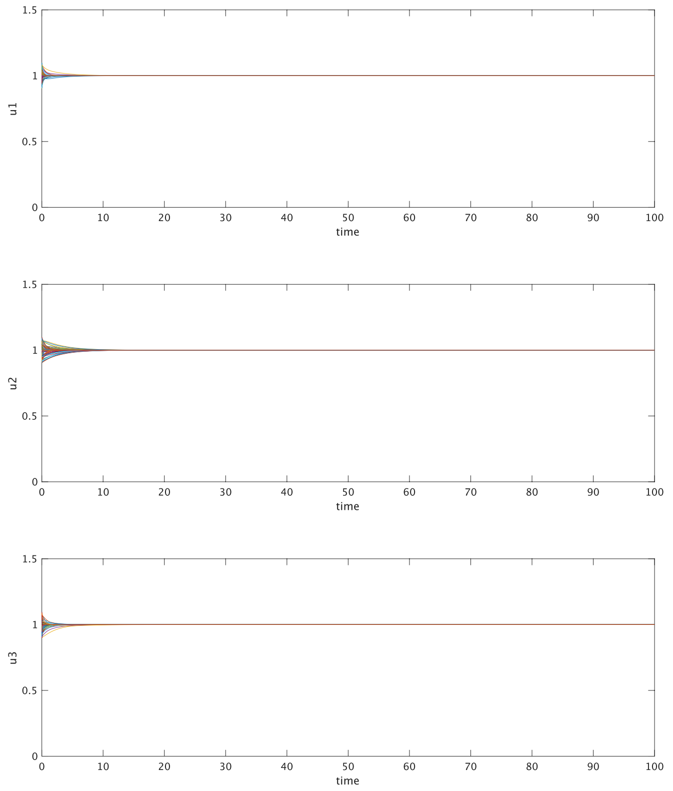

<Comment>

The values of l2-norm of dF for each parameter set were judged as zero since the value is quite small.

<Model name>

T5

<Parameter Setting>

Search region: 0.5 ～ 5×10^4 for x(5) (input flux), and 5×10^{-3} ～ 5×10^4 for others.

Weight of objective function

w_fix: 2.

w_relax: 2

w_basin: 2

n_sample_per_cluster: 400

n_div: 2

target_relax: -0.3

target_max_basin_size: 0.1

<computation environment>

CPU Core (thread) number: 24

Max CPU clock: 2.7 GHz

Parallel number: 22

<Result>

computation time: 28 min 42.563 sec

Number of TEAPS loops: 2

Total number of seed parameter set: 1600

Number of parameter sets met BSR: 1341

Evaluations of objective function values

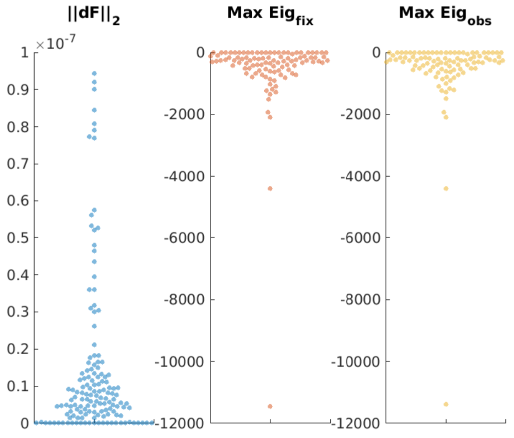

Time course plot

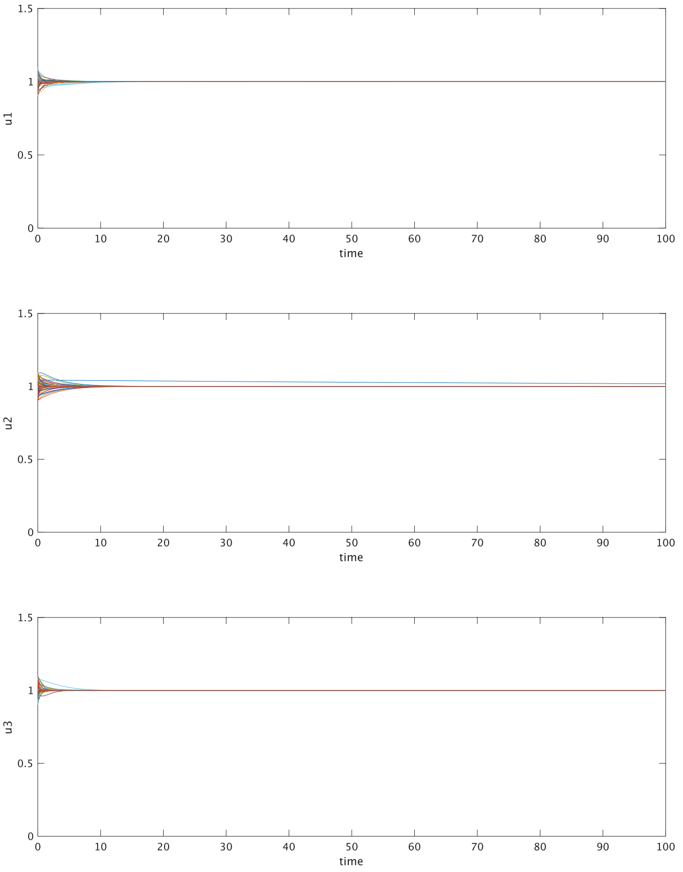

<Comment>

The values of l2-norm of dF for each parameter set were judged as zero since the value is quite small.

<Model name>

T6

<Parameter Setting>

Search region: 5×10^{-3} ～ 5×10^{4} for all parameters.

Weight of objective function

w_fix: 2.

w_relax: 1.2

w_basin: 1.2

n_sample_per_cluster: 400

n_div: 2

target_relax: -0.3

target_max_basin_size: 0.1

<computation environment>

CPU Core (thread) number: 48

Max CPU clock: 2.7 GHz

Parallel number: 46

<Result>

computation time: 12 min 22.206 sec

Number of TEAPS loops: 2

Total number of seed parameter set: 1600

Number of parameter sets met BSR: 1192

Evaluations of objective function values

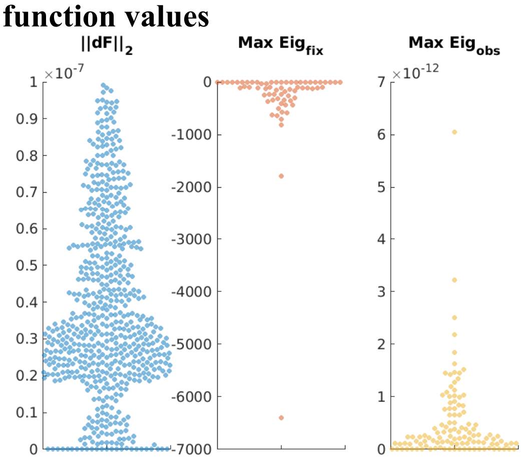

Time course plot

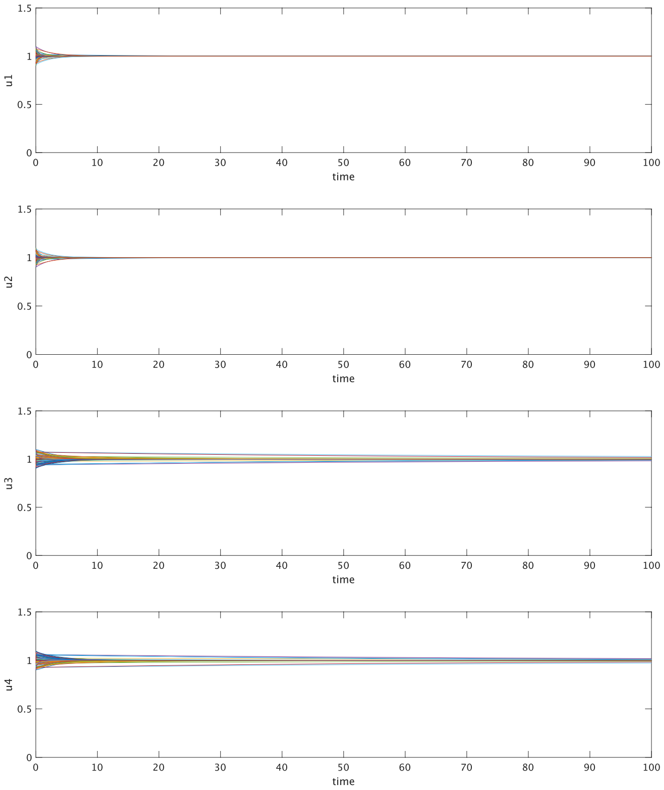

<Comment>

The values of l2-norm of dF and "max Eig_obs" for each parameter set were judged as zero since the value is quite small.

<Model name>

T7

<Parameter Setting>

Search region: 5×10^{-3} ～ 5×10^{4} for all parameters.

Weight of objective function

w_fix: 2.

w_relax: 1.2

w_basin: 1.2

n_sample_per_cluster: 400

n_div: 2

target_relax: -0.3

target_max_basin_size: 0.1

<computation environment>

CPU Core (thread) number: 48

Max CPU clock: 2.7 GHz

Parallel number: 46

<Result>

computation time: 31 min 32.687 sec

Number of TEAPS loops: 5

Total number of seed parameter set: 4000

Number of parameter sets met BSR: 2882

Evaluations of objective function values

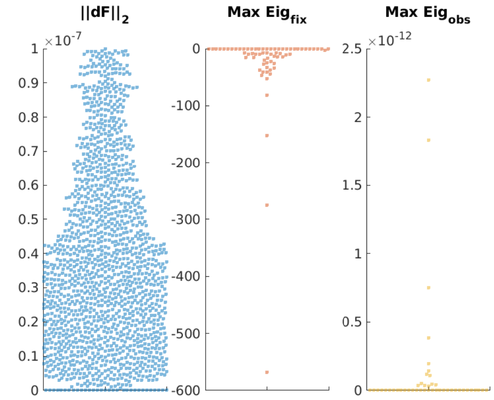

Time course plot

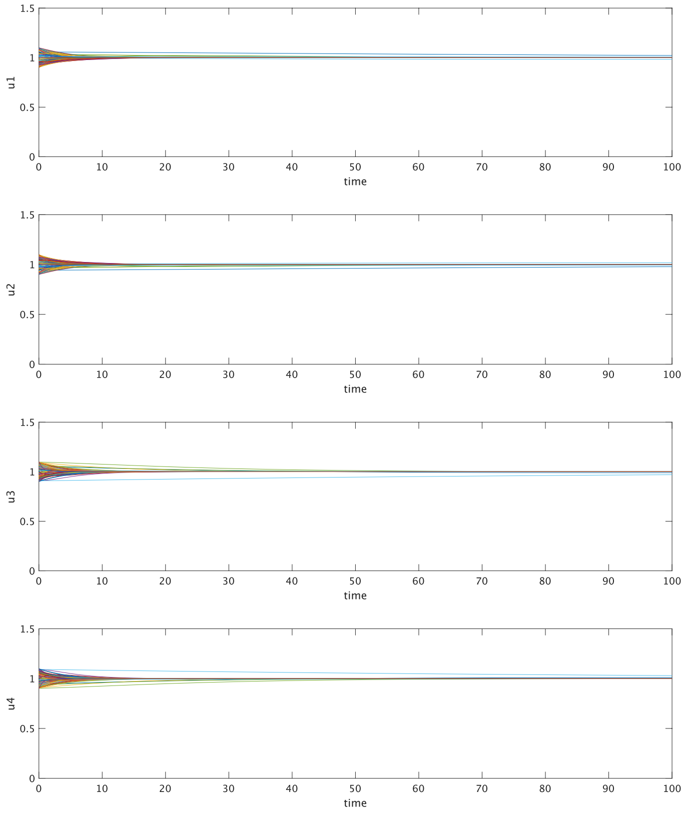

<Comment>

The values of l2-norm of dF and “max Eig_obs” for each parameter set were judged as zero since the value is quite small.

<Model name>

T8

<Parameter Setting>

Search region: 5×10^{-3} ～ 5×10^{4} for all parameters.

Weight of objective function

w_fix: 2.

w_relax: 2

w_basin: 2

n_sample_per_cluster: 400

n_div: 2

target_relax: -0.3

target_max_basin_size: 0.1

<computation environment>

CPU Core (thread) number: 24

Max CPU clock: 2.7 GHz

Parallel number: 42

<Result>

computation time: 1 hr 38 min 58.741 sec

Number of TEAPS loops: 2

Total number of seed parameter set: 1600

Number of parameter sets met BSR: 635

Evaluations of objective function values

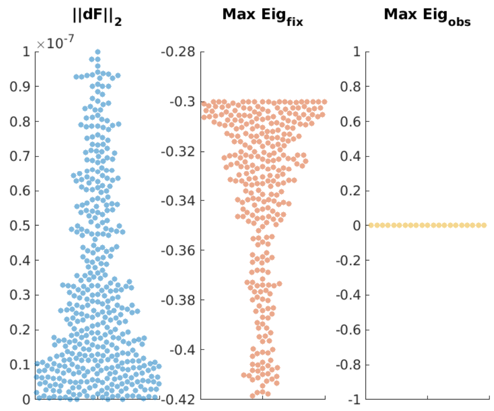

Time course plot

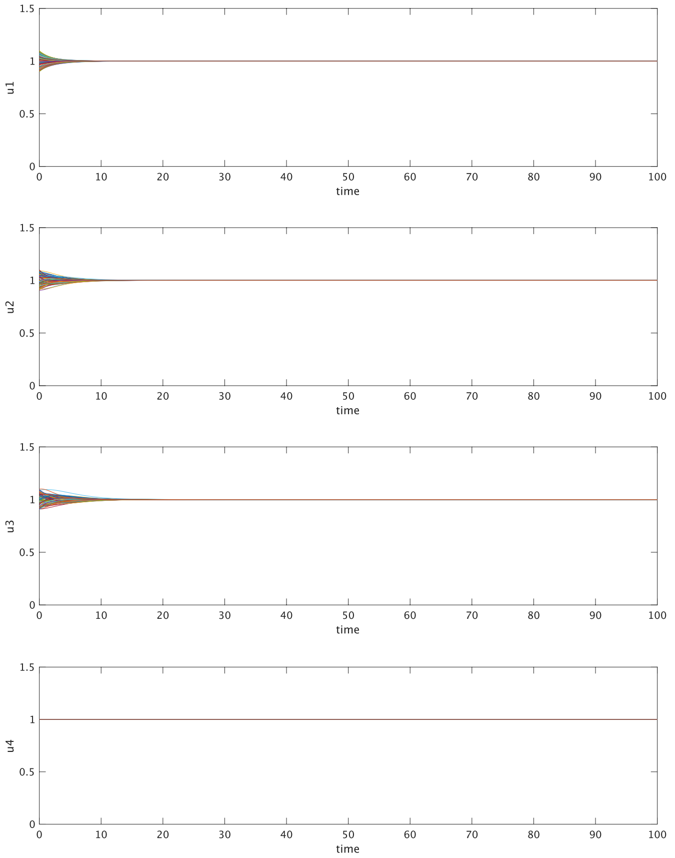

<Comment>

The values of l2-norm of dF for each parameter set were judged as zero since the value is quite small.

<Model name>

NF-kappaB

<Parameter Setting>

Search region: 1×10^{-7} ～ 1×10^{2} for all parameters.

Weight of objective function

w_fix: 2.

w_relax: 1

w_basin: 1

n_sample_per_cluster: 400

n_div: 2

target_relax: -1×10^{-4}

target_max_basin_size: 0.1

<computation environment>

CPU Core (thread) number: 24

Max CPU clock: 2.7 GHz

Parallel number: 42

<Result>

computation time: 1 hr 56 min 33.681 sec

Number of TEAPS loops: 2

Total number of seed parameter set: 1200

Number of parameter sets met BSR: 1095

Evaluations of objective function values

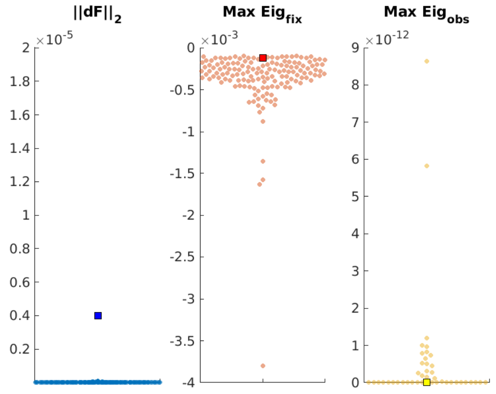

Note that the box markers indicate the values when calculated with the reported parameter set.

Time course plot

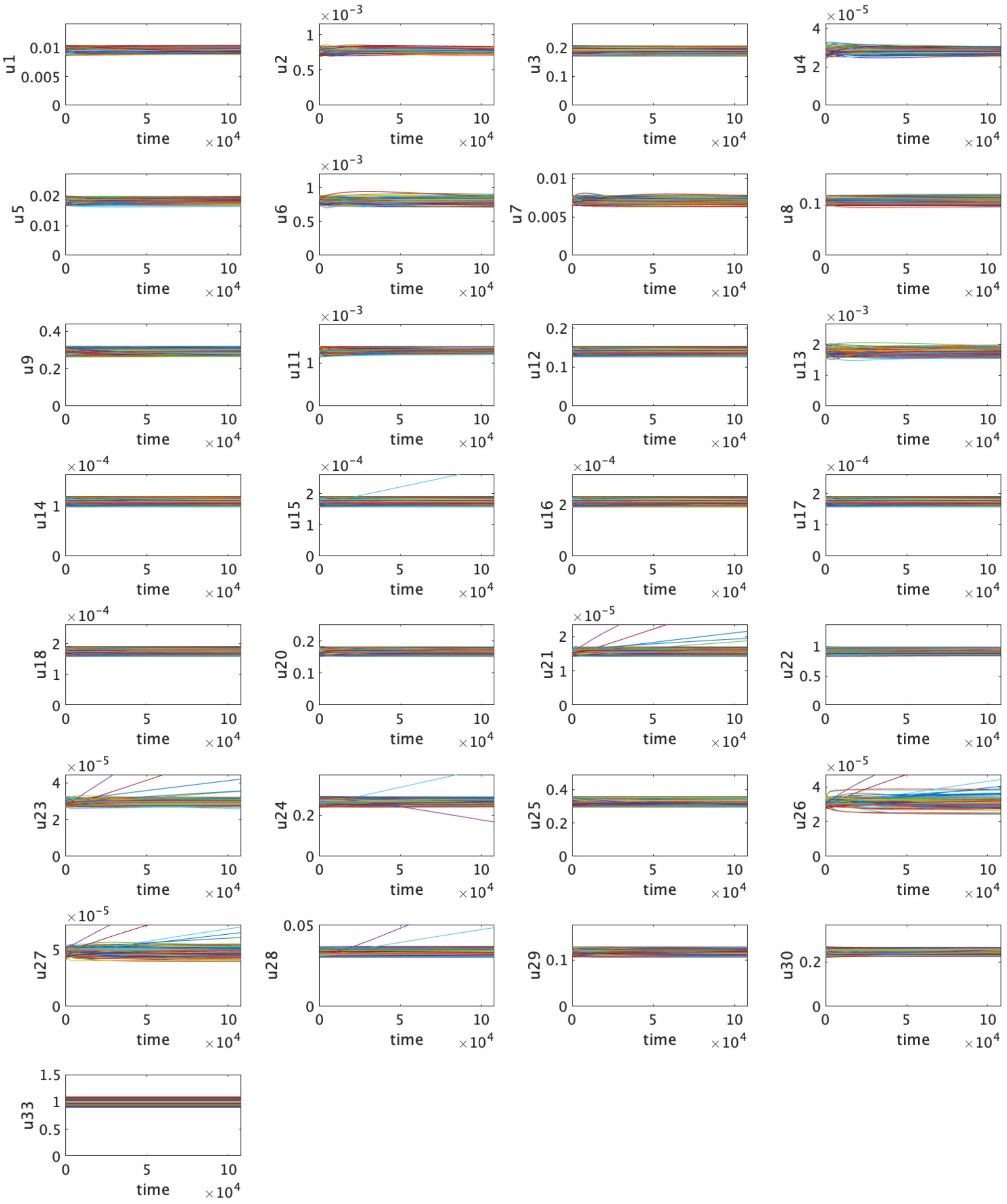

<Comment>

The values of l2-norm of dF and “max Eig_obs” for each parameter set were judged as zero since the value is quite small.

<Model name>

Arachidonic acid pathway model

<Parameter Setting>

Search region: 1×10^{-3} ～ 1×10^{20} for all parameters.

Weight of objective function

w_fix: 2.

w_relax: 1

w_basin: 1

n_sample_per_cluster: 400

n_div: 2

target_relax: -0.3

target_max_basin_size: 0.1

<computation environment>

CPU Core (thread) number: 24

Max CPU clock: 2.7 GHz

Parallel number: 42

<Result>

computation time: 2 d 15 hr 56 min 56.889 sec

Number of TEAPS loops: 5

Total number of seed parameter set: 4000

Number of parameter sets met BSR: 1391

Evaluations of objective function values

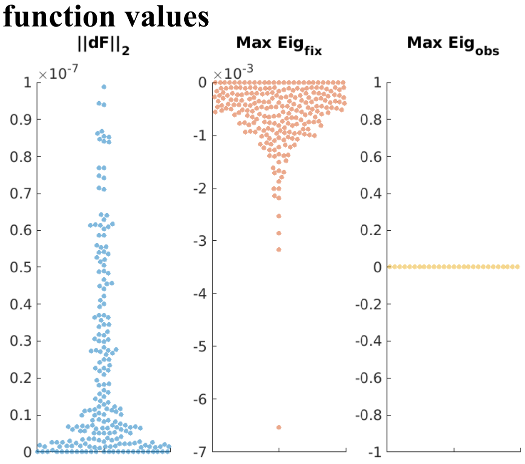

Time course plot

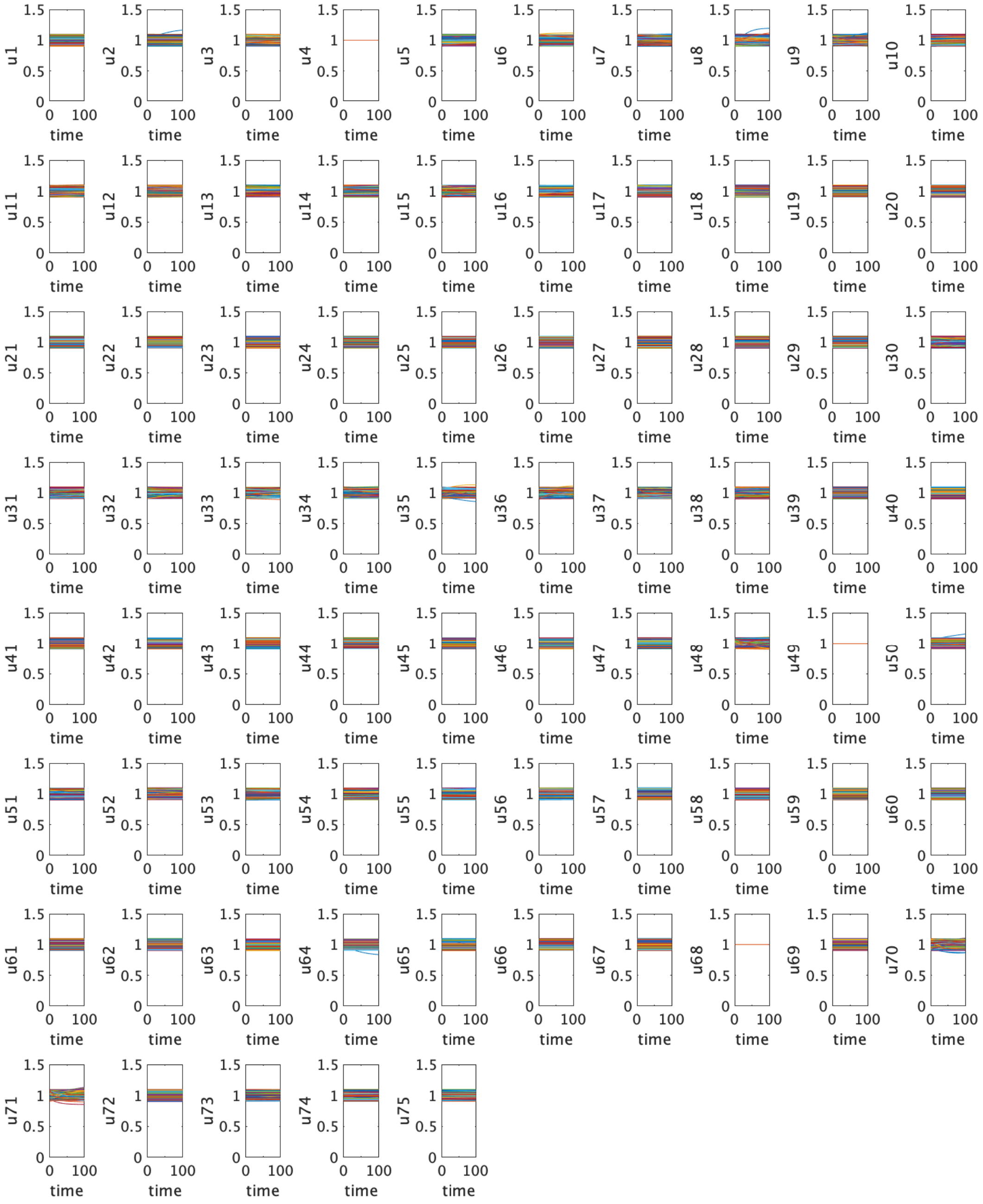

<Comment>

The values of l2-norm of dF for each parameter set were judged as zero since the value is quite small. The values of “max Eig_relax” was higher than the target value but take negative value which is consistent with BSR concept.
